# Effect of scheduled antimicrobial and nicotinamide treatment on linear growth in children in rural Tanzania: A factorial randomized, double-blind, placebo-controlled trial

**DOI:** 10.1371/journal.pmed.1003617

**Published:** 2021-09-28

**Authors:** Mark D. DeBoer, James A. Platts-Mills, Sarah E. Elwood, Rebecca J. Scharf, Joann M. McDermid, Anne W. Wanjuhi, Samwel Jatosh, Siphael Katengu, Tarina C. Parpia, Elizabeth T. Rogawski McQuade, Jean Gratz, Erling Svensen, Jonathan R. Swann, Jeffrey R. Donowitz, Paschal Mdoe, Sokoine Kivuyo, Eric R. Houpt, Estomih Mduma

**Affiliations:** 1 Department of Pediatrics, University of Virginia, Charlottesville, Virginia, United States of America; 2 Division of Infectious Diseases & International Health, University of Virginia, Charlottesville, Virginia, United States of America; 3 Haydom Global Health Research Centre, Haydom Lutheran Hospital, Haydom, Tanzania; 4 Haukeland University Hospital, Bergen, Norway; 5 School of Human Development and Health, Faculty of Medicine, University of Southampton, Southampton, United Kingdom; 6 Department of Surgery & Cancer, Imperial College London, London, United Kingdom; 7 Division of Infectious Disease, Children’s Hospital of Richmond at Virginia Commonwealth University, Richmond, Virginia, United States of America; 8 National Institute for Medical Research, Muhimbili Medical Research Centre, Dar es Salaam, Tanzania; Instituto de Salud Global de Barcelona, SPAIN

## Abstract

**Background:**

Stunting among children in low-resource settings is associated with enteric pathogen carriage and micronutrient deficiencies. Our goal was to test whether administration of scheduled antimicrobials and daily nicotinamide improved linear growth in a region with a high prevalence of stunting and enteric pathogen carriage.

**Methods and findings:**

We performed a randomized, 2 × 2 factorial, double-blind, placebo-controlled trial in the area around Haydom, Tanzania. Mother–child dyads were enrolled by age 14 days and followed with monthly home visits and every 3-month anthropometry assessments through 18 months. Those randomized to the antimicrobial arm received 2 medications (versus corresponding placebos): azithromycin (single dose of 20 mg/kg) at months 6, 9, 12, and 15 and nitazoxanide (3-day course of 100 mg twice daily) at months 12 and 15. Those randomized to nicotinamide arm received daily nicotinamide to the mother (250 mg pills months 0 to 6) and to the child (100 mg sachets months 6 to 18). Primary outcome was length-for-age z-score (LAZ) at 18 months in the modified intention-to-treat group. Between September 5, 2017 and August 31, 2018, 1,188 children were randomized, of whom 1,084 (*n* = 277 placebo/placebo, 273 antimicrobial/placebo, 274 placebo/nicotinamide, and 260 antimicrobial/nicotinamide) were included in the modified intention-to-treat analysis. The study was suspended for a 3-month period by the Tanzanian National Institute for Medical Research (NIMR) because of concerns related to the timing of laboratory testing and the total number of serious adverse events (SAEs); this resulted in some participants receiving their final study assessment late. There was a high prevalence of stunting overall (533/1,084, 49.2%). Mean 18-month LAZ did not differ between groups for either intervention (mean LAZ with 95% confidence interval [CI]: antimicrobial: −2.05 CI −2.13, −1.96, placebo: −2.05 CI −2.14, −1.97; mean difference: 0.01 CI −0.13, 0.11, *p* = 0.91; nicotinamide: −2.06 CI −2.13, −1.95, placebo: −2.04 CI −2.14, −1.98, mean difference 0.03 CI −0.15, 0.09, *p* = 0.66). There was no difference in LAZ for either intervention after adjusting for possible confounders (baseline LAZ, age in days at 18-month measurement, ward, hospital birth, birth month, years of maternal education, socioeconomic status (SES) quartile category, sex, whether the mother was a member of the Datoga tribe, and mother’s height). Adverse events (AEs) and SAEs were overall similar between treatment groups for both the nicotinamide and antimicrobial interventions. Key limitations include the absence of laboratory measures of pathogen carriage and nicotinamide metabolism to provide context for the negative findings.

**Conclusions:**

In this study, we observed that neither scheduled administration of azithromycin and nitazoxanide nor daily provision of nicotinamide was associated with improved growth in this resource-poor setting with a high force of enteric infections. Further research remains critical to identify interventions toward improved early childhood growth in challenging conditions.

**Trial registration:**

ClinicalTrials.gov NCT03268902.

## Abstract 2

In a randomized trial, Mark DeBoer and colleagues investigate the effect of antimicrobial and nicotinamide interventions on child growth.

## Author summary

**Why was this study done?:**

- Prior studies in resource-poor settings have revealed a strong association between carriage of bacterial and protozoal enteric pathogens and early life growth deficits.
- Preclinical and observational studies have suggested that deficiencies in the tryptophan–niacin–nicotinamide adenine dinucleotide (NAD+) pathway were associated with impaired growth and development.
- Clinical trials to evaluate the impact of either (a) regularly scheduled antimicrobial therapy targeting bacterial and protozoal pathogens; or (b) supplementation of the tryptophan–niacin–NAD+ pathway on child growth and development have not been performed.

**What did the researchers do and find?:**

- In this double-blind placebo-controlled, randomized controlled trial (RCT), we administered in a 2 × 2 factorial manner scheduled antimicrobials (azithromycin and nitazoxanide) and/or daily nicotinamide to a cohort of 1,188 children 0 to 18 months old, with a primary outcome of length-for-age z-score (LAZ) at 18 months.
- We found that neither of these interventions altered growth in these children, with a final prevalence of stunting in all subgroups of 47% to 52%.

**What do these findings mean?:**

- Scheduled administration of antimicrobials in an area with a high prevalence of pathogen carriage did not increase childhood growth, suggesting that either that this approach did not adequately reduce pathogen carriage, that reduced enteric pathogen carriage does not improve growth, or that other health challenges predominated.
- Daily provision of nicotinamide also did not alter growth, suggesting against deficiencies in the tryptophan–niacin–NAD+ pathway as a key cause of growth failure in this area with a maize-predominant diet.

## Introduction

Despite worldwide improvements in early childhood mortality over the past decades, poor early childhood growth in resource-poor settings remains a major challenge, with over one-third of children experiencing stunting of linear growth [1]. The causes of early growth failure are varied and include nutrition insufficiencies and enteric infections [2]. In turn, poor growth is frequently associated with lower developmental attainment [3], suggesting that these challenges contribute to loss of school readiness and future human capital [4].

An early and high burden of infection with bacterial and protozoal enteric pathogens has been strongly associated with growth deficits in children less than 2 years of age [5,6], including enteroaggregative *Escherichia coli*, *Campylobacter* species, *Shigella*, *Giardia*, and *Cryptosporidium* [7]. The potential that scheduled antimicrobial treatment may improve linear growth was provided by a meta-analysis of randomized controlled trials (RCTs) administering different antimicrobials to children, revealing that children receiving antimicrobials grew an additional 0.04 cm/month [8]; significant differences in growth have not been seen in most individual trials, such as a more recent cluster randomized trial of mass azithromycin distribution for trachoma [9]. Nitazoxanide is an antiprotozoal agent with activity against *Giardia* and *Cryptosporidium*. Treatment of *Cryptosporidium* infection with nitazoxanide in Zambia was associated with reduced mortality, but effects on linear growth were unstudied [10]. Therefore, it remains unclear whether administration of antimicrobials in an area with a high enteric pathogen burden is associated with improved linear growth in infants and young children.

Regional diets are susceptible to micronutrient deficiencies, and targeted supplementation with individual micronutrients in some studies has been associated with improvements in height [11]. Many resource-poor areas around the world rely predominantly on maize-based diets, which are low in the essential amino acid tryptophan. Tryptophan, via the kynurenine pathway, is an important precursor for the de novo synthesis of nicotinamide adenine dinucleotide (NAD+), an essential cofactor with a key role in a wide variety of metabolic processes including energy metabolism vital for growth and immune responses [12]. Cells can also generate NAD+ from niacin in the form of nicotinic acid via the Preiss–Handler pathway or of nicotinamide via the salvage pathway. Deficiencies in niacin are associated with pellagra and can further deplete the already limited tryptophan pool to maintain NAD+ availability. Lower serum levels of tryptophan have been associated with reduced stature in young children, while a higher ratio of kynurenine to tryptophan was associated with worsened response to vaccines [13]. Greater excretion of *N-*methylnicotinamide, a urinary biomarker of nicotinamide and NAD+ availability, was also associated with greater growth in stunted infants 6 months later [14]. Together, these data suggest that supplementing this pathway may have potential benefits for improving health in early childhood.

The area around Haydom, Tanzania is a rural setting where most families practice maize-based subsistence farming with a single agricultural harvest per annum, only have access to unimproved water sources, and have an income below 58,000 Tanzania shillings (approximately US$25) per month [15–17]. Haydom has a very low prevalence (<2%) of maternal HIV, and, located at over 1,500 feet elevation, almost no malaria [15]. A recent cohort study revealed that despite having only 15% stunting at birth, 70% of children followed in the Haydom area were stunted at 18 months, and their first 2 years of life were marked by carriage of multiple intestinal pathogens [6,7]. Because of the interrelatedness of enteric infections and nutrition—in which infections contribute to poor nutrient absorption and malnutrition contributes to susceptibility to infections [2]—we conducted a trial of intervention with (1) scheduled antimicrobials (azithromycin at 6, 9, 12, and 15 months of age and nitazoxanide at 12 and 15 months of age); and (2) daily nicotinamide in an area with a high incidence of postnatal stunting and high carriage of intestinal pathogens. We hypothesized that (1) periodic administration of azithromycin and nitazoxanide and (2) daily nicotinamide for the first 18 months would each increase length-for-age z-score (LAZ) at 18 months.

## Methods

### Study design

The study design for the Early Life Interventions for Childhood Growth and Development in Tanzania (ELICIT) study and the baseline characteristics of its participants have been reported previously [17,18]. Briefly, ELICIT was a 2 × 2 factorial randomized, double-blind, placebo-controlled trial (RCT) based in the Haydom Global Health Research Centre at Haydom Lutheran Hospital in Haydom, Tanzania [15]. The study protocol was approved by the National Institute for Medical Research (NIMR) of Tanzania and the Tanzanian Food and Drug Administration (TFDA) and the Institutional Review Board at the University of Virginia. Study oversight was provided by FHI360 (North Carolina, United States of America). Mothers gave written informed consent to participate either during pregnancy or at the time of enrollment. This study is reported as per the Consolidated Standards of Reporting Trials (CONSORT) guideline (S1 CONSORT Checklist) [19].

### Participants

Prospective participants were identified by community health workers throughout the recruitment area as women who were either pregnant or had recently delivered. These pregnant women and mothers were then approached at their homes by field team members to inform them about the study and assess interest. Inclusion criteria were maternal age ≥18 years, child age ≤14 days, and the family’s stated intent to reside within a 25-km radius of Haydom Lutheran Hospital for the duration of the study (S1 Protocol). Exclusion criteria were multiple gestation, significant birth defect or neonatal illness, infant enrollment weight <1,500 g, and lack of intent to breastfeed.

### Randomization and masking

Individuals were allocated to one of 4 treatment groups (1:1:1:1) in a 2 × 2 factorial manner, such that participants received either nicotinamide plus placebo, placebo plus antimicrobials, both interventions, or both placebo (S1 Fig). Individuals were randomly allocated to treatment arms using permuted blocks with a block size of 8 and a reproducible seed by study author JPM. Lists of the allocation blocks were stored at the research center and provided to the field teams to randomize participants at enrollment; all investigators and participants remained blinded to treatment assignment until after study completion and the analysis of the prespecified primary outcome. Allocation for each study intervention was concealed by the manufacturer, who provided both the active treatment and matching placebo, with the allocation code held subsequently held in a sealed opaque envelope by a nonstudy investigator.

### Procedures

The antimicrobial intervention consisted of 2 different antimicrobial medications randomized together, such that participants received either azithromycin and nitazoxanide or a placebo version of each. Azithromycin or its corresponding placebo (both manufactured by Universal Corporation, Kenya) was administered as a single dose of 20 mg/kg of 200 mg/5 mL suspension by mouth to the infant at months 6, 9, 12, and 15. Nitazoxanide or placebo (both by Romark, Florida, USA) was administered as a 3-day course of 100 mg twice daily of 100 mg/5 mL suspension at months 12 and 15. The doses of azithromycin and the first dose of the nitazoxanide courses were administered by study personnel, while the remainder of the nitazoxanide course was administered by the mother or the caregiver.

The nicotinamide versus placebo (both manufactured by VITA-gen, New York, USA) was first provided to lactating mothers (250 mg or placebo tablets daily) during infant age 0 to 6 months and then to the child (100 mg or placebo sachets daily) from months 6 to 18. Mothers were instructed to provide nicotinamide/placebo powder to the child by mixing in a small volume of age-appropriate food. Because the child’s only exposure to the nicotinamide intervention during the first 6 months was through mother’s breast milk, all mothers received breastfeeding support by trained study and community healthcare workers that included counseling that exclusive breastfeeding during the first 6 months of life is recommended by the World Health Organization (WHO) and the Tanzanian Ministry of Health. Nicotinamide doses were distributed every 2 months, and pill count or sachet counts were performed when the next installment of doses was delivered. Mothers were asked monthly about the frequency of nicotinamide doses ingested.

Study visits were conducted in the family’s home at enrollment and monthly from age 1 through 18 months, occurring within a 7-day window around these dates. Child length, weight, mid-upper arm circumference (MUAC), and head circumference were measured every 3 months starting at enrollment. At these visits, length was assessed using measuring boards, weight was assessed using digital scales, and MUAC and head circumference were assessed using measuring tape, with additional details described previously [18]. For quality control, study administrative personnel conducted random anthropometry assessments on a random subset of participants to compare to field worker measurements, revealing Pearson r correlations of 0.96 for length, 0.98 for weight, 0.97 for head circumference, and 0.89 for MUAC measures.

At all monthly visits, mothers provided responses to questionnaires regarding adherence to intervention medications, breastfeeding practices, child illness symptoms, child healthcare use, and all medication use. At the baseline visit, the mother provided information on demographic and economic factors, including tribal affiliation and the ward (geographical area) in which the family lived. Participant socioeconomic status (SES) was assessed using a scoring system developed from the Etiology, Risk Factors, and Interactions of Enteric Infections and Malnutrition and the Consequences for Child Health (MAL-ED) study based on improved water and sanitation, assets, maternal education, and household income (WAMI) [20].

Between May 3, 2019 and July 8, 2019 the study was suspended by Tanzanian regulatory agencies over procedural concerns including the timing of laboratory testing and the total number of serious adverse events (SAEs) that were then resolved (see S1 Text). This suspension occurred after recruitment was complete and did not affect participant numbers. During this time, participants were allowed to complete the study medication that they have been given (e.g., nicotinamide, which was distributed as a 2-month supply) and continued to be followed for adverse events (AEs). Participants who had completed the study during the suspension received their final study visit immediately after the study was restarted.

### Outcomes

The primary outcome was mean LAZ at 18 months, and secondary outcomes were 18-month weight-for-age z-score (WAZ), head circumference-for-age z-score (HCZ), mid-upper arm circumference-for-age z-score (MAZ), all-cause mortality, hospitalization, and childhood illness (diarrhea, lower respiratory infection, or febrile illness). Anthropometric z-scores were calculated using WHO Growth Standards and then cleaned as described in the statistical analysis plan (S1 Analysis Plan).

As part of monitoring for AEs, we performed phlebotomy on a subset of children at months 2, 8, and 18 to assess for differences between nicotinamide treatment groups in elevations in hepatic, chemistry, and hematology studies. Hepatic and chemistry analyzes were performed using a Roche Cobas 400 Plus (Roche Holding, Basel, Switzerland), and hematology assessments were performed on an Abbott Cell Dyne Ruby (Abbott Laboratories, Chicago, USA). Appropriate quality control measures were followed for all studies.

Antimicrobial resistance testing was performed according to guidelines of the Clinical and Laboratory Standards Institute as described previously [21]. Briefly, *E*. *coli* was cultured from fresh stool samples, confirmed with indole testing, and archived in pools of 5 colonies; these pools contained a mixture of *E*. *coli* phenotypes if more than one was present. Pooled *E*. *coli* underwent testing for azithromycin resistance, along with 18 additional antibiotics. Swabs inoculated with pooled *E*. *coli* isolates were streaked on MacConkey agar and incubated at 37°C for 2 to 6 hours. Mueller–Hinton agar was inoculated, and antimicrobial discs were placed on the plates, which were then inoculated at 37°C for 16 to 18 hours. For quality control, we assessed the appropriate American Type Culture Collection strains once weekly. Azithromycin minimum inhibitory concentration (MIC) was determined by Etest. The Clinical and Laboratory Standards Institute cutoffs for azithromycin resistance have not been established for *E*. *coli*; we used an MIC cutoff of ≥32 as used previously [21].

### Statistical analysis

For the assessment of growth outcomes at 18 months, we used the modified intention-to-treat group, which included all randomized participants with a valid 18-month measurement. We first examined whether there was a significant interaction effect between the nicotinamide and antimicrobial interventions on the primary and secondary anthropometry outcomes (*p* for interaction <0.05). When no significant effect was present, we estimated the effect of each intervention separately between each of the main interventions groups, those receiving nicotinamide versus those receiving placebo, and those receiving antimicrobial versus those receiving placebo. We used *t* tests to analyze the difference in means of anthropometry outcomes. A priori, we were powered to detect as little as a 0.176 difference in LAZ at 18 months for testing the main effect for each of the 2 interventions at the 5% level, with 80% power taking into account a 10% dropout rate. As part of the prespecified statistical analysis plan, for our adjusted analysis for each anthropometry outcome, we included baseline measure plus any of the prespecified covariates that were found in univariable linear regression analyses (with the covariate as predictor and anthropometry measure as the outcome) to have *p* < 0.2 for the association or have an effect size of >0.2 z-scores for that anthropometry measure. The adjusted analysis included all covariates, thus identified (plus baseline values of that anthropometry measure) in a multivariable model assessing the effect of the intervention (nicotinamide or antimicrobial) on the anthropometry outcome of interest. For baseline characteristics with less than 5% missing data, we single imputed the mean values to retain all children in the adjusted analysis and retain comparability with the unadjusted analysis. No characteristics had greater than 5% missing data. We conducted unadjusted and adjusted analyses using the same procedure for a prespecified sensitivity analysis that excluded children who had the 18-month anthropometry measurement taken outside of the designated time window and for a prespecified per protocol analysis. The per-protocol group was all children with any breastfeeding through age 6 months, received all doses of azithromycin and nitazoxanide with no more than one of these outside the 14-day window around the target date, and received at least 50% of nicotinamide doses by pill and sachet counting. We also included the following sensitivity analyses that were not prespecified: (1) we performed an analysis that included all of the potential confounders regardless of their association with the outcome; (2) we assessed the effects of the intervention on subgroups; and (3) to analyze for any potential survivor bias (due to death or loss to follow-up), we performed an analysis weighted to account for probability of censoring for each participant.

We estimated incidence rate ratios for hospitalizations and childhood illnesses between each of the main interventions and the corresponding placebo groups using negative binomial regression. Adjusted models included covariates that had *p* < 0.2 from a univariable negative binomial regression model between the outcome of interest and each covariate. We estimated unadjusted effects on mortality with log-rank tests. We estimated adjusted effects on mortality using Cox proportional hazards models.

We conducted post hoc analyses of the mean difference between each intervention on 18-month LAZ using the same methods above in the following subgroups: infant sex, WAMI quartile, birth season, and enrollment weight.

The study was registered in ClinicalTrials.gov (NCT03268902) prior to the start of enrollment, and a statistical analysis plan was added prior to the completion of data collection, cleaning, analysis, and unblinding (see S1 Analysis Plan).

## Results

**Fig 1:**
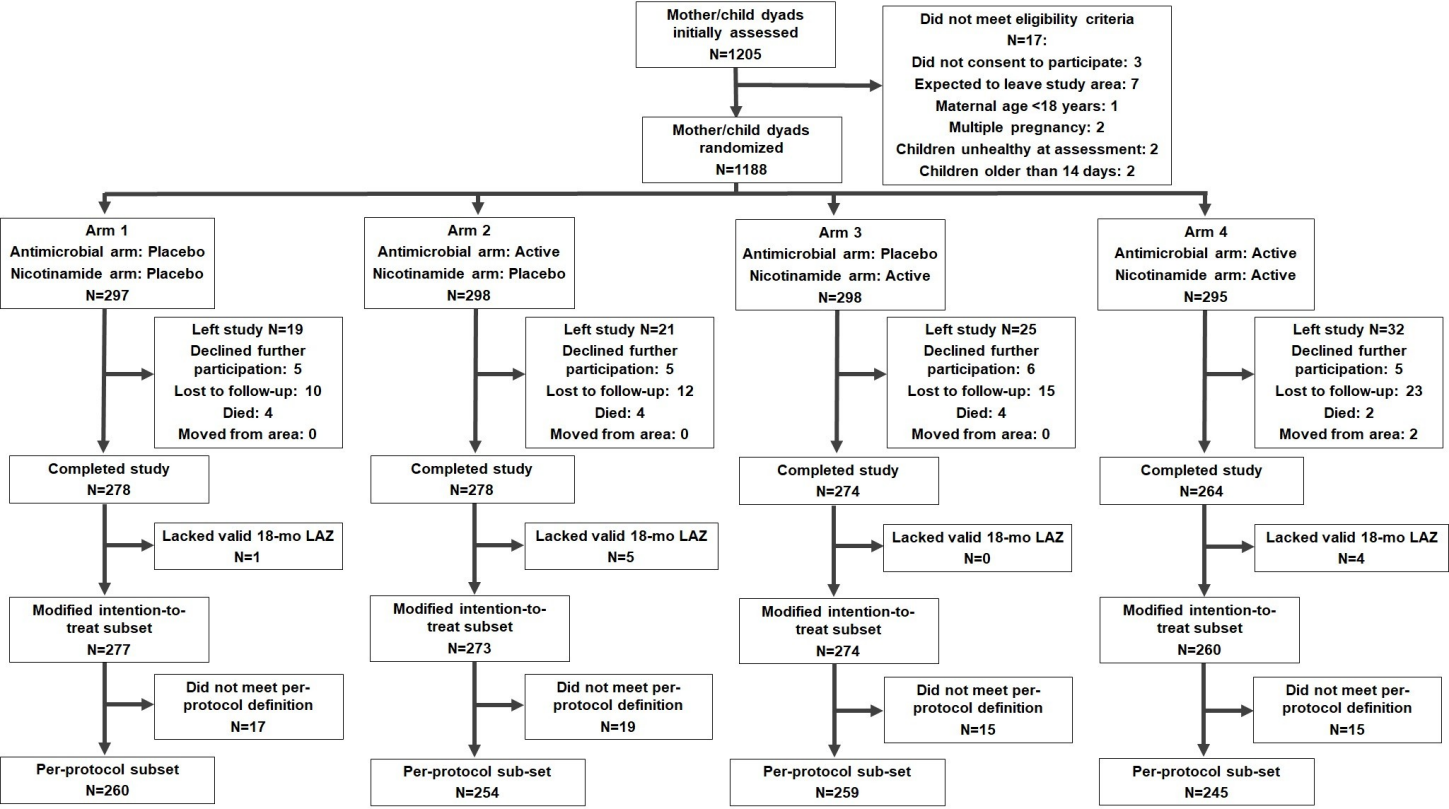
Trial profile by intervention subgroup.

Between September 5, 2017 and August 31, 2018, we identified 1,205 mother–child dyads, of whom 17 did not meet criteria (the most common reason for which was intention to relocate away from the study region during the trial); we thus enrolled 1,188 mother–child dyads (Fig 1). Of the remaining 1,188 participants, 295 were randomized to receive the active form of both interventions, 298 received only antimicrobials, 298 received only nicotinamide, and 297 received the placebos for both interventions (S2 Fig, S1 Table). From this group, 97 participants left the trial, and an additional 7 lacked a valid LAZ assessment at 18 months, leaving 1,084 participants in the modified intention-to-treat group. An additional 66 participants did not meet the per-protocol definition, leaving 1,018 participants in the per-protocol group.

Baseline characteristics of the overall cohort have been reported previously [17], and baseline characteristics of the modified intention-to-treat group are shown in Table 1. As expected from randomization, baseline characteristics were similar between arms, with the exception of enrollment head circumference z-scores, which were higher in the nicotinamide group compared to placebo (0.03 versus −0.09, difference 0.12, 95% confidence interval [CI] −0.249, −0.01) (Table 1).

**Table 1:** Baseline characteristics of the full recruited cohort by intervention arm*.

	Arm 1 (*n* = 297)	Arm 2 (*n* = 298)	Arm 3 (*n* = 298)	Arm 4 (*n* = 295)
**Intervention assignment**				
Antimicrobial arm	Placebo	Active	Placebo	Active
Nicotinamide arm	Placebo	Placebo	Active	Active
*Sociodemographics*				
Female sex	140 (47.1%)	144 (48.3%)	145 (48.7%)	148 (50.2%)
Hospital birth	167 (56.2%)	162 (54.4%)	145 (48.7%)	146 (49.5%)
Maternal age	27.71 ± 7.11	27.67 ± 6.64	27.68 ± 6.07	27.39 ± 6.52
Maternal height (cm)	157.7 ± 5.36	157.21 ± 5.32	157.41 ± 5.55	156.97 ± 5.8
Mother with ≥7 years of education	236 (79.5%)	213 (71.5%)	218 (73.2%)	215 (72.9%)
Monthly income (/1,000TSH)	50.5 ± 65.2	49.2 ± 47.7	48.3 ± 47.6	47.6 ± 43.2
**Risk factors**				
Access to an improved drinking water source	192 (64.6%)	197 (66.1%)	199 (66.8%)	189 (64.1%)
Drinking water >10 minutes from home	242 (81.5%)	239 (80.2%)	243 (81.5%)	230 (78%)
Access to an improved latrine	40 (13.5%)	26 (8.7%)	28 (9.4%)	34 (11.5%)
Crowding (>2 persons per room)				
Agricultural land ownership	282 (94.9%)	287 (96.3%)	287 (96.3%)	283 (95.9%)
WAMI index score (median, IQR)**	0.3 (0.1)	0.3 (0.1)	0.3 (0.1)	0.3 (0.1)
**Anthropometry**				
Enrollment length in cm (SD) z-score (SD)	49.22 (2.05)–0.73 (0.98)	48.82 (2.12)–0.90 (1.05)	49.1 (2.06)–0.77 (1.05)	49.06 (2.08)–0.78 (0.97)
Enrollment weight in kg (SD) z-score (SD)	3.14 (0.48)–0.59 (0.97)	3.12 (0.45)–0.60 (0.91)	3.12 (0.47)–0.62 (1.01)	3.13 (0.51)–0.61 (1.03)
Enrollment head circumference in cm (SD) z-score (SD)	34.71 (1.35)–0.06 (1.02)	34.58 (1.35)–0.13 (1.03)	34.82 (1.36)–0.07 (0.99)	34.76 (1.31)–0.01 (0.99)

* Mean ± SD is shown for continuous variables and number (percentage) for dichotomous variables unless otherwise stated.

** See reference [20].

IQR, interquartile range; SD, standard deviation; TSH, Tanzania shillings; WAMI, water and sanitation, assets, maternal education, and household income.

Participants in the antimicrobial arm received 86% of azithromycin doses and 91% of their nitazoxanide courses. By pill/sachet count, 64.1% of participants received at least 80% of their nicotinamide or placebo, and 97.2% received at least 50% of their doses (S2 Table). Caregivers reported large numbers of additional antimicrobial courses (i.e., either from local healthcare providers or administered by the family without a prescription). This totaled 2,729 courses among 918 participants, 6.99 per person year), including 1,589 courses of beta lactams, 214 of macrolides, and 250 of sulphonamides (S2 Table).

Breastfeeding was common through 6 months (while mothers were taking the nicotinamide/placebo pills), with 52% of women reporting exclusive breastfeeding, an additional 45% reporting predominant breastfeeding, and the remaining 2.5% reporting some breastfeeding that was less than predominant (S3 Table). Both mothers and children received the majority of doses of nicotinamide, with 76% of mothers receiving at least 80% of doses (by pill counting) and 64.1% of children receiving at least 80% of doses; adherence did not differ between active and placebo groups (S3 Table).

### Primary outcome

Mean LAZ at 18 months was not different between those who received antimicrobials versus placebo (−2.05 versus −2.05, mean difference: 0.007, 95% CI: −0.126, 0.112; *p* = 0.91) (Table 2). Similarly, mean LAZ at 18 months was not different between those who received nicotinamide versus placebo (−2.06 versus −2.04, mean difference: 0.027, 95% CI: −0.146, 0.092; *p* = 0.66).

**Table 2:** The 18-month anthropometry outcomes from unadjusted and adjusted analyses for the modified intention-to-treat analysis*.

	Nicotinamide					
Variable	Placebo	Active	Difference in z-scores, unadjusted (CI)	p-value	Difference in z-scores, adjusted	p-value
Length, z-score (SD) measurement in cm (SD) (n = 1,084)	−2.04 (0.95) 75.9 (2.79)	−2.06 (1.04) 76.0 (2.94)	0.02 (−0.15, 0.09)	0.66	0.04 (−0.06, 0.14)	0.45
Weight, z-score (SD) measurement in kg (SD) (n = 1,080)	−0.94 (0.94) 9.60 (1.10)	−0.94 (0.99) 9.60 (1.10)	0.00 (−0.11, 0.116)	0.98	0.01 (−0.01, 0.11)	0.88
Head circumference, z-score (SD) measurement in cm (SD) (n = 1,083)	−0.31 (0.98) 46.4 (1.44)	−0.20 (0.96) 46.6 (1.40)	0.11 (−0.218, 0.013)	0.08	0.05 (−0.05, 0.15)	0.31
MUAC, z-score (SD) measurement in cm (SD) (n = 1,078)	0.17 (0.89) 14.9 (1.07)	0.15 (0.94) 14.9 (1.13)	−0.02 (−0.09, 0.13)	0.69	−0.02 (−0.13, 0.09)	0.73
	**Antimicrobial**					
**Variable**	**Placebo**	**Active**	**Difference in z-scores, unadjusted**	**p-value**	**Difference in z-scores, adjusted**	**p-value**
Length, z-score (SD) measurement in cm (SD) (n = 1,084)	−2.05 (1.01) 76.0 (2.89)	−2.05 (0.99) 75.9 (2.83)	0.00 (−0.13, 0.11)	0.91	0.07 (−0.03, 0.17)	0.18
Weight, z-score (SD) measurement in cm (SD) (n = 1,080)	−0.93 (0.95) 9.61 (1.08)	−0.94 (0.99) 9.59 (1.09)	0.01 (−0.11, 0.13)	0.86	0.01 (−0.09, 0.12)	0.84
Head circumference, z-score (SD) measurement in cm (SD) (n = 1,083)	−0.23 (0.98) 46.5 (1.47)	−0.28 (0.96) 46.5 (1.38)	0.05 (−0.08, 0.16)	0.49	0.02 (−0.08, 0.12)	0.70
MUAC, z-score (SD) measurement in cm (SD) (n = 1,078)	0.14 (0.91 14.9 (1.09)	0.19 (0.93) 14.9 (1.11)	0.05 (−0.16, 0.06)	0.37	0.07 (−0.04, 0.18)	0.20

* Adjustments based on baseline measures and these individual covariates being associated with final outcome: LAZ: baseline LAZ, age in days at 18-month measurement, ward, hospital birth, birth month, years of maternal education, SES quartile category, sex, whether the mother was a member of the Datoga tribe, and mother’s height; WAZ: baseline WAZ, age in days at 18-month measurement, firstborn, ward, hospital birth, birth month, years of maternal education, SES quartile category, sex, whether mother was a member of the Datoga tribe, and mother’s height and weight; HCZ: baseline HCZ, ward, hospital birth, birth month, SES quartile category, and mother’s height, weight, and age; and MUAC-Z: baseline WAZ (baseline MUAC not available for most participants), age at 18-month measurement, firstborn, ward, hospital birth, birth month, years of maternal education, SES quartile category, sex, and mother’s height and weight.

CI, confidence interval; HCZ, head circumference-for-age z-score; LAZ, length-for-age z-score; MUAC, mid-upper arm circumference; SES, socioeconomic status; SD, standard deviation; WAZ, weight-for-age z-score.

There was no significant interaction between intervention with antimicrobials and nicotinamide on either the primary or any of the secondary outcomes (S4 Table).

There remained no difference in LAZ between either antimicrobial or nicotinamide intervention groups compared to corresponding placebo groups following adjustment for possible confounders from the prespecified analysis (Table 2; analysis to assess for confounders, S5 Table). Potential confounders evaluated for in the prespecified analysis were sex, WAMI score, maternal age, maternal height, maternal tribe (Tatoga versus not), maternal education ≥7 years versus <7 years), birth month, hospital birth (versus not), firstborn status, ward of residence, and child age when 18-month outcome was assessed. Individual confounders included in the analysis for each anthropometry outcome are provided in Table 2. Sensitivity analyses assessing for all possible confounders provided similar results (S6 Table).

There were also no effects of antimicrobials or nicotinamide on mean LAZ at 18 months in subgroups defined by sex, SES quartile, birth season, or low versus high entry weight (S3 Fig).

### Weight, head circumference, and MUAC

There was no difference in secondary anthropometry outcomes at 18 months between intervention groups based on either antimicrobial or nicotinamide, including WAZ, HCZ, and MUAC-Z (Table 2). These values did not change appreciably following adjustment for related factors (Table 2). Similarly, there was no difference in anthropometry measures between groups in a sensitivity analysis assessing only those whose 18-month anthropometry was assessed in the 18-month window and assessing per protocol based on study medication adherence and when weighted to account for possible differential death or dropout between intervention groups (S7–S9 Tables).

### Growth trajectories

**Fig 2:**
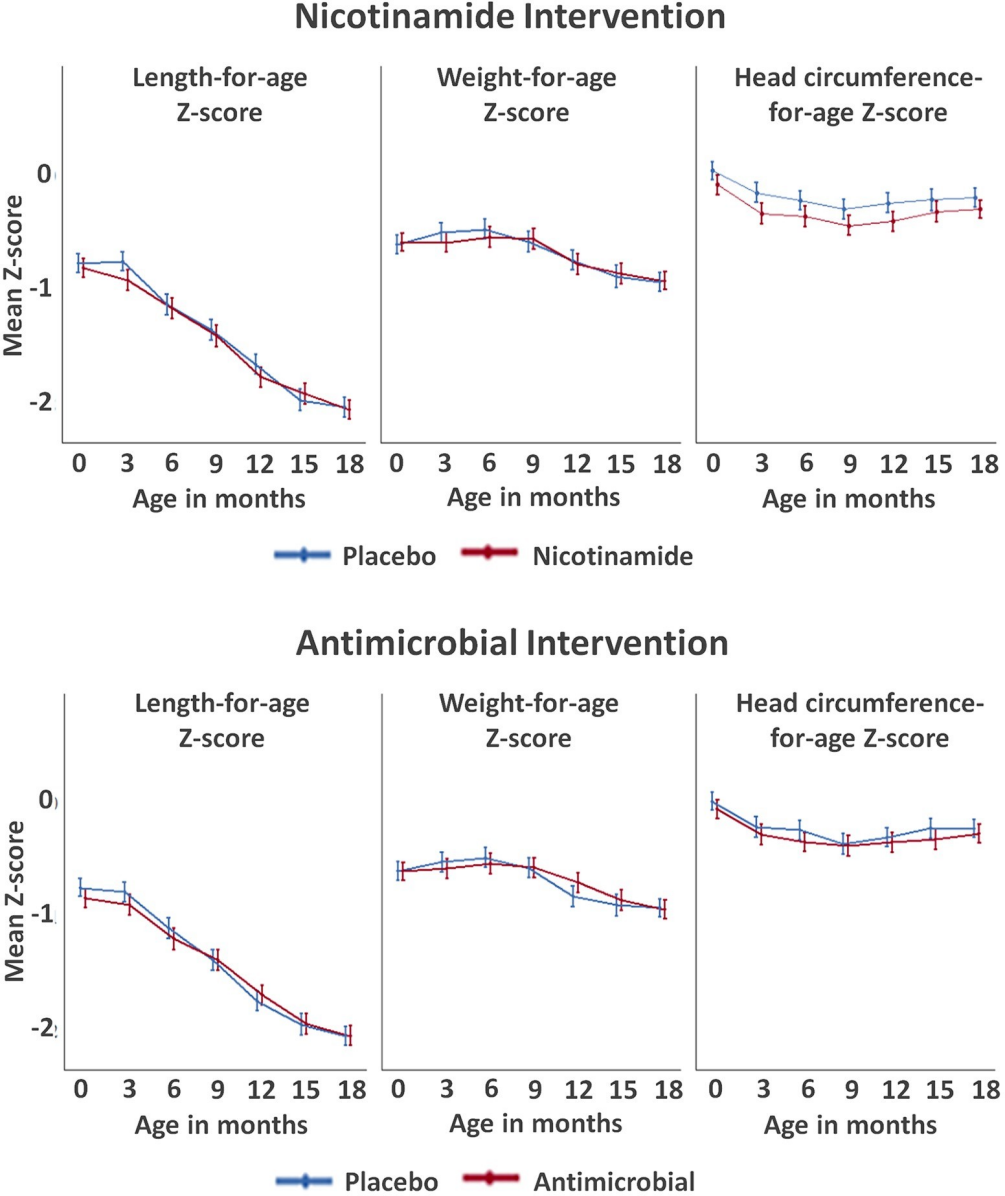
Anthropometry z-scores over time by intervention group for the modified intention-to-treat analysis (error bars represent 95% CIs). CI, confidence interval.

All groups exhibited a steady decline in mean LAZ scores as the children aged (Fig 2), although with a wide distribution of LAZ between participants at 18 months (S4 Fig). The prevalence of stunting between antimicrobial versus placebo arms was 25.1% versus 22.7% at 6 months (i.e., before antimicrobial treatment began) and 49.9% versus 48.4% at 18 months (S10 Table). For the nicotinamide intervention, stunting prevalence between nicotinamide versus placebo arms was 12.0% versus 15.4% at 3 months and 46.6% versus 51.5% at 18 months (*p* = 0.09).

Attained weight was not as severely suppressed as was length, with a drop in WAZ only starting at age 6 to 9 months and a final prevalence of WAZ <−2 of 13.5% versus 12.9% for the antimicrobial versus placebo arms and 13.4% versus 13.2% for the nicotinamide versus placebo arms (Fig 2). Head circumference z-scores exhibited an initial decrease between birth and 9 months before slightly increasing thereafter. Results were similar when restricted to a per-protocol group based on study medication adherence (S5 Fig).

### Surveillance laboratory assessment to assess safety

In laboratory assessments performed in a subset of children at months 2, 8, and 18 to follow the safety of the nicotinamide intervention, the only differences between groups was that at 8 months, children receiving nicotinamide had higher hemoglobin (11.3 [11.0, 11.5] versus 10.8 [10.5, 11.1], *p* = 0.03) and hematocrit (33.4 [32.6, 34.1] versus 31.8 [30.9, 32.7], *p* = 0.01) (S11 Table).

### Antimicrobial resistance testing

Antimicrobial resistance was tested on fecal *E*. *coli* from a random subset of participants at 18 months from 92 participants, 56 in the treatment arm and 36 in the placebo arm. *E*. *coli* was isolated from 89 of these participants, with a total of 1,399 cultures, from which 8 had no *E*. *coli* growth. Testing revealed that 32% of those in the antimicrobial group and 23% of those in the placebo group exhibited resistance to azithromycin at the end of the study (*p* = 0.352).

### SAEs

There was no difference in overall AEs between intervention groups, either between the nicotinamide versus placebo groups over the full 18 months or between the antimicrobial versus placebo groups after age 6 months (when the first antimicrobial dose was given), and none of these AEs were attributed to the interventions (Table 3). In assessing individual categories of AEs, there was a lower amount of reported symptoms of acute lower respiratory infection (ALRI, including pneumonia and bronchiolitis in this age range) in the antimicrobial versus placebo group (583 events among 595 participants versus 500 events in 593 participants, *p* = 0.03; this difference persisted after adjustment for potential confounders). There were similar numbers of SAEs in the nicotinamide and placebo groups (145 versus 147); there were also similar numbers of SAEs in the antimicrobial and placebo groups (90 versus 86). Overall, there were 14 deaths, 8 in the placebo group and 6 in the nicotinamide group. There were 3 deaths after 6 months—2 in the placebo group and 1 in the antimicrobial group. There was no difference in time to first SAE between the nicotinamide versus placebo or antimicrobial versus placebo (S6 Fig).

**Table 3:** AEs and SAEs by intervention group (beginning at time of first nicotinamide dose at enrollment and first antimicrobial dose at 6 months).

			Unadjusted analysis		Adjusted analysis**	
	Placebo	Nicotinamide	IRR (CI), Nicotinamide versus placebo	*p*-value	IRR (CI), Nicotinamide versus placebo	*p*-value
All events from enrollment*						
All AEs	2,289	2,318	1.04 (0.97, 1.11)	0.31	1.05 (0.98, 1.12)	0.21
ALRI	1,008	999	1.02 (0.92, 1.12)	0.74	1.01 (0.92, 1.12)	0.81
Diarrhea	683	700	1.05 (0.93, 1.18)	0.43	1.07 (0.96, 1.21)	0.23
Fever	83	80	0.99 (0.72, 1.35)	0.94	1.00 (0.73, 0.14)	0.99
*SAEs*						
All SAEs	145	147	1.04 (0.801, 1.354)	0.76	1.00 (0.77, 1.30)	0.99
ALRI	78	68	0.89 (0.626, 1.286)	0.56	0.87 (0.61, 1.25)	0.47
Diarrhea	55	57	1.05 (0.697, 1.592)	0.81	1.01 (0.66, 1.52)	0.98
	Placebo	Antimicrobials	IRR (CI), Antimicrobials versus placebo		IRR (CI), Antimicrobials versus placebo	
All events after age 6 months*						
All AEs	1,544	1,439	0.94 (0.87, 1.02)	0.16	0.94 (0.87, 1.02)	0.14
ALRI	583	500	0.87 (0.76, 0.99)	0.03	0.88 (0.773, 1.00)	0.05
Diarrhea	529	488	0.93 (0.81, 1.06)	0.26	0.92 (0.81, 1.05)	0.23
Fever	70	56	0.81 (0.57, 1.16)	0.25	0.81 (0.57, 1.16)	0.25
*SAEs after 6 months*						
All SAEs	90	86	0.96 (0.69, 1.34)	0.83	0.96 (0.69, 1.32)	0.79
ALRI	33	33	1.01 (0.60, 1.69)	0.97	0.98 (0.58, 1.66)	0.94
Diarrhea	45	45	1.01 (0.64, 1.60)	0.97	1.04 (0.65, 1.66)	0.87

* This includes SAE and non-SAEs.

** Adjusted for all factors (from S1 Table) independently associated with outcome: All AEs: ward, WAMI, birth month, sex, born in hospital, and mother’s weight; All ALRI: ward, WAMI, birth month, firstborn, born in hospital, and Datoga; All Diarrhea: ward, WAMI, birth month, sex, born in hospital, Datoga, mother’s weight, and mother’s school years; SAEs: ward, WAMI, and birth month; SAE ALRIs: ward, WAMI, season, mother school years, and firstborn; and SAE Diarrhea: ward and maternal education >7 years.

AE, adverse event; ALRI, acute lower respiratory infection; CI, confidence interval; IRR, incidence rate ratio; SAE, serious adverse event; WAMI, water and sanitation, assets, maternal education, and household income.

## Discussion

This interventional trial, conducted in a region of rural sub-Saharan Africa with a heavily maize-based diet and where children by age 18 months have a high prevalence of stunting and intestinal pathogen carriage, did not find that administration of daily nicotinamide, or scheduled antimicrobials, improved linear growth. Among those receiving active treatment or placebo for each of these interventions, we found a high prevalence of stunting—nearly 50%. For the antimicrobial intervention, this raises the potential that either this intervention did not adequately reduce pathogen carriage or that prior links noted between enteric pathogen burden and poor growth may have represented association and not causation [7], while for the nicotinamide intervention, this suggests against a growth benefit of augmenting the niacin pathway, even in an area with a maize-based diet. Overall, these data may serve best to emphasize the myriad health challenges in resource-poor areas, where ongoing nutritional deficiencies and other disease factors predominate in causing the growth suppression observed [1].

Pathogen carriage contributes to enteric and systemic inflammation, which is associated with disruption of growth factors [22,23] and direct effects at the growth plates [24]. However, even in the presence of causal effects of pathogens on growth, there are multiple potential reasons why antimicrobial administration may not affect linear growth: a low prevalence of pathogens targeted, resistance of organisms to the agents used, and an inadequate dose and timing of the antimicrobials; we will address each of these considerations briefly below. With respect to the pathogen prevalence in this area, we had noted in a prior study—through surveillance of monthly stools using real-time polymerase chain reaction (RT-PCR) for common enteric pathogens—that at age 18 months, there was a high prevalence of children without diarrhea who were carrying pathogens, including *Campylobacter* (50%), enteroaggregative *E*. *coli* (22%), enterotoxigenic *E*. *coli* (10%), and atypical enteropathogenic *E*. *coli* 10% (all susceptible to azithromycin), as well as *Giardia* (35%) and *Cryptosporidium* (6%) (both susceptible to nitazoxanide)—with the majority of children carrying more than 1 pathogen. While it is possible that this prevalence of carriage had decreased in the 5 years between studies, this is unlikely given the continued low socioeconomic conditions in the area, although confirmatory testing of pathogens remains needed [17].

Another important consideration is the potential for underlying resistance to antimicrobials. At age 18 months, we noted a relatively low degree of resistance to azithromycin (<33% in both arms), which was nevertheless higher than had been noted earlier in this region (6% at 18 months) [21]. This may be because the underlying use of azithromycin by local healthcare providers and families remains relatively low in the region, accounting for only 8% of external antimicrobial courses reported. Importantly, the group that received the azithromycin did not have a statistically significant difference in the degree of antimicrobial resistance at the end of the study, which was present in 32% versus 23% (*p* = 0.352). This is consistent with prior studies demonstrating that the association between azithromycin exposure and *E*. *coli* resistance is short lived [25].

The dose and frequency of antimicrobial administration likely also plays a role in potential efficacy of antimicrobial intervention. We chose to assess quarterly administration of a single dose of azithromycin (20 mg/kg—the same dose used biannually in a previous study assessing effects of scheduled antimicrobials on mortality [26]), as this approach had the potential for consideration as a public health intervention. In the prior study, this dose administered as mass drug administration every 6 months led to persistent differences in pathogen carriage after 24 months, including some *Campylobacter* species [27]. Among those receiving antimicrobials, we did note a lower odds ratio (OR) for ALRI (0.88, Table 3), suggesting potential efficacy for short-term respiratory infection—either from direct effects on pathogens or additional reported anti-inflammatory and immunomodulatory effects of azithromycin [28]. However, it remains unclear what the differences are between the single dose versus multiday course of azithromycin with respect to long-term pathogen reduction. For nitazoxanide, we provided the medication at age 12 and 15 months as a complete 3-day course, because in prior studies, this has been shown to be effective in the treatment of diarrhea from *Cryptosporidium* [10] and *Giardia* [29] in children in this age range; however, it remains possible that this intervention was too late, and the trajectory of growth-limiting effects was already well established.

Finally, there is potential that ongoing exposure to new pathogens could be frequent enough that reduced carriage was not sustained. It is likely that children in this area have multiple sources of pathogen exposure. Indeed, the WASH Benefits study in Bangladesh [30] and the Sanitation Hygiene Infant Nutrition Efficacy (SHINE) trial in Zimbabwe [31] attempted to reduce pathogen exposure by introducing latrines, increased handwashing, and clean water but found no change in child growth, along with no difference in pathogen carriage [31]. Another study demonstrated that rectal swabs obtained 14 days after acute diarrhea showed new infections, rather than persistent carriage, further suggesting limitations to periodic intervention in settings of high enteropathogen burdens [32].

We targeted supplementing the tryptophan–kynurenine–niacin pathway because of prior data associating low tryptophan with poor growth, the central importance of NAD+ metabolism in energy utilization and immune response, and the high potential for niacin/nicotinamide inadequacy in a setting where diets consist predominantly of maize [13]. In addition, promising preclinical data suggested that providing a related metabolite, nicotinamide riboside, to pregnant and nursing mice provided health benefits to the offspring, including larger size at weaning and neurodevelopmental advantages [12]. We selected doses that reflected currently available preparations (in the case of the maternal dose during lactation) and the upper end of the tolerable range for infants. Despite the lack of difference in LAZ between those receiving placebo versus nicotinamide groups (−2.06 versus −2.04), there was a nonsignificant increase in stunting prevalence among those in the placebo group (51.8% versus 46.4%, *p* = 0.09), with a spectrum of linear growth outcomes in both groups almost entirely below average; the difference in prevalence of stunting had increased from age 9 months and suggests the potential that longer-term treatment could be needed to see effects in a subgroup of children. As part of AE surveillance, we assessed ongoing laboratory studies, revealing slightly higher levels of hemoglobin and hematocrit among nicotinamide versus placebo treated children at 8 months. These higher levels of hemoglobin differed from prior treatment in healthy adults, where short-term (9 days) treatment with the derivative nicotinamide riboside caused a decrease in hemoglobin and longer-term (4 months) treatment caused no change; in a group of adults with mitochondrial myopathy and low NAD+ levels, niacin treatment resulted in a decrease in hemoglobin of unknown mechanism. Therefore, the significance of higher hemoglobin levels among infants in this setting is this setting is unclear. There were no noted detrimental differences with nicotinamide treatment, overall supporting the safety of the doses delivered. The failure of nicotinamide provision to produce differences in growth in our study suggests against a high degree of niacin/nicotinamide deficiency among children in the area or that nicotinamide does not play a strong role in improving growth in regions with widespread stunting.

Overall, the lack of benefit from either of these interventions may serve best to highlight the significant growth challenges in this setting, potentially due to socioeconomic challenges. These challenges include insufficient macronutrient and micronutrient deficiencies and inadequate energy intake, the ubiquity of pathogen exposure causing frequent infections and inflammation, and suboptimal healthcare delivery. These negative factors may predominate, limiting the ability of targeted interventions to improve growth—and emphasizing that a more global package of interventions is needed, potentially providing macronutrient supplementation such as ready-to-eat therapeutic food in addition to improved hygiene and targeted antimicrobial approaches. We did not see a difference in AEs overall between intervention groups, with a large proportion of participants in both intervention groups experiencing some AE, likely due to the underlying difficulties of life in this rural, developing area. Interestingly, we noted a slight reduction in the AEs for respiratory symptoms among those receiving azithromycin, suggesting some reduction of bacterial causes of these symptoms, although, clearly, this reduction did not result in overall improved growth.

This study benefited from a large study population, powered to detect as little as a 0.176 difference in LAZ at 18 months (approximately 0.4 cm at 18 months). In addition, families were visited in their homes monthly, providing frequently collected surveillance data. Limitations of the study include that participants self-reported much of the relevant information, including illness symptoms and use of other antimicrobials provided during ongoing care. For the nitazoxanide course, the study team was present to witness only the first dose of the 3-day course, meaning that participants may have omitted further adherence; however, all other objective measures suggested high adherence to the study interventions. While we currently lack data regarding enteral pathogen burden and additional laboratory data in these children, such data will be important to assess whether the current interventions altered pathogen carriage in the absence of effects on growth. Also, while we acquired all of the active medications (and placebos) from approved and reputable companies, we did not independently perform follow-up testing of these interventions to confirm presence (and absence) of the active agent. In conclusion, this large, single-site RCT assessing administration of scheduled antimicrobials and daily nicotinamide did not find any differences in the degree of stunting among infants where stunting is highly prevalent. While enteric pathogen carriage has been linked to poorer growth, this study showed that periodic antimicrobial intervention is insufficient to improve LAZ, even in a region with a high prevalence of enteric disease. Also, whereas deficiencies in the tryptophan–kynurenine–niacin pathway have been linked to short stature in this area, provision of nicotinamide did not prevent this. Further research remains critical for identifying interventions to improve early childhood growth and well-being in low-resource settings.

## Supporting information

S1 CONSORT Checklist Checklist of information to include when reporting a randomized trial. (DOCX)

S1 Protocol ELICIT study protocol. ELICIT, Early Life Interventions for Childhood Growth and Development in Tanzania.
(DOCX)

S1 Text Resolution of study suspension by NIMR, Tanzania. NIMR, National Institute for Medical Research.
(PDF)

S1 Analysis Plan Statistical analysis plan posted on ClinicalTrials.gov prior to study completion. (DOCX)

S1 Fig Factorial design. (DOCX)

S2 Fig Trial profile by individual intervention. (DOCX)

S3 Fig Subgroup analysis for effect of nicotinamide intervention **(A)** and antimicrobial intervention **(B)** on LAZ by SES (WAMI), weight at study entry, sex, and harvest season. LAZ, length-for-age z-score; SES, socioeconomic status; WAMI, improved water and sanitation, assets, maternal education, and household income.
(DOCX)

S4 Fig Distribution of anthropometry z-scores at 18 months by intervention group for the modified intention-to-treat analysis. (DOCX)

S5 Fig Anthropometry z-scores over time by intervention group for the modified per-protocol analysis. Shown are mean values with error bars representing 95% CIs. CI, confidence interval.
(DOCX)

S6 Fig Time to SAE after enrollment by nicotinamide intervention arm **(A)** and antimicrobial intervention arm **(B)**. SAE, serious adverse event.
(DOCX)

S1 Table Baseline characteristics of the modified intention-to-treat group. (DOCX)

S2 Table Antimicrobial doses as part of intervention and other sources. (DOCX)

S3 Table Breastfeeding and nicotinamide frequency. (DOCX)

S4 Table Assessment for interaction between interventions on 18-month anthropometry outcomes. (DOCX)

S5 Table Assessment for factors associated with 18-month outcomes to determine covariates for adjusted analysis. (DOCX)

S6 Table Assessment of 18-month LAZ adjusted for all covariates listed in S5 Table (regardless of their relationship to LAZ from the prespecified selection analysis). LAZ, length-for-age z-score. (DOCX)

S7 Table Sensitivity analysis of 18-month anthropometry outcomes for those whose 18-month anthropometry was measured in the 18-month window. (DOCX)

S8 Table The 18-month anthropometry outcomes from per-protocol analysis. (DOCX)

S9 Table Primary outcome weighted to account for potential differential death or loss to follow-up by intervention group. (DOCX)

S10 Table Stunting prevalence by time point. (DOCX)

S11 Table Safety laboratory study information. (DOCX)

## Abbreviations

AE: adverse event
ALRI: acute lower respiratory infection
CI: confidence interval
CONSORT: Consolidated Standards of Reporting Trials
ELICIT: Early Life Interventions for Childhood Growth and Development in Tanzania
HCZ: head circumference-for-age z-score
LAZ: length-for-age z-score
MAL-ED: Etiology, Risk Factors, and Interactions of Enteric Infections and Malnutrition and the Consequences for Child Health
MAZ: mid-upper arm circumference-for-age z-score
MIC: minimum inhibitory concentration
MUAC: mid-upper arm circumference
NAD+: nicotinamide adenine dinucleotide
NIMR: National Institute for Medical Research
OR: odds ratio
RCT: randomized controlled trial
RT-PCR: real-time polymerase chain reaction
SAE: serious adverse event
SES: socioeconomic status
SHINE: Sanitation Hygiene Infant Nutrition Efficacy
TFDA: Tanzanian Food and Drug Administration
WAMI: water and sanitation, assets, maternal education, and household income
WAZ: weight-for-age z-score
WHO: World Health Organization
