# Supplementary material for: Effect of scheduled antimicrobial and nicotinamide treatment on linear growth in children in rural Tanzania: A factorial randomized, double-blind, placebo-controlled trial
Source: PLoS Med. 2021 Sep 28;18(9):e1003617. doi: 10.1371/journal.pmed.1003617 (PMC8478246; doi:10.1371/journal.pmed.1003617)
Supplement: S1 Protocol — ELICIT, Early Life Interventions for Childhood Growth and Development in Tanzania. (DOCX) [file pmed.1003617.s002.docx]

**
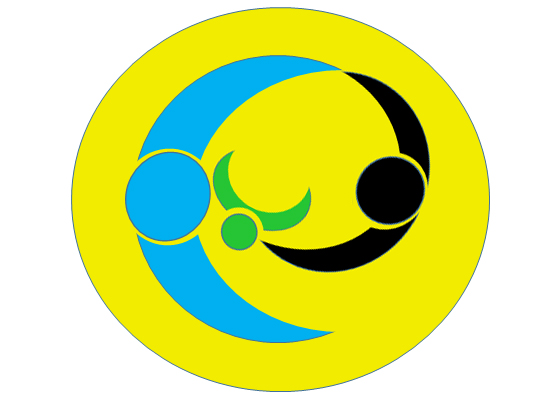
ELICIT: Early Life Interventions for Childhood Growth and Development in Tanzania**

**
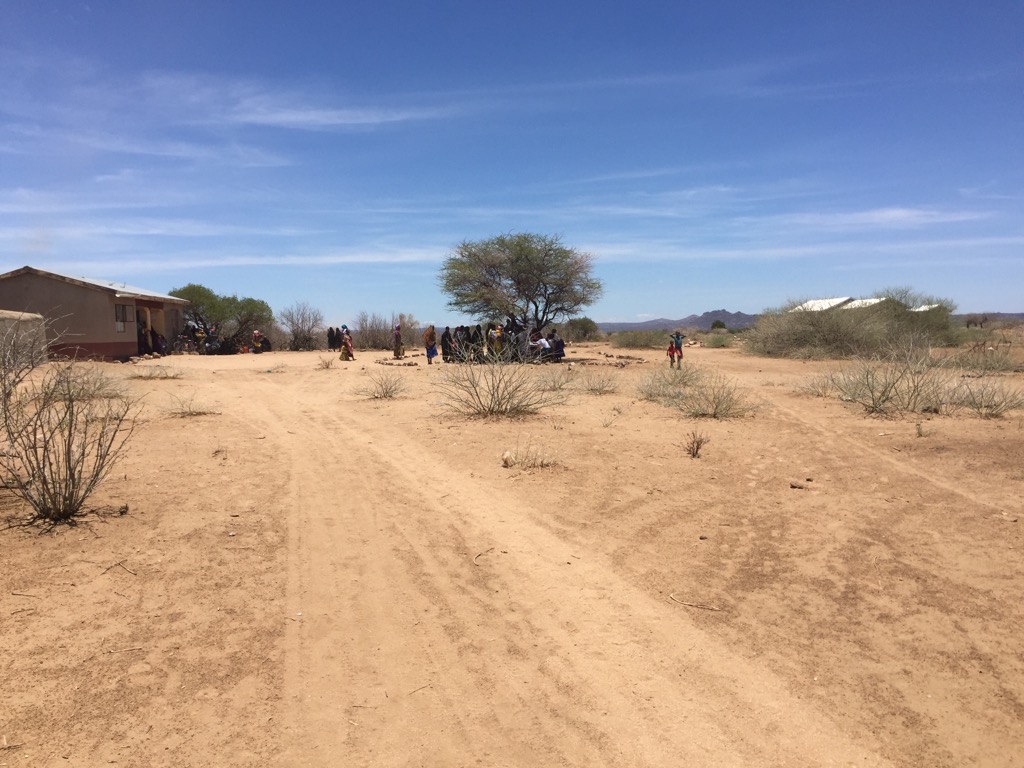
**

**Haydom Global Health Research Centre at**

**Haydom Lutheran Hospital, Haydom NIMR station**

**and**

**The University of Virginia**

Protocol Number: 19465 (UVa IRB-HSR)

Protocol Version: 6.0

Protocol Date: 07 December 2018

Sponsor name: Haydom Lutheran Hospital

Signature: _________________________________

PI name: Estomih Mduma

Signature: __________________________________

**Table of Contents** 18

[A. PROJECT SUMMARY…………………………………………………………..19](#_Toc223515680)

[I. Title:](#_Toc223515681) 19

[II. Primary Investigators:](#_Toc223515682) 19

III. Abstract…..……………………………………………………………………….. 20

[IV. Background/Introduction………………………………………………………... 21](#_Toc223515683)

[V. Hypotheses, Aims & Objectives: 22](#_Toc223515684)

[B. STUDY DESIGN AND METHODS](#_Toc223515686) 23

[I. Study design:](#_Toc223515687) 23

[II. Setting:](#_Toc223515688) 23

[III. Subjects:](#_Toc223515689) 23

IV. Timing of Treatments and Evaluations to be performed 28

a) General timeline..............................................................................…...28

[b) Vitamin B3/nicotinamide treatment 29](#_Toc223515693)

c) Antimicrobial treatment……..…………………………………………….......29

d) [Outcome measures description 29](#_Toc223515694)

[e) Timing of outcome measures](#_Toc223515695) 29

[V. Ethical considerations: 33](#_Toc223515684)

[VI. Statistical considerations: 34](#_Toc223515684)

[C. REFERENCES](#_Toc223515705) 36

A. Project Summary

I. Title:

ELICIT: Early Life Interventions for Childhood Growth and Development In Tanzania

II. Primary Investigators:

**Haydom Lutheran Hospital**

Estomih Mduma, DLSHTM, MSc/MPH

Principal Investigator for this proposal and will oversee this research project and its implementation. Dr. Mduma is experienced and skilled in clinical trial and public health research for about 16 years and work with Haydom Global Health Research Centre, at Haydom Lutheran Hospital (HLH). The extensive experience in research include Co-PI and research manager for the MAL-ED study (the founder of this ELICIT study), also other clinical trials involved as a coordinator or co-investigator; multiple NIH/BMGF projects, including Microbicides RCT and Acyclovir RCT to prevent HIV-1 transmission in HIV/HSV co-infected discordant couples, both funded by BMGF.

Other investigators:

Pascal Mdoe MD, MMED (investigator)

Joshua Gideon MD, MMED (investigator)

William Mollam Pharmacist (Investigator)

Justine Museveni MD (Investigator)

Samson Madawabora, Clinical Psychologist (investigator)

Samwel Jatosh BSc (Research coordinator)

Ladislaus Blanss (Data Manager)

Erling Svenson (Psychologist)

Rosemary Nshama, Thomas Walingo and Caroline Kimathi (Lab scientists)

Rehema Bukhay, Nicodemus Ingii and Anita Yeconia (Research Nurses)

Godfrey Guga BA (statistician)

**NIMR Haydom Station:**

Sokoine Kivuyo MD, MPhil

# University of Virginia

Mark DeBoer Pediatric Endocrinologist, Associate Professor

Pediatric Endocrinologist working with the Center for Global Health on projects related to child growth and metabolism in the setting of enteric infections and malnutrition. Assisting with the development and plan related to the planning, training, implementation and analysis of the nutrition/growth interventions and outcomes

Other investigators

Eric Houpt, MD, Professor, infectious disease physician

Rebecca Scharf, MD, MPH Developmental Pediatrician

Jean Gratz, Lab Manager

Joann McDermid, MSc, PhD, RD Assistant Professor of global nutrition

James Platts-Mills MD, Assistant Professor

Mark Conaway PhD, biostatistician

**BST Inc. (AutoAnthropometry team in collaboration with University of Virginia)**

Gene Alexander

David Reeves

III. Abstract:

Children living in rural sub-Saharan Africa experience massive challenges to child thriving, with poor linear growth and delays in child development. In a cohort of 211 children living in the rural Haydom area of Tanzania (participating in the Interactions of Malnutrition & Enteric Infections: Consequences for Child Health and Development “MAL-ED” Study^1^), 70.6% had stunted growth at 18 months. This rate of moderate and severe stunting (length-for-age z-score [HAZ] <-2 standard deviations) was the highest of the 8 study sites in MAL-ED. Due to the intensive observational data collected, we know that this enormous deficit is likely associated with high rates of enteric infections with *Campylobacter*, *E. coli* pathotypes, *Cryptosporidium*, and *Giardia*, organisms susceptible to azithromycin and/or nitazoxanide. Infections such as these occur frequently in developing areas and are often associated with environmental enteropathy, including ongoing enteric inflammation and loss of enterocyte integrity, leading to possible bacterial translocation and poorer absorption of ingested nutrients. The consequences of these infections, enteric dysfunction and poor nutrient absorption frequently include growth stunting, learning delays, and an overall loss of human capital. Emerging evidence suggests a potential role for the tryptophan-niacin pathway (including the end-product nicotinamide, an isoform of vitamin B3) in decreasing mucosal inflammation and affecting enteral microbiota. At the Tanzania site of MAL-ED, serum levels of tryptophan were related to subsequent linear growth, further suggesting importance of the tryptophan-niacin pathway. What is not clear is whether early childhood growth and development could be improved by targeting enteric infection and the tryptophan-niacin pathway by 1) delivering antibiotics against specific bacteria and/or 2) providing vitamin B3 as nicotinamide/niacinamide.

We propose to utilize an experienced research team in rural Tanzania to assess growth and cognitive effects of treatment with azithromycin and nitazoxanide (randomized together) and/or niacin treatment for targeting the infections and inflammation of environmental enteropathy. *We hypothesize that these interventions will result in improved linear growth, decreased enteric disease burden and improved child learning ability*. Our randomized trial of these inexpensive interventions will address known problems in a resource-poor setting.

IV. Background/Introduction:

**Scientific background, rationale and relevance of this project.**

In severely under-resourced areas of the world, such as the Haydom area in Tanzania (TZ), enteric infection likely contributes to worsened health-related outcomes including growth and cognitive development. Through ongoing work at the Haydom site of MAL-ED, we have been following children in an area of severe poverty, with considerable malnutrition, and high rates of enteropathogen infection. These challenges contribute to problems in the following critical domains with long-lasting sequelae:

1. **Growth deficits:** Poor weight gain and linear growth failure in childhood are often seen as surrogates for overall health status; persistently poor growth has implications for future work potential and lower human capital.^2-4^ The high rate of stunting in Tanzanian children in the MAL-ED site (Figure 1A--worst among all MAL-ED sites) is likely related to enteric infections (details of which are described further below). In addition, serum levels of tryptophan were positively associated with subsequent linear growth,^5^ suggesting a role for the tryptophan-niacin pathway.

**
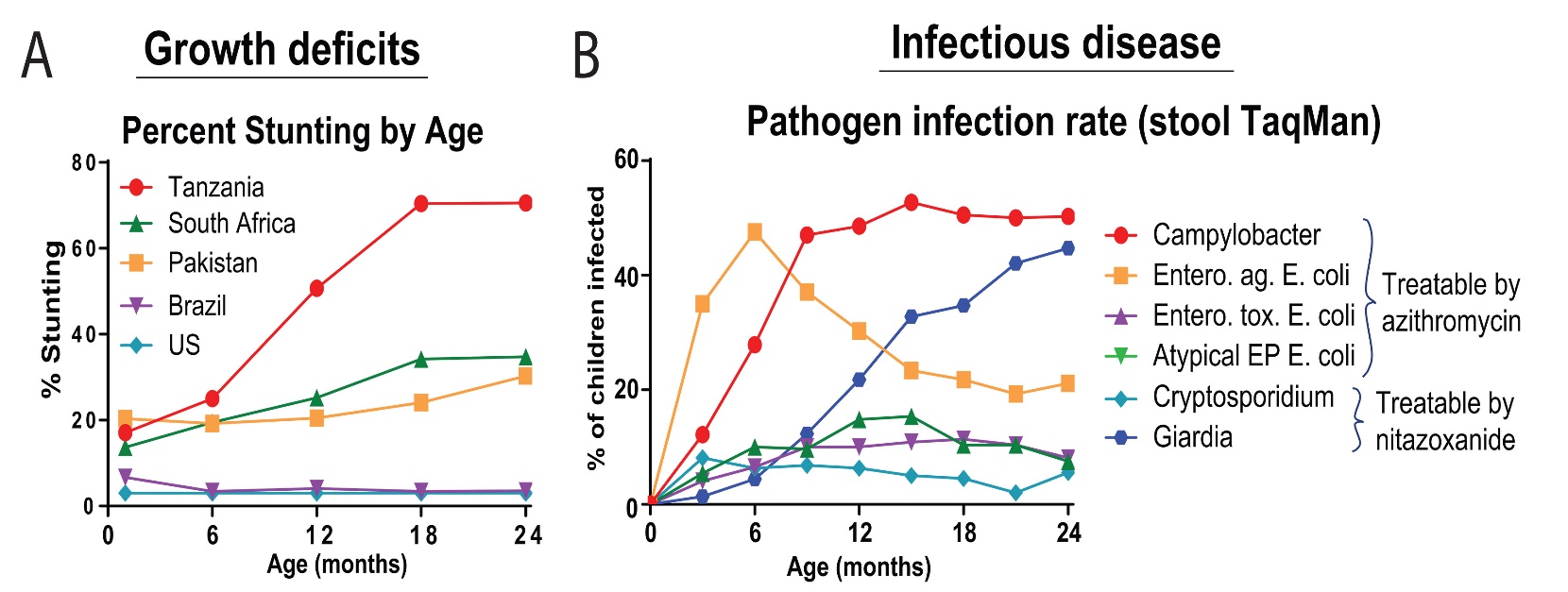
**

**Figure 1: Deficits & challenges among young children in the Haydom, TZ research site in MAL-ED.**

2. **Intestinal infections**: Preliminary data from the MAL-ED study reveals that children in TZ have a high rate of pathogen carriage and that asymptomatic infection with enteropathogens is directly associated with poor linear growth, impaired oral vaccine response, and cognitive deficits. Specifically, we found that an enteropathogen score (number of enteropathogens per stool sample) accounted for as much of a measured effect on growth as did socioeconomic status index, and those children with a high pathogen score had a measurable reduction in HAZ (-0.5 Z scores) compared to those with low pathogen burdens (Figure 2). In further support of the potential effect of enteropathogens, or pathogens in general, on childhood outcomes, single dose azithromycin given annually to an unselected group of children in Ethiopia improved survival.^6^

| 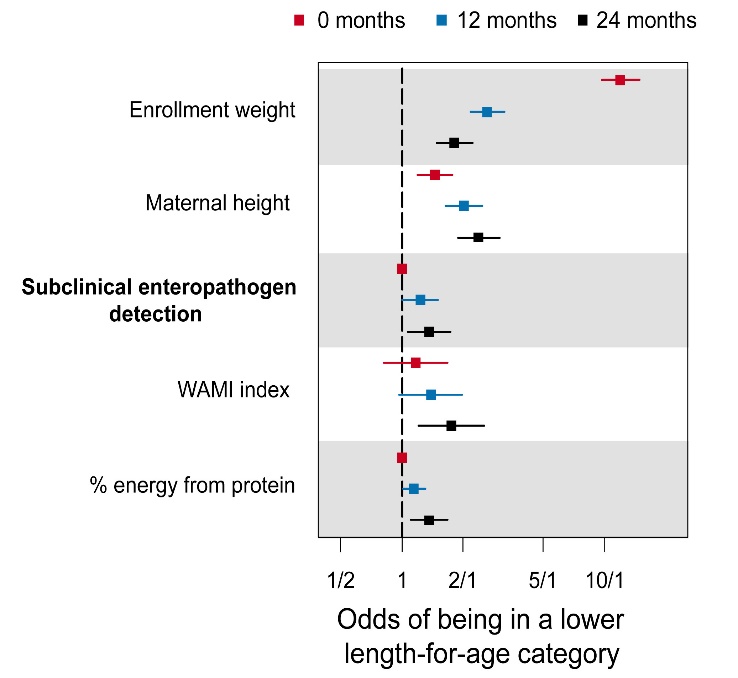 | 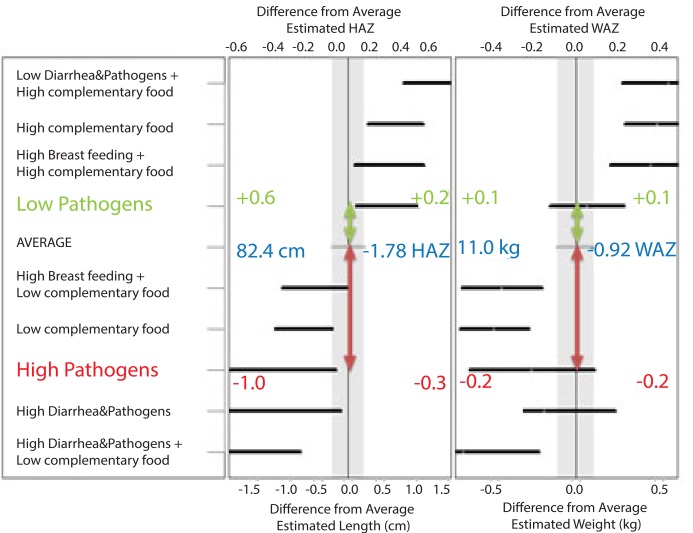 |
| --- | --- |
| **Figure 2: Preliminary data from emerging MAL-ED publications. A. Data from Checkley et al.: Subclinical enteropathogen score was a significant contributor to poor growth in MAL-ED. B. Data from Caulfield et al. showing that children with high pathogen score had lower HAZ and WAZ across 8 sites.** | |

3. **Developmental delays**: Perhaps there is no more important outcome (with the exception of mortality) than the cognitive, social and emotional development of a population, with clear extensions to human capital, economic productivity and quality of life^7-9^ in communities. In MAL-ED assessments, TZ children had fewer words than seen among US children. The reasons for potential developmental delays are multifactorial, but likely include persistent infections and malnutrition^10-15^. Studies in other developing areas have demonstrated improved cognitive development following delivery of nutrition support^16^ and treatment of infections.^17, 18^

V. Hypotheses, Aims & Objectives:

**A. Objectives**

Research questions:

1. Nicotinamide arm

Given that children in the Haydom region: a) a significant amount of stunting, b) have significant enteric pathogen carriage and c) have multiple nutrient deficiencies, AND given that abnormalities in the tryptophan/kynurenine/nicotinamide pathway have been noted in both poor growth and response to intestinal bacteria, we asked the research question: Is nicotinamide an important part of intestinal response to enteric pathogens without which children experience an increase in intestinal infections and a decrease in growth and cognitive development?

We then hypothesized that intervention with nicotinamide (vitamin B3)(compared to placebo) during early life will result in increased childhood growth and cognitive development and decreased intestinal infection and inflammation.

Primary objective: After intervention with nicotinamide (daily dose for mother during child’s age 0-6 months; daily dose for child during months 6-18), assess growth response via monthly measure, with primary outcome height-for-age z-score at age 18 months.

Secondary objectives: Assess further endpoints described in the protocol below, related to:

Intestinal pathogen burden, intestinal and systemic inflammation, and alterations in nutrition, metabolism, and hormone regulation.

• Proportion of stunting (HAZ<-2) at 18 months

• Weight-for-age (WAZ) and weight-for-height (WHZ) z-scores at 18 months

• Metabolomic assay of gut microbial metabolism and alterations to tryptophan-niacin pathway at 6, 12 and 18 months

• Tryptophan: kynurenine ratio and other tryptophan metabolites in serum at 12 and 18 months

• IGF-1 in serum at 12 and 18 months

• High-sensitivity C-reactive protein in serum at 12 and 18 months

• Stool myeloperoxidase at months 6, 12 and 18 months

• Anemia—proportion of children with moderate anemia as defined by WHO for age and altitude

• Prevalence of enteric pathogens at months 3, 6, 6+14 days, 12, 12+14 days, and 18

• Change in microbiota at months 6, 6+14 days, 12 and 18 (subset of participants)

• Symptomatic diarrhea by monthly questionnaire

• Hospitalization and all-cause mortality

• Cognitive outcomes Malawi Development Assessment Tool (MDAT) assessment, the Bayley scales of development and the Observation of Maternal and Child Interaction (OMCI) at 12-18 months, given the importance of nutrition and enteric status for the developing brain

1. Antimicrobial arm:

Given previous data from our team and others regarding the importance of enteric pathogens in suppressing childhood growth and given that antimicrobials are able to target many of these pathogens, we asked the research question: Would occasional treatment with antimicrobials eliminate enough of the total enteric pathogen burden to increase childhood growth.

We then hypothesized that intervention with antimicrobials (azithromycin and nitazoxanide, both compared to placebo) among a population of children significantly affected by enteric pathogens would increase their linear growth and improve cognitive development.

Primary objective: After intervention with azithromycin (single dose at months 6, 9, 12 and 15) and nitazoxanide (3-day course at months 12 and 15), assess growth response via monthly measure, with primary outcome height-for-age z-score at age 18 months.

Secondary objectives: Assess further endpoints described in the protocol below, related to intestinal pathogen burden, intestinal and systemic inflammation, and alterations in nutrition, metabolism, and hormone regulation.

• Reduction in pathogen infection on Taqman Array Cards at months 3, 6, 6+14 days (i.e., after azithromycin alone), 12, 12+14 days (after both azithromycin and nitazoxanide) and 18

• Alteration of stool microbiota over time

• Sickness via monthly questionnaire (diarrhea, fever, URI, hospitalization)

• Proportion of stunting (HAZ<-2) at 18 months

• Weight-for-age (WAZ) and weight-for-height (WHZ) z-scores at 18 months

• hsCRP (given potential reduction in inflammation)

• IGF-1 (given potential relationships between infection and growth factor production)

• Anemia (given potential effects of infection on iron absorption and metabolism)

• Stool myeloperoxidase

• Cognitive outcomes (Malawi Development Assessment Tool assessment, the Bayley scales of development and the Observation of Maternal and Child Interaction (OMCI) at 12-18 months, described further below)

B. Study Design and Methods

I. Study Design:

Randomized, placebo control, factorial design

**Treatments**

This study tests for effects of administration of nicotinamide/niacinamide (to nursing mothers and young children age 6-18 months) and antimicrobials (to young children 6-18 months), with further details provided in the next section. Justification for these treatments is as follows:

1. Nicotinamide/niacinamide:

Tryptophan is an essential amino acid (i.e., not synthesized by humans) that is critical for protein synthesis and as a precursor of serotonin and niacin. Deficiencies of tryptophan, either by absence of its intraluminal transporter (in Hartnup disease) or a key enzyme (ACE2) cause susceptibility to diarrhea and colitis. These sequelae are related in part to reduced function of mTOR in enterocytes and subsequent reduced secretion of antibacterial dipeptides—with downstream effects on colonic bacteria and inflammation (Figure 5). Intriguingly, among Haydom children studied as part of MAL-ED, higher serum levels of tryptophan were associated with improved subsequent growth,^5^ with an increase in HAZ of 0.29 for those with tryptophan levels at the 90^th^ percentile compared to those at the 10^th^ percentile (p<0.001). While the mechanism behind these growth observations are still not clear, preclinical studies demonstrated 1) that lower levels of tryptophan were associated with reduced growth velocity^19^ and 2) that in low tryptophan conditions, the lower mTOR activity and associated colitis were rescued by nicotinamide/niacinamide, a form of niacin.^20^

Regulation of tryptophan metabolism is related to activity of indoleamine 2,3-dioxygenase (IDO), which increases conversion toward kynurenine. This occurs during infection but also is increased in disease states such as Crohn’s disease, in what in most settings appears to be a maladaptive process. Kynurenine exhibits immune-modulatory activity, including suppressing T-cell activation^21^ and promoting regulatory T-cell development,^22, 23^ with potential immunosuppressive effects.^5^ Among children from Haydom participating in MAL-ED, a higher ratio of kynurenine:tryptophan was associated with higher systemic inflammation, including higher levels of interferon-α, IL-6 and C-reactive protein.^5^ Moreover, in Peruvian children, high kynurenine levels were associated with a decreased response to oral vaccines, suggesting that a high IDO activity in this setting may overall produce a maladaptive immune response.

Kynurenine is further metabolized toward niacin. Niacin itself is a critical cofactor for production of nicotinamide adenosine dinucleotide (NAD+), which is required for cellular functions such as generation of ATP. Significant dietary deficiencies of niacin result in pellagra, which is also associated with diarrhea. Niacin deficiency is frequently seen in areas with suboptimal niacin intake that include maize and/or sorghum-based diets, the former being common in the Haydom area. While niacin and tryptophan intake were not assessed in Haydom, there is a strong likelihood of suboptimal intake given the multiple micronutrient deficiencies that were observed.

A separate study from MAL-ED used a metabolomics approach to identify metabolic variation associated with growth in children from northern Brazil.^24^ Children excreting greater amounts of *N*-methylnicotinamide (NMND), a metabolite of nicotinamide, exhibited higher subsequent growth (see Figure 5).^24^ Stunted children were found to excrete greater amounts of indole metabolites, bacterial products of tryptophan metabolism (Figure 5), than their non-stunted equivalents as well as other metabolites derived from the gut microbial metabolism of amino acids. These alterations indicate a shift in the functional status of the gut microbiota among children with malnutrition.^24^ These results demonstrate the utility of applying metabolic profiling approaches to investigate the biochemical modulations induced by interventions including the critical metabolic pathways outlined above.


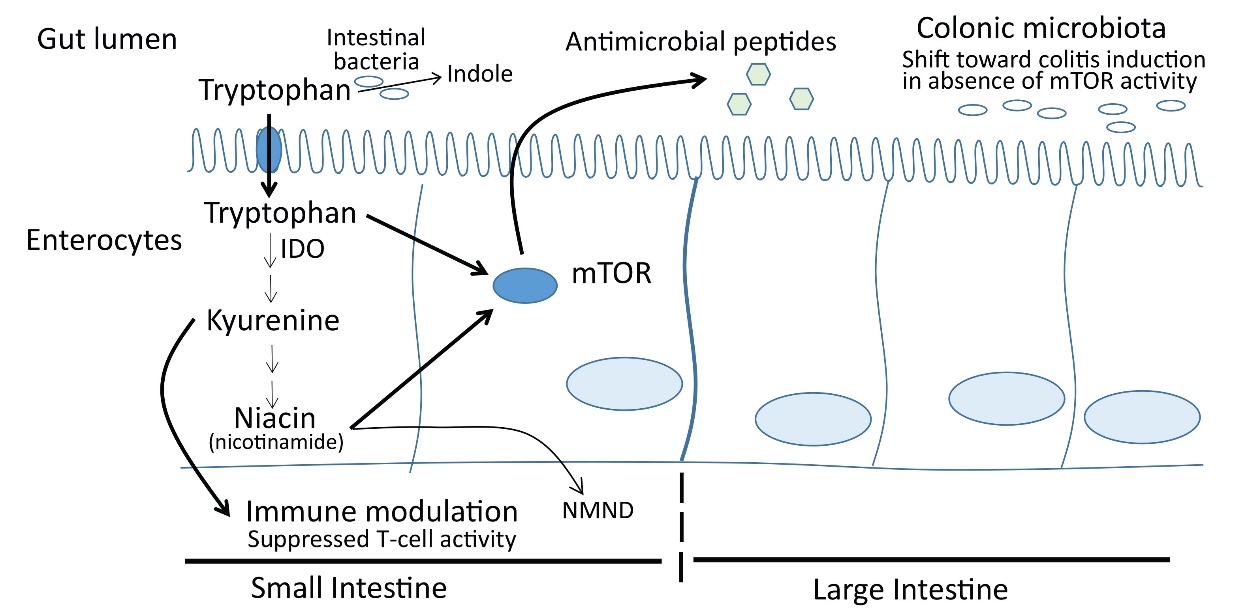


**Figure 3: Schematic of the tryptophan-nicotinamide pathway in the intestinal lumen and mucosa.**

Overall, the relationships of the tryptophan-kynurenine-nicotinamide pathway with enteric microbiota and inflammatory response appear in most cases to favor higher levels of tryptophan, lower levels of kynurenine and higher levels of nicotinamide toward sustaining more beneficial enteric conditions. Administration of high doses of nicotinamide, as we propose, would be hypothesized to overall shift the equilibrium away from catabolism of tryptophan to kynurenine, thus resulting in increased levels of both nicotinamide and tryptophan—and thereby reducing enteral and systemic inflammation. It is our hypothesis that an intervention using high-dose nicotinamide/niacinamide will increase linear growth and reduce local and systemic inflammation via reduction of kynurenine-related immunomodulation and increase in mTOR activity and downstream effects on colonic microbiota.

Nicotinamide/ niacinamide intervention description:

Mothers in the niacin intervention will be given nicotinamide/niacinamide 250 mg (or placebo) daily from delivery through 6 months post-partum resulting in breastmilk niacin transfer during breastfeeding. Nicotinamide/niacinamide 100 mg daily (or placebo, both as a powder added to food or fluid) will then be administered to children from 6-months through 18-months of age. Training on nicotinamide administration and adherence will be provided to mothers. To assess adherence, the study team will evaluate unused quantities of nicotinamide and administer an adherence questionnaire during home follow-up visits.

The infant nicotinamide dose is based on the upper end of the advisable range^25^ and the recommended dose for low niacin conditions.^26^ Adult safety data on nicotinamide has been established in its use assessing for prevention of auto-immune diseases such as Type 1 diabetes.^27, 28^ Long-term administration has been performed in otherwise-healthy adults in doses as high as 3 g without untoward effects.^27, 28^ These doses have been extrapolated to safe ingestion amounts of 150 mg daily for 1-year-old children as part of the European Commission.^25^ In a pediatric study of otherwise-healthy children at risk for Type 1 diabetes, doses of 1.2 g/m^2^ were administered to young children without problem.^29^ Based on body size, this corresponds to a dose of approximately 400 mg for a 6 month-old child (i.e., 4 times the dose we propose). Another study gave otherwise-healthy children with ADHD 3 g of nicotinamide daily (i.e., >10 times the dose we propose, factored on a per-body-weight basis). Of note, nicotinamide does not result in flushing symptoms, as nicotinic acid can produce at high doses, nor does it cause a lowering of LDL cholesterol levels.^26^ Excess nicotinamide is metabolized by methylation in the liver to form *N*-methylnicotinamide (NMND), which does not have biological activity and is a polar, water-soluble excretory product.^25^ High doses of niacin are excreted in the urine, as nicotinic acid and its glycine conjugate (nicotinuric acid), thus we anticipate that there will be safe and efficient elimination of any excess niacin.^30, 31^

Nicotinamide is present in breast milk in quantities that are associated with serum levels.^32^ In Malawian women given 20 mg/d of niacin equivalents in a lipid-based nutritional supplement, relative increases of breastmilk nicotinamide of 130 ug/L at 2-6 weeks and 60 ug/L at 24 weeks lactation were reported.^32^ While nicotinamide concentrations in breast milk may be linearly related to supplemental dose, there is likely a threshold effect upon which higher maternal doses will not result in greater breastmilk transfer. Extrapolating from the Malawian study and assuming perfect linearity and lack of threshold regulation, maternal nicotinamide doses of 250 mg/d are estimated to further increase the breast milk concentrations by approximately 1950 ug/L at 2-6 weeks and 900 ug/L at 24 weeks. Based on the estimated total mean nicotinamide breastmilk concentration (combined baseline mean unsupplemented concentration from Malawian study of 219 to 430 ug/L plus the supplemented contribution as proposed in this study), we estimate supplemented women in this study would have an approximate mean breastmilk concentration ranging from 2380 to 1119 ug/L at 6 and 24 weeks, respectively. Considering a mean milk consumption of about 0.8 L/d during the 6-month exclusive breastfeeding period, this would represent between 895 and 1904 ug/d total consumption in exclusively breastfed infants up to 6-months of age in the nicotinamide study arms, compared to the National Academy of Medicine reference Adequate Intake for infants from birth to 6-months of 2000 ug/d.^33^

Given the above, we propose a maternal niacin dose of 250 mg/d as nicotinamide (or placebo) for the first 6-months postpartum and niacin supplementation of 100 mg/d as nicotinamide (or placebo) for children between 6 and 18 months of age. To ensure infant safety of indirect nicotinamide transfer during breastfeeding 0-6 months and directly during infant nicotinamide administration from 6 to 18 months, we will perform serum testing including basic metabolic panel, liver function assessments and complete blood count on a subset of children at or about 2 and 8 months and at study end to verify tolerability and lack of significant unanticipated symptoms with data presented to the DSMB.

2. Antimicrobials:

A meta-analysis has revealed that antimicrobials have a growth promoting effect in young children in low and middle income countries, more pronounced for weight than for linear growth, with effect sizes of ~24 g/mo and 0.04 cm/mo respectively.^34^ These data derive from similar settings to Tanzania, such as Niger (though no additional increase in growth was observed whether given once or twice per year).^35^ The mechanism of such a growth effect is also unclear but has been hypothesized to be due to treatment of subclinical enteric infections or alteration of the microbiome. Mass distribution of azithromycin has been associated with reduced mortality in children in Ethiopia.^6^ Given our MAL-ED data showing high rates of bacterial enteropathogen carriage (*Campylobacter*, EAEC, ETEC, aEPEC), organisms susceptible to azithromycin, we have an excellent basis to expect a growth increase. We also have preliminary data from Christian Medical College, Vellore, India (our colleagues in the MAL-ED study), showing that treatment with a single dose of azithromycin (compared to placebo) within 14 days results in the expected decrease in infection rates of relevant pathogens (as measured by TaqMan Array Cards (TAC)). Please see the Response to Reviewer section for discussion regarding any potential risk associated with distribution of antimicrobials. Regarding the timing of administration, our data from MAL-ED demonstrated that at age 6 months, the proportion of children in the Haydom area testing positive at age 6 months for *Campylobacter* was 31.7% and for EAEC 47%. We consider this prevalence high enough that intervention with azithromycin at would likely be beneficial for a large proportion of the population starting at age 6 months. However, re-exposure could result in rapid re-colonization with pathogens. We will thus repeat treatment every 3 months.

We will also add nitazoxanide to the study because of its additive antimicrobial properties, particularly for protozoa such as *Cryptosporidium* and *Giardia* which were found at high rates in this cohort and have also been associated with poor linear growth. Nitazoxanide has been approved for pediatric use to as young as 12 months, thus we will administer this at 12 and 15 months. Findings from randomized studies have shown a beneficial effect of nitazoxanide in cryptosporidiosis without HIV (reviewed in reference ^36^) including a significant reduction in mortality in malnourished children with cryptosporidial diarrhea in Zambia.^37^ We will evaluate TaqMan array cards to assess stool pathogens over the course of the intervention, both before azithromycin dosing, 2-weeks after azithromycin, before nitazoxanide dosing, 2-weeks after nitazoxanide and at study completion.

Antimicrobial intervention administration: Children in the sites randomized to receive the antimicrobial intervention will be given azithromycin 20 mg/kg (200 mg/5ml oral suspension, rounded to the nearest mL, administered by spoon or syringe by study personnel) or placebo at 6, 9, 12 and 15 months, nitazoxanide 100 mg (5 mL oral suspension) or placebo twice daily x 3 days at 12 and 15 months or neither (control group). In addition to witnessed administration of nitazoxanide at study visit, families will be given containers with 2 additional doses to be given in the 2 subsequent days. These drug dosages are US-FDA and Tanzanian-FDA approved at these age groups.

Study duration: For this phase, study completion is planned in 3 years where, 25% will be enrolled in the first year and 100% enrolled in two years. With adequate funding, follow-up will continue yearly through age 9 years.

II. Setting:

This study will be conducted at the Haydom Global Health Research Centre at Haydom Lutheran Hospital. We will recruit mother/child pairs in the approximately a 20 km radius of Haydom Lutheran Hospital.

III. Research participants:

Participants consist of pregnant women (at recruitment) of 18 years and above and later the mother/child dyad until testing effects of treatment through age 18 months, with long-term follow-up through age 9 years. As described further below, we are testing 2 intervention arms which are randomized in a factorial design such that ½ of the mother/child dyads will receive each of the interventions and ½ will be controls for that arm. We expect to recruit 1188 pregnant women with expectation of 5% screening failure, 5% dropout and withdraw and about 1080 children and their mother complete study participation.

**Inclusion criteria:**

1. Maternal age >18
2. Current pregnancy or infant </=14 days.

**Exclusion criteria:**

1. Maternal inability to adhere to protocol.
2. Multiple gestation.
3. Childhood illness (significant birth defect, severe neonatal illness, hospitalization for other than healthy birth).
4. Birth weight <1500 g.
5. Lack of breastfeeding at enrollment (and lack of intention to continue breastfeeding at time of enrollment).

As mentioned previously, the overall purpose of this study is to determine the efficacy of two different interventions on specific outcomes related to childhood growth, enteric infection and cognitive development. Participants will be recruited as pregnant women or as mothers of an infant age </= 14 days. Interventions and outcomes are split between the mothers and the children. Thus, mothers will be recruited as a dyad of mother and child.

The primary outcome is difference in HAZ at 18 months. The sample size was calculated to provide sufficient power for testing the main effects for the two interventions in a 2 x 2 factorial design. If each main effect were tested at the 5% levels, as is customary in the analysis of factorial studies,^42, 43^ with 270 participants per group, there is 80% power for a difference in HAZ of 0.176. This is within the range of improvement of an increase of 0.16 seen in a prior study after 12 months of micronutrient supplementation,^44^ though in Haydom the growth and deficiencies are more severe than that site, thus the potential for greater effect size. The interaction between main effects will then be estimated and tested as a secondary analysis. If we were to take a more conservative approach and use a significance level of 2.5% for each of the main effects, with 270 subjects per group, we would still have 80% power for an average HAZ increase of 0.193. Finally, if we were to adjust the significance to include the test for the interaction, the F-test for the main effect in a 2-way ANOVA has 80% power with a two-sided significance level of 1.67%, when the main effect of the intervention is to increase the mean HAZ by 0.203. All of these are reasonable changes in HAZ to observe for in the Haydom population, as significant as the mean deficit in growth is. Adjusting for 10% dropout, the total sample size required is 1188. Within our recruitment radius are >2507 births occur yearly.  We will recruit during an 8-month window.  In our extensive research experience at the Haydom MAL-ED site, we observed 0% refusal to participate and a high 81% retention rate by 4 years of follow-up during MAL-ED (and, thus, a study of shorter duration may have improved follow-up).  Based on this, we anticipate a high rate of recruitment (>90%) and 18-month retention (>90%). Therefore, accounting for drop-out, we anticipate achieving 1188 mother/child dyads divided between intervention and control, leaving us adequate power to determine differences in HAZ. In addition to standard 2-way ANOVA to assess main effects and the interaction, linear and logistic regression will be used to evaluate the effect of the individual intervention domains on outcomes after taking into account individual maternal and child characteristics such as SES, maternal height and enrollment weight as covariates.

**General description of field teams and randomization scheme:**

*Intervention Field Teams:* We will use research field workers and community health care workers in the neighborhoods in a 20 km radius of Haydom Lutheran Hospital to survey for pregnancies (last trimester) in the area and alert the field team. The field team members will then approach the household and explain the study and offer the option of participating. Families who provide informed consent will be randomized at the time of recruitment to be in one of the four treatment groups. The community health care workers will be made aware of the enrollment and will subsequently survey for timing of birth and then contact the study team for a visit within the first two weeks after birth for entry anthropometry. Mother/child dyads randomized to the nicotinamide/niacinamide intervention will be given a two-month supply of nicotinamide/niacinamide to start, as described further below. The field team will then visit the house at least monthly until 18 months of age

*Randomization Scheme:* We will randomize our interventions in a factorial design, using computer-generated numbers on an individual participant basis. This provides 4 different combinations of interventions (Figure 3) such that each intervention is implemented in 50% of children overall. On a more granular level, 25% of children will receive both interventions, 25% will receive only antimicrobials, 25% will receive only nicotinamide and 25% will receive no intervention. With this approach, we will be able to independently assess our individual intervention domains and outcomes. This factorial design may also allow for analysis of additive or synergistic effects of the combinations of these interventions.

IV. Timing of treatments and evaluations:

This next portion of the protocol focuses on what these interventions are, when the treatments are given and how the related outcomes are measured. The overview of this is shown in the Figure 1, with details explained in the question further below.


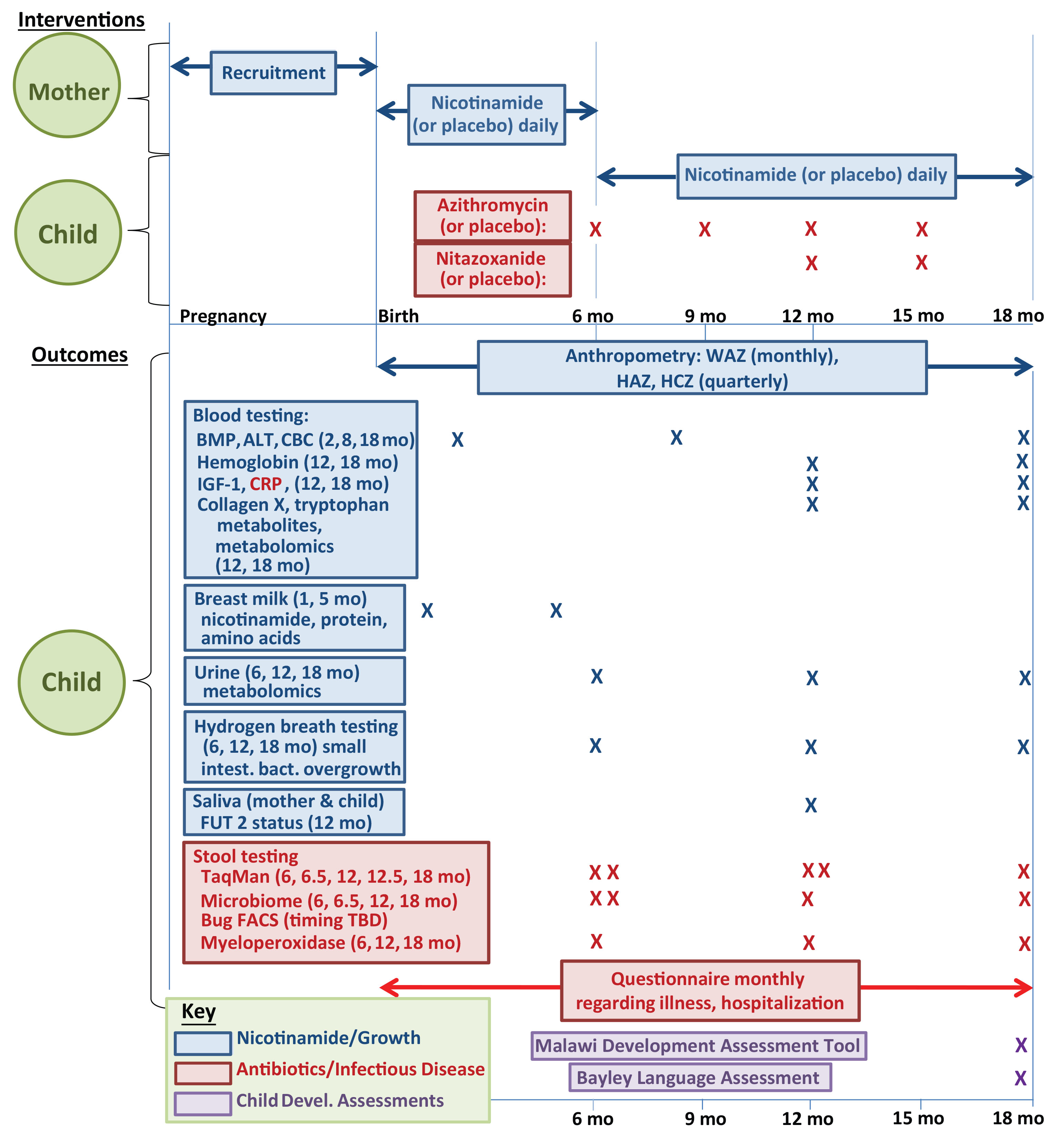


**Figure 4: Intervention and assessment schematic through 18 months. Children will then be followed at yearly intervals until age 9 years.**

The treatment arms are:

1. Nicotinamide/niacinamide group
2. Provision of nicotinamide/niacinamide (vitamin B6) 250 mg daily to mother during lactation 0-6 months
   - 1. These will be provided to mothers each house visit for daily use with instructions to take with fluid daily.
     2. These will be given at the initial study visit and subsequent visits thereafter through 6 months of age.
3. Provision of nicotinamide/niacinamide powder 100 mg daily for children from months 6-18
   - 1. These will be provided to mothers to start daily use at 6 months of age with instructions to administer to child mixed with food or fluid daily.
     2. These will be given at the 6-month study visit and subsequent visits thereafter through 18 months of age.
4. Control group: Mothers and children in this group will receive placebo and no additional intervention beyond usual care.
5. Antimicrobial group (receiving azithromycin and nitazoxanide). Children in this group will be given:
   1. Azithromycin 20 mg/kg, administered by study personnel) at 6, 9, 12 and 15 months
   2. Nitazoxanide 100 mg twice daily x 3 days at 12 and 15 months. The first dose of the 3-dose course will be administered by study personnel; the subsequent doses will be supplied to the mother in small containers with instructions to administer to the child at home on the subsequent 2 days. Mothers will be asked at the next visit whether they were able to administer these doses.
   3. Mothers will be told to report any new rash or other reaction following antibiotic dose to their neighborhood healthcare representative (study staff), who will then contact the central study staff.
      1. Child will be brought to pediatric clinic at Haydom Lutheran Hospital for evaluation
      2. These data will be reported to DSMB

Control group: Children in this group will receive placebo and no additional treatment beyond usual care.

Re-iterating a point from above, depending on the randomization of the mother/child dyads, each dyad will be assigned to anywhere from no interventions to both intervention domain described above.

**Outcome measures**

The outcome measures will be performed on all mother/child dyads, regardless of the intervention arm to which they are randomized.

General data collected:

- - - 1. Socioeconomic data
         1. By questionnaire, mothers will be asked regarding number of children, number of family members living in home, maternal and paternal occupation, and family income and assets

Data collection related to specific domains:

1. Nutrition/growth
   1. Weight
      1. Mother’s weight will be measured on standing scale
      2. Child’s weight will be measured using recumbent scale or standing scale as appropriate
   2. Linear growth
      1. Mother’s height will be measured using stadiometer
      2. Child’s length will be measured using Schor board monthly until age 18 months and then yearly until age 9 years
      3. Child’s anthropometry will additionally be measured in a subset of participants using the AutoAnthropometry device by BST, Inc. We will take 3‐D pictures using a commercially available scanner, as shown below.


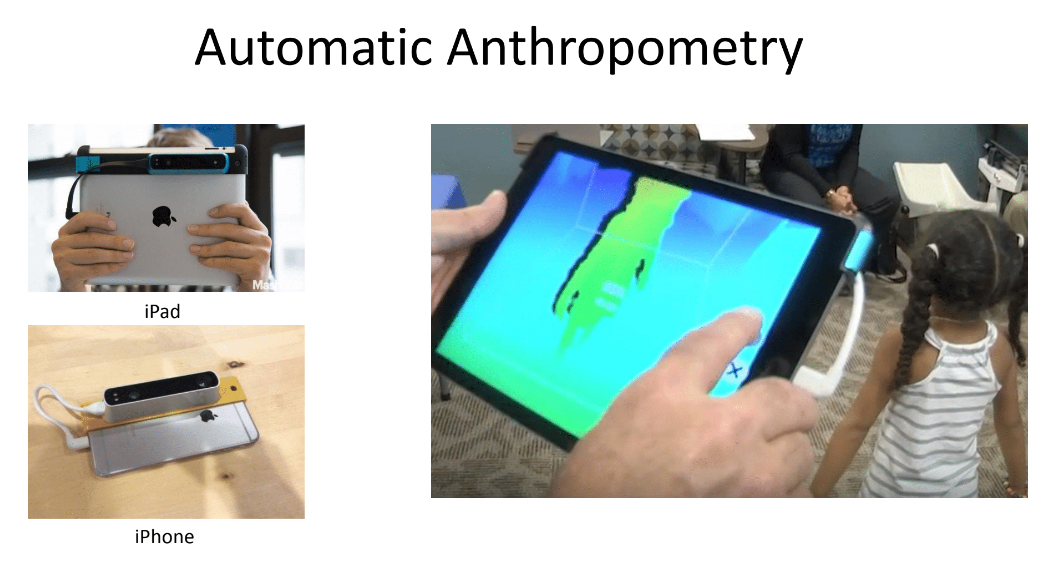


The 3‐D pictures will be developed by shining a very low-powered near-infrared light source at the child and then taking a series of pictures. The technology used is identical to the Microsoft Kinect device, used in millions of homes around the world to play video games. In this application, we shine the light on the child, take a series of snap‐shots from different angles, then using the computer develop a three-dimensional model of the child. The child is not identifiable from the 3D scans. The process is very similar to taking a photograph under a very weak, invisible strobe light.

The resulting scans look this image:


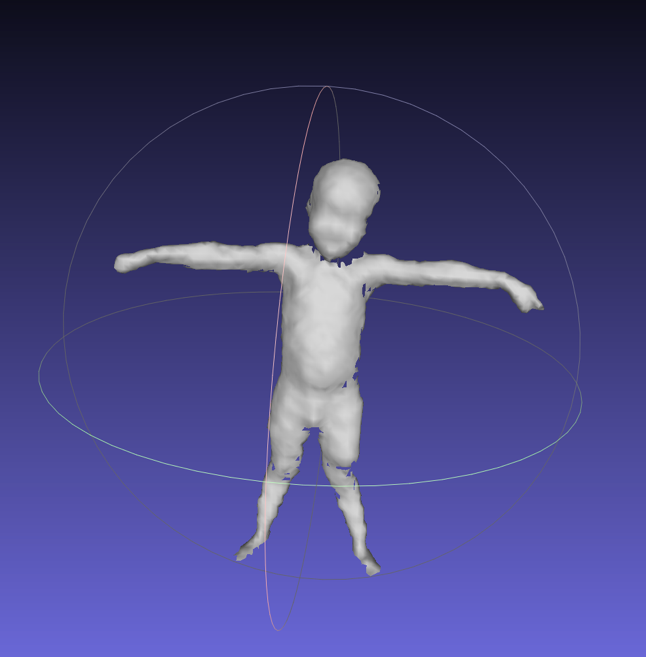


These images will then be used to digitally assess anthropometry and will be compared to the anthropometric measures by traditional methods to calibrate the system among children in the Haydom region. This system has been approved by IRBs in the United States, Guatemala, Kenya, and the Philippines; thus far, the system has required additional calibration in each setting, such as we propose to do here. The iPad and the Structure Sensor have UL Safety and CE Mark certifications.

- 1. Questionnaire monthly regarding breast feeding exclusivity, introduction of foods, and adherence to treatment
  2. Counting of leftover maternal vitamins and childhood multi-nutrient doses
  3. Phlebotomy at or about 2 and 8 months of age and study end (subset of participants) for measure of safety, including basic metabolic panel, liver function tests and complete blood count, and at or about ages 12 and 18 months (full cohort) and yearly thereafter (funding permitting) for measure of health, growth factors, inflammatory factors, hemoglobin, metabolomics and metabolites related to the tryptophan-nicotinamide pathway
     1. Phlebotomy will be performed by trained healthcare worker
     2. Maximum blood volume collection will be 3 mL/kg at each blood draw, and blood draws will be separated by at least 4 months, as shown below:

| **Child’s age at blood draw** | **Reason for blood draw** | **Volume of blood draw** |
| --- | --- | --- |
| **2 months (subset of children)** | Assess basic metabolic panel, liver function tests and complete blood count | Maximum 3 mL/kg |
| **8 months (subset of children)** | Assess basic metabolic panel, liver function tests and complete blood count | Maximum 3 mL/kg |
| **12 months** | Assess study outcomes related to nutrition/health, metabolism and inflammation | Maximum 3 mL/kg |
| **18 months (subset of children)** | Assess basic metabolic panel, liver function tests and complete blood count | Maximum 3 mL/kg |
| **18 months** | Assess study outcomes related to nutrition/health, metabolism and inflammation | Maximum 3 mL/kg |

From these, blood will either be tested within one week of draw (in the case of the basic metabolic panel, liver function tests and complete blood count) or will be separated into serum and stored.

- - 1. Serum will be batched and stored at -80 until time of testing

1. Antimicrobial-related outcomes
   1. Stool samples for laboratory testing for bacterial colonization and infection
      1. Stool will be collected monthly and evaluated for pathogens and microbiota at months 3, 6, 6+14 days, 9, 12, 12+14 days, 15 and 18 and yearly thereafter
      2. Samples will be batched and stored at -80 degrees
      3. Testing will include TaqMan array cards, culture and microbiota analysis
   2. Questionnaires regarding symptoms of illness
      1. Mothers will be asked regularly regarding interval symptoms of illness in their child
      2. Questions concerning diarrhea, bloody stools, fevers, cough, antibiotic use and other
   3. Exhaled hydrogen
      1. Using a standard protocol,^41^ exhaled hydrogen will be measured to assess the degree of small intestinal bacterial overgrowth in a subset of children around age 6, 12 and 18 months. As in a prior protocol approved by the UVa HSR-IRB,^41^ this will only be performed in children with a WAZ >-3. For this testing the child will be required to fast with only water allowed during the fasting period. Children below 1 year of age will fast for 2 hours and children older than 1 year of age will fast for 3 hours prior to beginning testing. After the fast, the child will be given a 1g/kg (5ml/kg) of glucose solution. Hydrogen breath chromatography will be measured prior to administration of the glucose solution and then every 20 minutes for 3 hours after solution administration. Breath samples will be collected using a anesthesia mask with 1-way valve and bag (as previously), along with the Quintron Breath Sampler Neonate and Infant System and samples will be analyzed using the Quintron BreathTracker SC breath chromatograph.
   4. Surveillance for complications from antibiotic use
      1. As described above under interventions for antimicrobial domain, data will be collected regarding observed symptoms following antimicrobial administration, including if the child has had a health care visit and results of subsequent clinical evaluation
2. Child development
   1. Children will undergo testing using developmental assessment scales by trained staff at 12-18 months and yearly until age 9 years.
3. Maternal health, infection/inflammation, nutrition and metabolism
   1. Blood will be drawn from mothers to assess metabolomics and markers of infection/inflammation, nutrition and general health.
   2. Stool will be collected from mothers to assess metabolism and markers of infection/inflammation, nutrition and general health

**Timing of outcome measures**

Initial consent:

Mothers will be screened during pregnancy or up to infant age of 2 weeks. Eligibility will be confirmed at child age up to 2 weeks (based on infant weight). Following confirmation of eligibility, mothers will consented and randomized. Treatment with nicotinamide/niacinamide or placebo will start immediately after the initial enrollment.

Newborn visit:

1. Questionnaire regarding birth history: date, weight (if performed), birth complications, interval medical problems.
2. Breast feeding, other complementary food history thus far.

Monthly visits during ages 1-6 months:

1. Breast feeding, other complementary food history thus far.
2. Questions regarding general health, diarrhea/fever/cough/hospitalization
3. Adherence to nicotinamide/niacinamide, both maternal (months 1-6) and child (months 7-18)
4. Frequency of parental use of the development items (at 6 months only).

Specific months with additional items covered during visits, with approximate timing:

1, 3, 5

1. Breast milk collection

2, 8, 18 months:

1. Blood drawn in sub-set for basic metabolic panel, liver function tests, complete blood count

3 months:

1. Stool collection

6 months:

1. Urine collected

2. Breath test performed to evaluate for small intestine bacterial overgrowth

12 month:

1. Blood drawn.
2. Hemoglobin point-of-care
3. Urine collected
4. Saliva sample collected (child and mother).
5. Breath test performed to evaluate for small intestine bacterial overgrowth

12-18 months:

1. Malawi Development Assessment Tool
2. Observation of Maternal & Child Interaction
3. Bayley scales of development
4. Each of the above scores may be video recorded for later scoring by study personnel. These video recordings may be uploaded to the secure study database for QC purposes. Participants will always be informed when video recording is performed and resultant files will be destroyed upon study completion.

18 months:

1. Updated family socioeconomic data
2. Stool sample collected.
3. Blood drawn.
4. Urine collected.
5. Saliva sample collected (if not done previously).
6. Breath test performed to evaluate for small intestine bacterial overgrowth

Stool samples will be collected at months 3, 6, 6.5, 9, 12, 12.5, 15 and 18 by distribution of diapers and plastic bags the month prior or by the community health representative in the days before the visit.

With the exception of the 18 month Visit, other notable months’ treatments or assessments may vary by 1 month; the timing of this will be taken into account in the analysis.

As funding permits, participants will then be followed yearly after the study for assessments of anthropometry, blood pressure, blood and urine tests of nutrition, metabolism and general health, and developmental testing including tests such as the Malawi Development Assessment Tool, the Bayley scales of development, the Hammersmith Infant Neurological Exam (HINE), the Peabody Developmental Motor Scales, the Developmental NEuroPSYchological assessment (NEPSY), the Weschsler Preschool and Primary Scale of Intelligence, and the Wechsler Intelligence Scale for Children, and potentially tablet-based developmental assessments.

V. Ethical considerations:

**The authorities in the area where the study will be conducted will be informed about the study and permission to conduct the study will be requested from the regulatory institution i.e. National Institute for Medical Research (NIMR) in Tanzania and University of Virginia IRB in the US. The local leaders in the study catchment area with be informed about the study in the awareness meeting. The member of the community will be informed about the study during surveillance and screening and those found eligible will go through the consenting process when those who will voluntarily consent after understanding about the study will have to sign the consent document before any study procedure is taken to them. For the potential participant who are illiterate, they will be allowed to identify an impartial witness who will participate in the consenting process and if the participant is will after understanding all about the study, then will mark with a thumb print the impartial witness sign the consent.**

**There is a Data Safety and Monitoring Board (DSMB) as described below.**

DSMB: A data and safety monitoring board will be formed to review data regarding safety of antibiotics. This board will contain experts and physicians with specialties in infectious disease, global health, pediatrics, and statistics to assist with reviewing data related to adverse events, health outcomes and surveillance cultures evaluating for antibiotic resistance. The DSMB will then make recommendations regarding ongoing safety of the interventions and any need to discontinue one/any of the treatment arms.

Data and Safety Monitoring Board members:

1. Jim Todd, Ph.D

Professor of Applied Biostatistics

London School of Hygiene & Tropical Medicine

(Lives and works in Mwanza-BMC/Moshi-KCM College)

1. Sean Moore, MD, MPH

Associate Professor of Pediatrics

University of Virginia

(Pediatrician/GI specialist/global health investigator)

1. Donna Denno, MD, MPH

Professor of Pediatrics and Global Health

University of Washington

            (Pediatrician and international health researcher)

1. Prof. Blandina Mmbaga MD, MMED, PhD

Pediatrician, Researcher and lecturer (KCMC, KCRI and KCMU College

(Director– KCRI). Moshi, Tanzania

VI. Statistical considerations:

*Randomization Scheme:* We are employing a factorial design such that each intervention will be assigned independently. We will randomize our intervention domains separately on an individual basis. This provides 4 different combinations of interventions (Figure 1).


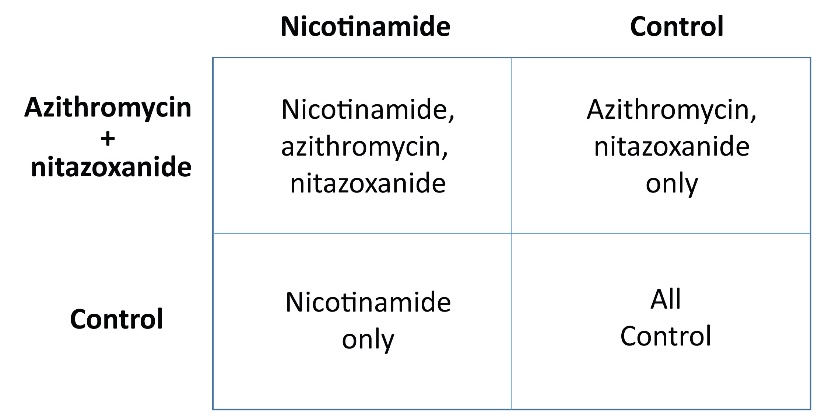


**Figure 5: Randomization scheme.**

1. **Statistical considerations for the protocol:**

The primary outcome is difference in HAZ at 18 months. The sample size was calculated to provide sufficient power for testing the main effects for the two interventions in a 2 x 2 factorial design. If each main effect were tested at the 5% levels, as is customary in the analysis of factorial studies,^42, 43^ with 270 participants per group, there is 80% power for a difference in HAZ of 0.176. This is within the range of improvement of an increase of 0.16 seen in a prior study after 12 months of micronutrient supplementation,^44^ though in Haydom the growth and deficiencies are more severe than that site, thus the potential for greater effect size. The interaction between main effects will then be estimated and tested as a secondary analysis. If we were to take a more conservative approach and use a significance level of 2.5% for each of the main effects, with 270 subjects per group, we would still have 80% power for an average HAZ increase of 0.193. Finally, if we were to adjust the significance to include the test for the interaction, the F-test for the main effect in a 2-way ANOVA has 80% power with a two-sided significance level of 1.67%, when the main effect of the intervention is to increase the mean HAZ by 0.203. All of these are reasonable changes in HAZ to observe for in the Haydom population, as significant as the mean deficit in growth is. Adjusting for 10% dropout, the total sample size required is 1188. Within our recruitment radius are >2507 births occur yearly.  We will recruit during an 8-month window.  In our extensive research experience at the Haydom MAL-ED site, we observed 0% refusal to participate and a high 81% retention rate by 4 years of follow-up during MAL-ED (and, thus, a study of shorter duration may have improved follow-up).  Based on this, we anticipate a high rate of recruitment (>90%) and 18-month retention (>90%). Therefore, accounting for drop-out, we anticipate achieving 1188 mother/child dyads divided between intervention and control, leaving us adequate power to determine differences in HAZ. In addition to standard 2-way ANOVA to assess main effects and the interaction, linear and logistic regression will be used to evaluate the effect of the individual intervention domains on outcomes after taking into account individual maternal and child characteristics such as SES, maternal height and enrollment weight as covariates.

**2. Justification for the sample size used in this protocol:**

See above response (Question 2). In our extensive research experience at the Haydom MAL-ED site, we observed 0% refusal to participate and a high 81% retention rate at 4 years. Based on this, we anticipate a high rate of recruitment (>85%) and retention (>90%). We also employ Home Based Care workers to assist with participant retention. Therefore we anticipate easily achieving 1,188 mother/child dyads divided between intervention and control, leaving us adequate power to determine differences in HAZ.

**3. Plan for primary variable analysis:**

The primary outcome for the overall study is HAZ at 18 months. For each of the intervention domains we will compare this outcome between those randomized to receive the intervention vs. those who did not. This will be performed using a modified intent-to-treat analysis, assessing participants who complete the study and have a measurement of HAZ at 18 months.

**4. Plan for secondary variable analysis:**

Each of the intervention domains has its own set of secondary outcomes as well, and these will be compared between those who receive the intervention and those who do not. This will initially be performed using the same modified intent-to-treat analysis assessing those with the individual outcome of interest. Secondary analysis for all endpoints (including HAZ at 18 months) will also be performed using per-protocol treatment, defined as participants meeting the following criteria:

- Breast fed through age 6 months
- Received all doses of azithromycin
- Received initial dose of nitazoxanide
- Received at least 50% of nicotinamide doses.
- Have the outcome of interest listed.

Secondary analysis outcomes are as follows:

Nicotinamide/niacinamide domain:

- Proportion of stunting (HAZ<-2) at 18 months
- Weight-for-age (WAZ) and weight-for-height (WHZ) z-scores at 18 months
- HAZ at yearly visits from age 2-9 years
- Metabolomic assay of gut microbial metabolism and alterations to tryptophan-niacin and other pathways at 6, 12 and 18 months
- Tryptophan:kynurenine ratio and other pathway metabolites in serum at 12 and 18 months
- IGF-1 at 12 and 18 months
- High-sensitivity C-reactive protein at 12 and 18 months
- Stool myeloperoxidase at months 6,12 and 18 months
- Anemia—proportion of children with moderate anemia as defined by WHO for age and altitude^45^
- Prevalence of enteric pathogens at months 6, 6+14 days, 12, 12+14 days, and 18
- Change in microbiota (by traditional sequencing) at months 6, 6+14 days, 12 and 18 (subset of participants)
- Change in microbiota (using Bug FACS) at months 6, 6+14 days, 9, 12, 15 and 18 (subset of participants)
- Change in small intestinal bacterial overgrowth as assessed by exhaled increased hydrogen following ingestion of sugar (subset of participants).
- Difference in breast milk composition (nicotinamide, tryptophan, amino acids, human milk oligosaccharides (HMO), docosahexaenoic acid (DHA)
- Symptomatic diarrhea
- Hospitalization and all-cause mortality
- Cognitive outcomes Malawi Development Assessment Tool (MDAT)^40^ assessment and the Observation of Maternal and Child Interaction (OMCI)^59^ at 15 months, given the importance of nutrition and enteric status for the developing brain^46^

Antibiotic domain:

- Reduction in pathogen infection on TAC at months 6, 6+14 days (i.e., after azithromycin alone), 12, 12+14 days (after both azithromycin and nitazoxanide) and 18
- Alteration of stool microbiota over time
- Change in small intestinal bacterial overgrowth as assessed by exhaled increased hydrogen following ingestion of sugar (subset of participants).
- Sickness via monthly questionnaire (diarrhea, fever, URI, hospitalization)
- Proportion of stunting (HAZ<-2) at 18 months
- Weight-for-age (WAZ) and weight-for-height (WHZ) z-scores at 18 months
- hsCRP (given potential reduction in inflammation)
- IGF-1 (given potential relationships between infection and growth factor production)
- Anemia (given potential effects of infection on iron absorption and metabolism)
- Stool myeloperoxidase
- Cognitive outcomes (Malawi Development Assessment Tool assessment^40^ and the Bayley Scales of Childhood Development at 18 months, described further below)

C. References

1. Jones, G: Pharmacokinetics of vitamin D toxicity. *American Journal of Clinical Nutrition,* 88**:** 582S-586S, 2008.

2. Bhutta, ZA: Early nutrition and adult outcomes: pieces of the puzzle. *Lancet,* 382**:** 486-487, 2013.

3. Victora, CG, Adair, L, Fall, C, Hallal, PC, Martorell, R, Richter, L, Sachdev, HS: Maternal and child undernutrition: consequences for adult health and human capital. *Lancet,* 371**:** 340-357, 2008.

4. Adair, LS, Fall, CH, Osmond, C, Stein, AD, Martorell, R, Ramirez-Zea, M, Sachdev, HS, Dahly, DL, Bas, I, Norris, SA, Micklesfield, L, Hallal, P, Victora, CG, group, C: Associations of linear growth and relative weight gain during early life with adult health and human capital in countries of low and middle income: findings from five birth cohort studies. *Lancet,* 382**:** 525-534, 2013.

5. Kosek, MN, Mduma, E, Kosek, PS, Lee, GO, Svensen, E, Pan, WKY, Olortegui, MP, Bream, JH, Patil, C, Asayag, CR, Sanchez, GM, Caufield, LE, Gratz, J, Yori, PP: Plasma tryptophan and the kynurenine tryptophan ratio are associated with the acquisition of statural growth deficits and oral vaccine underperformance in populations with environmental enteropathy. *American Journal of Tropical Medicine & Hygiene,* under review, 2016.

6. Wiggs, L: Behavioural aspects of children's sleep. *Archives of Disease in Childhood,* 94**:** 59-62, 2009.

7. Grimes, CA, Riddell, LJ, Campbell, KJ, Nowson, CA: Dietary Salt Intake, Sugar-Sweetened Beverage Consumption, and Obesity Risk. *Pediatrics,* 131**:** 14-21, 2013.

8. Brill, DS, Moenter, SM: Androgen Receptor Antagonism and an Insulin Sensitizer Block the Advancement of Vaginal Opening by High-Fat Diet in Mice. *Biol Reprod*, 2009.

9. Pappa, HM, Mitchell, PD, Jiang, H, Kassiff, S, Filip-Dhima, R, DiFabio, D, Quinn, N, Lawton, RC, Varvaris, M, Van Straaten, S, Gordon, CM: Treatment of Vitamin D Insufficiency in Children and Adolescents with Inflammatory Bowel Disease: A Randomized Clinical Trial Comparing Three Regimens. *Journal of Clinical Endocrinology &amp; Metabolism,* 97**:** 2134-2142, 2012.

10. Guerrant, RL, Oria, RB, Moore, SR, Oria, MO, Lima, AA: Malnutrition as an enteric infectious disease with long-term effects on child development. *Nutr Rev,* 66**:** 487-505, 2008.

11. Guerrant, RL, Kosek, M, Lima, AA, Lorntz, B, Guyatt, HL: Updating the DALYs for diarrhoeal disease. *Trends Parasitol,* 18**:** 191-193, 2002.

12. Guerrant, DI, Moore, SR, Lima, AA, Patrick, PD, Schorling, JB, Guerrant, RL: Association of early childhood diarrhea and cryptosporidiosis with impaired physical fitness and cognitive function four-seven years later in a poor urban community in northeast Brazil. *Am J Trop Med Hyg,* 61**:** 707-713, 1999.

13. Lorntz, B, Soares, AM, Moore, SR, Pinkerton, R, Gansneder, B, Bovbjerg, VE, Guyatt, H, Lima, AM, Guerrant, RL: Early childhood diarrhea predicts impaired school performance. *Pediatr Infect Dis J,* 25**:** 513-520, 2006.

14. Niehaus, MD, Moore, SR, Patrick, PD, Derr, LL, Lorntz, B, Lima, AA, Guerrant, RL: Early childhood diarrhea is associated with diminished cognitive function 4 to 7 years later in children in a northeast Brazilian shantytown. *Am J Trop Med Hyg,* 66**:** 590-593, 2002.

15. Patrick, PD, Oriá, RB, Madhavan, V, Pinkerton, RC, Lorntz, B, Lima, AA, Guerrant, RL: Limitations in verbal fluency following heavy burdens of early childhood diarrhea in Brazilian shantytown children. *Child Neuropsychol,* 11**:** 233-244, 2005.

16. Hiscock, H, Scalzo, K, Canterford, L, Wake, M: Sleep duration and body mass index in 0-7-year olds. *Archives of Disease in Childhood,* 96**:** 735-739, 2011.

17. Nokes, C, Grantham-McGregor, SM, Sawyer, AW, Cooper, ES, Bundy, DA: Parasitic helminth infection and cognitive function in school children. *Proc Biol Sci,* 247**:** 77-81, 1992.

18. Nokes, C, McGarvey, ST, Shiue, L, Wu, G, Wu, H, Bundy, DA, Olds, GR: Evidence for an improvement in cognitive function following treatment of Schistosoma japonicum infection in Chinese primary schoolchildren. *Am J Trop Med Hyg,* 60**:** 556-565, 1999.

19. Le Floc'h, N, Otten, W, Merlot, E: Tryptophan metabolism, from nutrition to potential therapeutic applications. *Amino Acids,* 41**:** 1195-1205, 2011.

20. Perlot, T, Penninger, JM: ACE2 - from the renin-angiotensin system to gut microbiota and malnutrition. *Microbes Infect,* 15**:** 866-873, 2013.

21. Mayneris-Perxaches, J, Lima, AA, Guerrant, RL, Leite, AM, Moura, AF, Lima, NL, Soares, AM, Havt, A, Moore, SR, Pinkerton, R, Swann, JR: Urinary N-methylnicotinamide and beta-aminoisobutyric acid predict catch-up growth in undernourished Brazilian children. *Scientific Reports,* in press, 2016.

22. Zelante, T, Iannitti, RG, Cunha, C, De Luca, A, Giovannini, G, Pieraccini, G, Zecchi, R, D'Angelo, C, Massi-Benedetti, C, Fallarino, F, Carvalho, A, Puccetti, P, Romani, L: Tryptophan catabolites from microbiota engage aryl hydrocarbon receptor and balance mucosal reactivity via interleukin-22. *Immunity,* 39**:** 372-385, 2013.

23. Favre, D, Mold, J, Hunt, PW, Kanwar, B, Loke, P, Seu, L, Barbour, JD, Lowe, MM, Jayawardene, A, Aweeka, F, Huang, Y, Douek, DC, Brenchley, JM, Martin, JN, Hecht, FM, Deeks, SG, McCune, JM: Tryptophan catabolism by indoleamine 2,3-dioxygenase 1 alters the balance of TH17 to regulatory T cells in HIV disease. *Sci Transl Med,* 2**:** 32ra36, 2010.

24. Mayneris-Perxachs, J, Lima, AA, Guerrant, RL, Leite, Á, Moura, AF, Lima, NL, Soares, AM, Havt, A, Moore, SR, Pinkerton, R, Swann, JR: Urinary N-methylnicotinamide and β-aminoisobutyric acid predict catch-up growth in undernourished Brazilian children. *Sci Rep,* 6**:** 19780, 2016.

25. ScientificCommitteeOnFood: *Opinion of the Scientific Committee on Food on the Tolerable Upper Intake Levels of Nicotinic Acid and Nicotinamide (Niacin),* Brussels, Belgium, European Commission, 2002.

26. US_National_Library_of_Medicine: Niacin and niacinamide (vitamin B3). <https://www.nlm.nih.gov/medlineplus/druginfo/natural/924.html>, 2016.

27. Knip, M, Douek, IF, Moore, WP, Gillmor, HA, McLean, AE, Bingley, PJ, Gale, EA, Group, ENDIT: Safety of high-dose nicotinamide: a review. *Diabetologia,* 43**:** 1337-1345, 2000.

28. Vague, P, Vialettes, B, Lassmann-Vague, V, Vallo, JJ: Nicotinamide may extend remission phase in insulin-dependent diabetes. *Lancet,* 1**:** 619-620, 1987.

29. Gale, EA, Bingley, PJ, Emmett, CL, Collier, T, Group, ENDITE: European Nicotinamide Diabetes Intervention Trial (ENDIT): a randomised controlled trial of intervention before the onset of type 1 diabetes. *Lancet,* 363**:** 925-931, 2004.

30. Figge, HL, Figge, J, Souney, PF, Sacks, FM, Shargel, L, Janosik, JE, Kaul, AF: Comparison of excretion of nicotinuric acid after ingestion of two controlled release nicotinic acid preparations in man. *J Clin Pharmacol,* 28**:** 1136-1140, 1988.

31. Stern, RH, Freeman, D, Spence, JD: Differences in metabolism of time-release and unmodified nicotinic acid: explanation of the differences in hypolipidemic action? *Metabolism,* 41**:** 879-881, 1992.

32. Allen, LH, Hampel, D, Shahab-Ferdows, S, York, ER, Adair, LS, Flax, VL, Tegha, G, Chasela, CS, Kamwendo, D, Jamieson, DJ, Bentley, ME: Antiretroviral therapy provided to HIV-infected Malawian women in a randomized trial diminishes the positive effects of lipid-based nutrient supplements on breast-milk B vitamins. *Am J Clin Nutr,* 102**:** 1468-1474, 2015.

33. National_Academies_of_Science: Dietary Reference Intakes: Vitamins. <http://www.nationalacademies.org/hmd/~/media/Files/Activity%20Files/Nutrition/DRIs/DRI_Vitamins.pdf>.

34. Morgenthaler, TI, Owens, J, Alessi, C, Boehlecke, B, Brown, TM, Coleman, J, Jr., Friedman, L, Kapur, VK, Lee-Chiong, T, Pancer, J, Swick, TJ: Practice parameters for behavioral treatment of bedtime problems and night wakings in infants and young children - An American Academy of Sleep Medicine report. *Sleep,* 29**:** 1277-1281, 2006.

35. Amza, A, Kadri, B, Nassirou, B, Stoller, NE, Yu, SN, Zhou, Z, West, SK, Mabey, DC, Bailey, RL, Keenan, JD, Porco, TC, Lietman, TM, Gaynor, BD: A cluster-randomized controlled trial evaluating the effects of mass azithromycin treatment on growth and nutrition in Niger. *Am J Trop Med Hyg,* 88**:** 138-143, 2013.

36. Checkley, W, White, AC, Jaganath, D, Arrowood, MJ, Chalmers, RM, Chen, XM, Fayer, R, Griffiths, JK, Guerrant, RL, Hedstrom, L, Huston, CD, Kotloff, KL, Kang, G, Mead, JR, Miller, M, Petri, WA, Priest, JW, Roos, DS, Striepen, B, Thompson, RC, Ward, HD, Van Voorhis, WA, Xiao, L, Zhu, G, Houpt, ER: A review of the global burden, novel diagnostics, therapeutics, and vaccine targets for cryptosporidium. *Lancet Infect Dis,* 15**:** 85-94, 2015.

37. Amadi, B, Mwiya, M, Musuku, J, Watuka, A, Sianongo, S, Ayoub, A, Kelly, P: Effect of nitazoxanide on morbidity and mortality in Zambian children with cryptosporidiosis: a randomised controlled trial. *Lancet,* 360**:** 1375-1380, 2002.

38. Houpt, E, Gratz, J, Kosek, M, Zaidi, AK, Qureshi, S, Kang, G, Babji, S, Mason, C, Bodhidatta, L, Samie, A, Bessong, P, Barrett, L, Lima, A, Havt, A, Haque, R, Mondal, D, Taniuchi, M, Stroup, S, McGrath, M, Lang, D, Investigators, M-EN: Microbiologic methods utilized in the MAL-ED cohort study. *Clin Infect Dis,* 59 Suppl 4**:** S225-232, 2014.

39. Platts-Mills, JA, McCormick, BJ, Kosek, M, Pan, WK, Checkley, W, Houpt, ER, Investigators, M-EN: Methods of analysis of enteropathogen infection in the MAL-ED Cohort Study. *Clin Infect Dis,* 59 Suppl 4**:** S233-238, 2014.

40. Gladstone, M, Lancaster, GA, Umar, E, Nyirenda, M, Kayira, E, van den Broek, NR, Smyth, RL: The Malawi Developmental Assessment Tool (MDAT): the creation, validation, and reliability of a tool to assess child development in rural African settings. *PLoS Med,* 7**:** e1000273, 2010.

41. Donowitz, JR, Haque, R, Kirkpatrick, BD, Alam, M, Lu, M, Kabir, M, Kakon, SH, Islam, BZ, Afreen, S, Musa, A, Khan, SS, Colgate, ER, Carmolli, MP, Ma, JZ, Petri, WA: Small Intestine Bacterial Overgrowth and Environmental Enteropathy in Bangladeshi Children. *MBio,* 7**:** e02102-02115, 2016.

42. Yusuf, S, Lonn, E, Pais, P, Bosch, J, López-Jaramillo, P, Zhu, J, Xavier, D, Avezum, A, Leiter, LA, Piegas, LS, Parkhomenko, A, Keltai, M, Keltai, K, Sliwa, K, Chazova, I, Peters, RJ, Held, C, Yusoff, K, Lewis, BS, Jansky, P, Khunti, K, Toff, WD, Reid, CM, Varigos, J, Accini, JL, McKelvie, R, Pogue, J, Jung, H, Liu, L, Diaz, R, Dans, A, Dagenais, G, Investigators, H-: Blood-Pressure and Cholesterol Lowering in Persons without Cardiovascular Disease. *N Engl J Med,* 374**:** 2032-2043, 2016.

43. Locks, LM, Manji, KP, McDonald, CM, Kupka, R, Kisenge, R, Aboud, S, Wang, M, Fawzi, WW, Duggan, CP: Effect of zinc and multivitamin supplementation on the growth of Tanzanian children aged 6-84 wk: a randomized, placebo-controlled, double-blind trial. *Am J Clin Nutr,* 103**:** 910-918, 2016.

44. Soofi, S, Cousens, S, Iqbal, SP, Akhund, T, Khan, J, Ahmed, I, Zaidi, AK, Bhutta, ZA: Effect of provision of daily zinc and iron with several micronutrients on growth and morbidity among young children in Pakistan: a cluster-randomised trial. *Lancet,* 382**:** 29-40, 2013.

45. WHO: Haemoglobin concentrations for the diagnosis of anaemia and assessment of severity. . *Vitamin and Mineral Nutrition Information System*Geneva, World Health Organization 2011.

46. Prado, EL, Dewey, KG: Nutrition and brain development in early life. *Nutr Rev,* 72**:** 267-284, 2014.
