# Supplementary material for: Effect of scheduled antimicrobial and nicotinamide treatment on linear growth in children in rural Tanzania: A factorial randomized, double-blind, placebo-controlled trial
Source: PLoS Med. 2021 Sep 28;18(9):e1003617. doi: 10.1371/journal.pmed.1003617 (PMC8478246; doi:10.1371/journal.pmed.1003617)
Supplement: S1 Text — NIMR, National Institute for Medical Research. (PDF) [file pmed.1003617.s003.pdf]

Haydom Lutheran Hospital
ELCT Mbulu Diocese

IRB-HSR#19465: ELICIT: Early **L**ife **I**nterventions for **C**hildhood growth and development **I**n **T**anzania

Date: May 08, 2019

Ref. HLH/MBL/N.01/14

The NatHREC Secretariat, National Institute for Medical Research,
C/O Prof. Yunus Mgaya, Director General,
National Institute for Medical Research,
POB 9653, Dar es Salaam,
Tanzania

Dear National Health Research Ethics Committee

RE: Responses to suspension of a clinical trial titled" Early Life Interventions for Childhood Growth and Development in Tanzania"(ELICIT)

This letter is in response to your letter with reference NIMR/HQ/R.8a/Vol.1/2019 dated 25th April 2019 (which we received 4th May 2019) notifying the Principal Investigator and the Study Team of the suspension of the clinical trial research study we are conducting entitled, "Early Life Interventions for Childhood Growth and Development in Tanzania (ELICIT)." a collaborative research project between Haydom Global Health Research Centre (HGHRC) at Haydom Lutheran Hospital (HLH), National Institute for Medical Research (NIMR) Haydom Station, through NIMR Muhimbili and the University of Virginia, US.
We were very surprised and humbled by this decision. We appreciate the oversight and assistance NatHREC provides.
Please accept our responses to the queries raised, which are addressed in a point-by-point fashion in the attached responses.

Together with this letter, we accompany the following documents which supports our responses:

1. ELICIT Protocol v 5.0.
2. The NIMR approved MTA between HLH and the University of Maryland
3. The Memorandum of Understanding (MoU) for lab testing between HLH and Kilimanjaro Clinical Research Centre
4. Hand washing SOP for HGHRC.
5. Interim analysis report
6. Data Management SOP for HGHRC
7. "Note to file" notification related to signing and stamping CRFs after data entry.

Sincerely

Estomih Mduma,
ELICIT clinical trial research study PI,
Haydom Global Health Research Centre,
Haydom Lutheran Hospital.
Mbulu, Manyara

Haydom Lutheran Hospital
ELCT Mbulu Diocese

IRB-HSR#19465: ELICIT: Early **L**ife **I**nterventions for **C**hildhood growth and development **I**n **T**anzania

RE: Responses to suspension of a clinical trial titled" Early Life Interventions for Childhood Growth and Development in Tanzania"(ELICIT)

1. There were three points of critique stated under point 1; we will address these individually:
   a. **All samples were collected from participants and stored at -80 degrees until testing.**

      RESPONSE: Stool testing aimed for antimicrobial-related outcomes largely toward the end of the study. We now see that the wording in our protocol was unclear, and we apologize for the initial lack of clarity. Our protocol ((Protocol Version 5.0 (attached) on page 17, as is referenced in the Visit Report)) addresses laboratory testing of stool samples in point 2.a.:

      Point i. states the timing of collection ("Stool will be collected monthly and evaluated for pathogens and microbiota at months 3, 6, 6+14 days, 9, 12, 12+14 days, 15 and 18 and yearly thereafter")
      Point ii. states the immediate handling of samples ("Samples will be batched and stored at -80 degrees")
      Point iii. describes the various tests involved ("testing will include Taqman array cards, culture and microbiota analysis").

      We now appreciate how the reviewer may have interpreted point i. to suggest that the evaluation of stool was performed immediately upon collection. A further explanation; the testings are not being performed immediately because of the labor-intensive the testing procedures. Again some of these samples are being tested in Haydom (using Taqman Array Cards, TAC), while other tests will be performed at the University of Maryland (approved MTA attached). The results from these stool tests are for research purposes only and the data obtained will be analyzed at the end of the study in the context of other factors (e.g. final anthropometry measures, intervention sub-group, etc.). (The same is true for other types of testing, with point 1.f.iii [at the top of page 17], the protocol states, "Serum will be batched and stored at -80 until time of testing.")

      If it is desirable by the Committee, we will clarify this point further by revising the wording of the protocol point 2.a.i. to state: "Stool will be collected at months 3, 6, 6+14 days, 9, 12, 12+14 days, 15 and 18 and yearly thereafter, with testing performed when possible."

   b. **Some stool samples were taken to KCRI for testing; however, there was no approval for Material Transfer Agreement issued by NatHREC.**

      RESPONSE: We deeply apologize for this. Our understanding was that the requirement for MTA was related to samples that are being shipped oversee (out of the country), as documented in the MRCC _form2 "item number 9" which state that "… Please note that if samples are to be shipped outside Tanzania MTA clearance is required." We had shipped some stool to KCRI (in Moshi, TZ) because of the large amount of work needed to test more than 7000 stool samples. The lack of contract/MoU was pointed out by the TFDA during their last year inspection (2-3 July 2018), and we have established a MoU between Haydom Lutheran Hospital and Kilimanjaro Clinical Research Centre (attached). However, that lack of MTA was also pointed out by TFDA, who performed a site visit at the same time as the NatHREC visit. In response to the TFDA comments, we suspended shipment of stool samples to KCRI and we are applying NIMR for MTA to transfer stool to KCRI;

this solution was accepted by the TFDA. Shipment of stool samples to KCRI will only continue after getting MTA approval.

c. **PI is absent from the Research site: He is doing PhD study in Norway without notifying the NatHREC secretariat.**

RESPONSE: We agree with the Monitor observation on the absence of the PI during the visit. We again apologize for this as it was a coincidence that the monitoring visit coincide with the PI travel and it was difficult to postpone as was a long-time planned travel. We tried to ask a favor for the TFDA reschedule their visit but was not possible. The absence of the PI during the GCP inspection visit was communicated with TFDA team prior to the visit and arrangements were made to all sections/departments of the study to ensure the inspection visit is successful even in the absence of the PI.

PhD: The PI started work toward the PhD in 2012. This is not a full-time course, and most of the PhD-related activities take place in Haydom and consume minimum time (about 30% which is out of the time effort required to oversee the trial). Additionally, the PI occasionally travels for one or two weeks for the purpose related to the study and his work on his PhD. The PI is continuously in contact with the study team while away from Haydom, via phone and emails. In the PI's absence, the chain of command for the next responsible person in the study is Samwel Jatosh (Research Coordinator), Paschal Mdoe MD (Investigator), Joshua Gideon MD (Investigator), and Justine Museveni MD (Investigator). In rare cases when one of these is away, it is communicated to the next in this line and to the PI who is in charge on site. The PI maintains contact with the person in charge to receive updates on a daily basis and to help problem solve issues that arise as needed.

We apologize for not alerting the NatHREC secretariat of this arrangement as we were not informed that the TFDA inspectors were coming together with the NatHREC member, and in the future, as directed, we will notify NatHREC in the event of prolonged travel out of country.

2. **Stool samples were not tested as specified in the protocol (i.e. at months 3, 6, 6+14 days, 9, 12, 12+14 days, 15, 18 and yearly); instead all samples were achieved in the freezer at -80 degree which is contrary to the approved protocol version 5.**

   RESPONSE: We again apologize for any lack of clarity in this description. Please see point 1a above.

3. **SOP for handwashing was not found in the microbiology lab.**

   RESPONSE: This point was also identified by the TFDA during their visit that took place at the same time as the NIMR visit. Since that time, the laboratory has developed and implemented a handwashing SOP. Please refer to the attached SOP that is posted in our facility.

4. **Some stool samples were taken to KCRI for testing; however, there was no approval for Material Transfer Agreement issued by NatHREC.**

RESPONSE: We apologize for this. This point is further addressed under 1.b. above.

(Please note, points of critique for numbers 5 to 8 were not listed in the letter; the report proceeded from point number 4 directly to point number 9, to which we reply below.)

9. **There were 237 SAEs reported of which majority were Gastroenteritis and Anaemia according to the DSMB report of the meeting held on 9th August, 2018 the same was discussed and PI instructed to conduct ad hoc analysis of the samples collected for the participants in both study groups (Intervention and Control arms) by December, 2018. The aim was to investigate if there will be significance difference of the results between the two groups in order for the DSMB to decide whether to un-blind or continue blinding the study.**
   RESPONSE: Our interim analysis came about from two influences:
   1. As a study team, we had been following for differences in AE's and SAE's by nicotinamide intervention group (Nic-A vs. Nic-B, where we are blinded which group is the active drug and which is the placebo) and wished to investigate the potential that one study arm had more diarrhea AE's than the other.
   2) The Bill & Melinda Gates Foundation (the funder of the study) was interested in whether there were potential differences in anthropometry measures between intervention groups at the 6-month point in the study.

   The Gates Foundation had requested that we perform an interim analysis to assess these 6-month outcomes and had initially identified mid-December as a date for this. When we had our DSMB meeting on 9 October (we did not have a DSMB meeting in August, so this must have been the DSMB meeting in question), we informed the DSMB about our concerns about the possibility of differential AE's between nicotinamide intervention groups, and said that we would perform a more formal analysis of this when we did the outcomes testing in December. However, the Gates Foundation then requested that we perform this analysis later to allow a larger number of participants to reach 6 months; we informed the DSMB chair of the change in timing on 18 December 2018.
   We performed the interim analysis on 25 January 2019 and informed the DSMB of the results on 28 February. These results showed no difference between nicotinamide intervention group for AE's or SAE's, either all together or AE's and SAE's specific to gastroenteritis or pneumonia. This was acceptable to the DSMB (please see attached report for interim analysis), and no un-blinding was requested. We have also determined that there was no difference in SAE anemia by nicotinamide group.
10. **Many protocol deviations which related to not adhering to the administration of IMP schedule were observed.**
   RESPONSE: We have carefully re-reviewed our data regarding this. Out of 9840 drug administration forms, we found 70 that were given outside the planned time window. While our goal is 0%, the doses outside the timing window represent less than 1% of doses of study medications—and this error rate has decreased over time, with only 6 incidents happening since 1 January 2019. To further improve this rate, we will hold a re-training session with the study team on the anticipation and adherence to the medication time windows.

(Please note, we did not receive any indication of a point of critique numbered 11; the report proceeded from point number 10 to point number 12.)

12. **CRF's in the several participants files reviewed example PID TZ1C1031, TZ1C1032, TZ1C1041, TZ1C1191, TZ1C1192, TZ1C1162, TZ1C1222, TZ1C1172, TZ1C1151, TZ1C1052, TZ1C1071, TZ1C1072, TZ1C1142, TZ1C1011, Including Child monthly form, drug administration form, specimen collection form, pill/pack counting form,**

breast milk collection form were not signed and dated by first and second data entry personnel and some were signed by first of second data entry personnel only.

RESPONSE: We apologize for these omissions of signatures. To clarify more on this matter, this was also raised during the FHI trial-monitoring visit (shared with NIMR in the past), which took place on the 12-16 March 2018. The site amended the data management SOP (attached) and implemented the procedure to "sign and stamp" all the CRFs after data entry (both 1st and 2nd entry) as from 3rd April 2018, and a note to file was completed to cover the CRFs before the date and stated the reason to not have signature and stamp. What was now observed in the resent inspection (on this discussion), 02 out of the 14 PIDs are involved and the rest (12) were before implementation of the SOP. However we will also hold a re-training session with field workers and data entry personnel on the importance of signing forms upon completion of data entry, to avoid such omissions in the future.

Conclusion: We are grateful for the NatHREC's review of our study and are eager to respond to any further points of critique the committee might have. The ELICIT study aims to reveal important relationships between intervention with antimicrobials and nicotinamide and child thriving (growth and cognitive development) in a resource-limited setting. We are hopeful that NatHREC will permit us to complete this study as planned. Please do not hesitate to contact us with any future questions. Finally, as the PI of this project, I will be happy to appear before the NatHREC Clinical trial subcommittee for more conversations as early as possible for their convenience.

NATIONAL INSTITUTE FOR MEDICAL RESEARCH
HEADQUARTERS

Telephone: +255-22-2121400
Telefax: +255-22-2121360
E-mail: hq@nimr.or.tz
Website: www.nimr.or.tz

3 Barack Obama Drive
P.O. Box 9653
11101 Dar es Salaam
Tanzania

Our Ref: NIMR/HQ/R.8a/Vol.II

4th June 2019

Dr. Estomih Mduma
Haydom Global Health Research Center
Haydom Lutheran Hospital
P.O. Box 9000, Mbulu
Manyara

RE: PERMISSION FOR RESUMPTION OF A CLINICAL TRIAL TITLED "EARLY CHILD INTERVENTIONS FOR CHILDHOOD GROWTH AND DEVELOPMENT IN TANZANIA (ELICIT)"

Reference is made to the caption above.

On the 25th April 2019, I approved a letter to suspend the ELICIT trial. Following your responses to MRCC directives in a cover letter dated 8th May 2019, NatHREC invited you to attend its 37th Clinical Trials Sub-committee meeting which was held on 16th May 2019 for further dialogue. The outcome of the dialogue was extended to the 119th NatHREC meeting held on 30th May 2019 for final decision. I am pleased to inform you that NatHREC forwarded satisfactory resolution for ELICIT trial to MRCC in order to approve resumption of the ELICIT trial. The Medical Research Coordinating Committee (MRCC) directs you to amend the ELICIT trial protocol according to the changes discussed during the 37th Clinical Trials Sub-committee meeting. Attached is a report (Appendix 5) on the dialogue during the 37th Clinical Trials Sub-committee meeting for your information.

This letter serves as a permit for you to resume the trial and kindly be informed that, MRCC will keep monitoring the progress of the trial to ensure safety and security of the participants of the trial.

Yours sincerely,

Prof. Yunus D. Mgaya
DIRECTOR GENERAL

Cc: Managing Medical Director, Haydom Lutheran Hospital
Cc: ELICIT Project DSMB
Cc: NatHREC
Cc: TFDA

APPENDIX 5

28th May 2019

REPORT ON DISCUSION AND RECOMMENDATION TO LIFT SUSPENSSION OF A CLINICAL TRIAL TITLED "EARLY CHILD INTERVENTIONS FOR CHILDHOOD GROWTH AND DEVELOPMENT IN TANZANIA (ELICIT)"

The Clinical Trials (CT) Sub-committee met and discussed with the Principal Investigator (PI) of the captioned clinical trial on various protocol and Standard Operating Procedure violations reported by the NatHREC Inspectors after the oversight visit done from 18 to 19 February 2019. The report led the MRCC to suspend the project pending reception of a response letter and appearance of the PI before the CT sub-committee. The PI submitted a written response to various charges within the given time, and then he was invited to appear before the CT Sub-Committee 37th meeting scheduled for 16th May 2019 at NIMR HQ. The main objective of summoning the PI was to further discuss and interview him on the issues reported by the inspectors. The CT Sub-Committee meeting was successful held on 16th May 2019, and Dr Mduma (the PI) accompanied by the Project Co-investigator Dr. Sokoine Kivuyo from NIMR Muhimbili attended the meeting. The CT Sub-committee interviewed them based on the queries in the MRCC letter of suspension as described below:

1. <u>Samples were collected from participants and stored at -80 degrees Celsius until testing.</u>
   The PI apologised for the confusion and informed the Committee that the protocol was not clear under this section, because the same section had a sub-section which provisioned for storage until testing. The PI agreed to submit an amendment which shall clarify this issue in the respective section and sub-section. In principle, the CT Sub-committee agreed that this was a protocol deviation and the PI should in deed submit an amended protocol.

2. <u>Stool samples were taken to Kilimanjaro Clinical Research Institute (KCRI) for testing but this was not what is specifically described in the protocol. This raised two concerns as follows;</u>

a. The approved protocol was not followed as required thus leading to protocol deviation. The PI agreed that this was an oversight, and he stated that they were under the impression that processing samples in the country would not require Materials Transfer Agreement (MTA), hence for capacity building they had agreed to share samples with KCRI.

   The CT Sub-Committee instructed the PI to amend the protocol to accommodate this arrangement, and sign the MoU with KCRI with contacts of the responsible person stated in the amended protocol.

b. Prof. Blandina Mmbaga is the person responsible for the samples at KCRI, also an investigator as well a member of the Data and Study Monitoring Board (DSMB). The Committee was concerned with a potential conflict of interest. As a result, the PI thought of retracting the idea of sending samples to KCRI and perform the experiments at Haydom.

3. The PI was absent on the date the site visit was conducted by TFDA and NatHREC member. He was reported to be doing his PhD, which is not part of this study.

   The PI reported to the Committee that he is able to commit time to the study, about 30-50% of the time, he has currently finished doing his PhD research and is at dissemination stage. He reported that on average he might be travelling less than 3 times in a year and the longest duration he was away from site was less than 2 weeks.

4. Missing handwashing SOP in the Microbiology laboratory. The PI apologised for this oversight and informed that the SOP has already been placed in the Microbiology laboratory

5. There were many Serious Adverse Events (SAEs) reported from site mostly gastrointestinal and anaemia. The DSMB had noted this and asked the study PI to do an interim analysis and compare the three treatment groups. In fact, the study PI acknowledged that investigators

and the sponsor (Gates Foundation) were the first people to raise concern regarding high number of reported SAEs. This concern lead to the need of an interim analysis that was agreed with the DSMB. Later the DSMB requested that the study should delay the interim analysis until December 2018 in order to have a good sample size. The interim analysis was eventually conducted in March 2019, and the results showed no significant differences between the three treatment groups in SAEs.

6. Protocol deviations on Investigational Medicinal Product (IMP) administration. The PI informed that only 1% of the drug administration forms reported drugs administered outside the schedule, and explained that though it was the intention of the PI to make sure patients take drugs within the specified time, but this was sometimes impossible as patients were given drugs to take home. PI further explained that staff were retrained in order to improve on performance. The Committee agreed that this amounted to protocol deviation.

7. Case Report Forms (CRFs) from several participants were not counter signed. The PI apologised for this and informed that in response to this, the site amended the data management SOP and a note to file was put on the files to cover the dates that the CRFs were not counter signed. The Committee noted this and commented that this implied poor monitoring of the site by the PI.

8. Request from Dr. Mduma. After the interview, Dr Mduma requested the Clinical Trials Sub-committee members grant him permission if possible to continue with data collection while waiting for the final decision from NatHREC. The reason for the request was that, the trial participants are now completing month 18, the period that they take measurements and information (trial endpoint), which will be used to answer the research questions. This is because, if they wait for letter from MRCC lifting the suspension, it might not come in time and thus becoming impossible to get data from participants who will be above 18 months of age. The CT Sub-committee insisted that only the full Committee (NatHREC) can

make decision to retract the suspension after being satisfied with the responses and Good Clinical Practice (GCP) is followed and participants are going to be safe.

9. Decision to lift the suspension of the ELICIT Trial. After reviewing the PI's responses during the 37th Clinical Trials Sub-committee meeting, members were satisfied with the responses and recommended lifting the suspension when the NatHREC meets at the 119th meeting on 30th May 2019. Then the NatHREC will write to inform the MRCC on the responses from the PI which are satisfactory.

End of the report

Dr. Paul E. Kazyoba
NatHREC Secretary
