# Supplementary material for: Effect of scheduled antimicrobial and nicotinamide treatment on linear growth in children in rural Tanzania: A factorial randomized, double-blind, placebo-controlled trial
Source: PLoS Med. 2021 Sep 28;18(9):e1003617. doi: 10.1371/journal.pmed.1003617 (PMC8478246; doi:10.1371/journal.pmed.1003617)
Supplement: S1 Analysis Plan — (DOCX) [file pmed.1003617.s004.docx]

ELICIT Primary Statistical Analysis Plan 5 February 2020

*I. Unblinding, outcomes, analysis populations, data cleaning*

**Timing of analysis plan and unblinding**

This analysis plan is being posted prior to the termination of data collection (anticipated as early as 24 February, 2020). The study will remain blinded until the following conditions have been reached:

1. All 18-month anthropometry, 18-month cognitive testing, and 18-month questionnaires data collection are completed.
2. Database has been cleaned.
3. Primary analysis has been completed (blinded to intervention group), as described below.

Development of analysis code will be conducted on a dataset in which intervention groups have been randomly permuted. The final primary analysis will be conducted on true intervention groups blinded to which group corresponded to which intervention.

**Outcomes**

Primary outcome: Length-for-age z-score (LAZ) at 18 months

Secondary outcomes:

1. Weight-for-age z-score (WAZ) at 18 months
2. Head-circumference-for-age z-score (HCZ) at 18 months
3. Weight-for height z-score (WHZ) at 18 months
4. MUAC-for-age z-score at 18 months
5. Mortality
6. SAE’s, overall and by category
7. AE’s, overall and by category

**Analysis populations**

1. Intention to Treat (ITT): All children who were enrolled in the study and randomized.
2. Modified Intention to Treat (mITT): All children with a valid 18-month anthropometry measurement. The study was suspended by the Tanzanian regulatory agencies for a 9-week period, requiring some assessments to be performed up to 9 weeks outside the target date; these late measurements of the 18-month anthropometry outcomes related to the study suspension will be included.
3. Per-protocol: All children in the mITT group who met the additional criteria:
   1. Breastfed (at least partially) through age 6 months
   2. Received all doses of azithromycin
   3. Received initial dose of each nitazoxanide course
   4. Received no more than one drug administration (at 6, 9, 12, and 15 month visits) outside of the 14-day window around the target date
   5. Received at least 50% of nicotinamide doses as measured by pill and sachet counting

**Cleaning of anthropometry z-score data**

Anthropometry z-scores were calculated using the zscorer v0.2 package based on the WHO child growth standards. Growth rates between each pair of adjacent measurements were then calculated. Any growth rates that were in the bottom 0.5% or the top 99.5% of the distribution for each age category were flagged. Absolute Z score differences between each measurement and its two flanking measurements were then used to determine which of the measurements was implausible. This implausible value was then coded as NA. Maternal heights will be cleaned by invalidation of values more than 6 standard deviations from the mean.

*II. Analysis of Z-score outcomes (LAZ, WAZ, HCZ, WHZ, MUAC) -- mITT and per-protocol groups*

ELICIT is a factorial study assessing two interventions (nicotinamide vs. placebo; antimicrobials vs. placebo). Our a priori plan was to analyze each intervention separately, and the study was powered for each primary effect. Thus, all of the processes described below to compare between intervention groups will first be done for nicotinamide (NIC-A vs. NIC-B) and then for antimicrobials (ANTI-A vs. ANTI-B). This includes looking for baseline differences and choosing adjustment variables separately between NIC-A vs NIC-B and ANTI-A vs ANTI-B.

To determine if there are any synergistic effects of nicotinamide and antimicrobials, we will then include an interaction term between the two interventions in a linear regression model for the 18-month outcomes. If there is a statistically important interaction (defined as p<0.05 from a Wald test), or a sizeable point estimate of the interaction term (defined as a difference of >0.2 z-scores), we will report effects separately for NIC alone vs. control, ANTI alone vs. control, and NIC+ANTI vs. control.

**Unadjusted analysis:** Unadjusted analyses will be conducted by 2-sided t-test with alpha=0.05 and equal variance.

**Adjusted analysis**: We will also conduct an adjusted analysis to improve power and/or account for potential baseline differences as well as differences by loss-to-follow-up. Factors considered for the adjusted analysis include:

1. Enrollment measurement of outcome (e.g. LAZ, WAZ, HCZ, MUACZ)
2. Sex
3. Socioeconomic status (measured by the WAMI^1^)
4. Maternal age, height, tribe, and education
5. Birth month
6. Hospital birth
7. Firstborn status
8. Ward of residence
9. Age when 18-month outcome was assessed

A priori, we will include the baseline measure of the corresponding anthropometry outcome in the adjusted analyses (NB: WAZ will be used for MUAC). Each of the remaining covariates above will be assessed in a bivariate analysis for the association with the 18-month outcome using linear regression. Those covariates with p<0.2 or difference of 0.2 z-scores will be included in a multivariable analysis based on a linear regression model including the intervention group and each of the factors meeting the criteria above. For covariates that can be specified in different ways (e.g. using birth month as opposed to birth season), we will choose the model with the highest AIC. If point estimates and/or inference differ substantially between the unadjusted and adjusted analysis, we will rely on the adjusted analysis for the primary interpretation of results to ensure differences at 18-months are not explained by baseline differences.

For baseline characteristics with less than 5% missing data, we will single impute the mean values to retain all children in the adjusted analysis and retain comparability with the unadjusted analysis. In the adjusted analysis, for characteristics with >5% missing data, we will consider multiple imputation if the covariate meets the criteria for inclusion in the model. We will also investigate whether missing outcome measurements (or lost-to-follow-up before 18 months) are associated with randomization arm or baseline characteristics. If so, we will use inverse-probability of censoring weighting to account for informative censoring.

**Sensitivity analysis for mITT group**: Unadjusted and adjusted sensitivity analyses will be performed in which children who have the 18-month outcome measurement taken outside the pre-specified measurement window will be excluded from the mITT group.

*II. Adverse events, serious adverse events, and mortality – ITT group*

For adverse events and serious adverse events, as well as for specific types of serious adverse events, unadjusted and adjusted incidence rate ratios comparing intervention arms will be calculated using Poisson or negative binomial regression as appropriate, with an offset term for the log of person time at risk.

For the analysis of the effects of antimicrobials, only events and person-time starting at six months (the first scheduled administration of antimicrobials) will be included.

For mortality, unadjusted analysis will be performed using a Kaplan-Meier survival analysis, using a log-rank test to test for differences between intervention arms. The adjusted analysis will be performed using a Cox proportional hazards model.

**References**

1. Psaki SR, Seidman JC, Miller M, Gottlieb M, Bhutta ZA, Ahmed T, et al. Measuring socioeconomic status in multicountry studies: Results from the eight-country mal-ed study. *Population Health Metrics*. 2014;12
