# Supplementary figures and images for: Effect of scheduled antimicrobial and nicotinamide treatment on linear growth in children in rural Tanzania: A factorial randomized, double-blind, placebo-controlled trial
Source: PLoS Med. 2021 Sep 28;18(9):e1003617. doi: 10.1371/journal.pmed.1003617 (PMC8478246; doi:10.1371/journal.pmed.1003617)

**S1 Fig: Factorial design.**

**
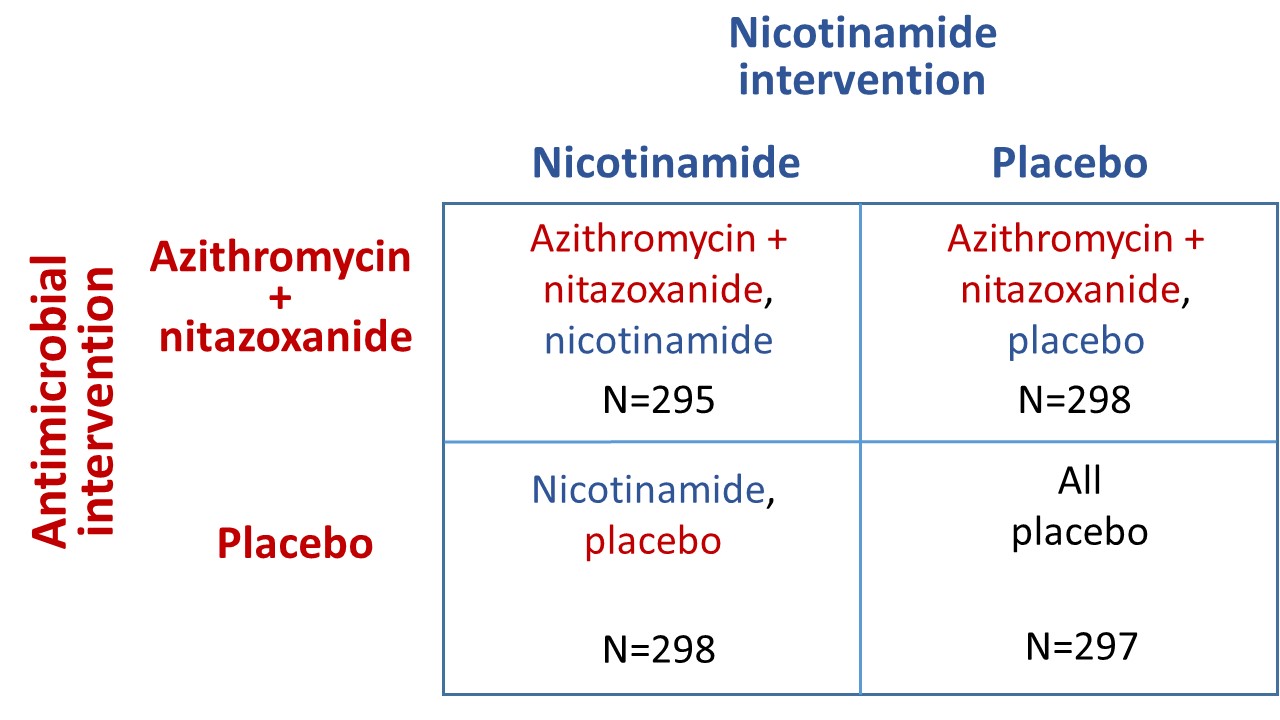
**

Supplement: S1 Fig — (DOCX) [file pmed.1003617.s005.docx]

**S2 Fig: Trial profile by individual intervention**

**
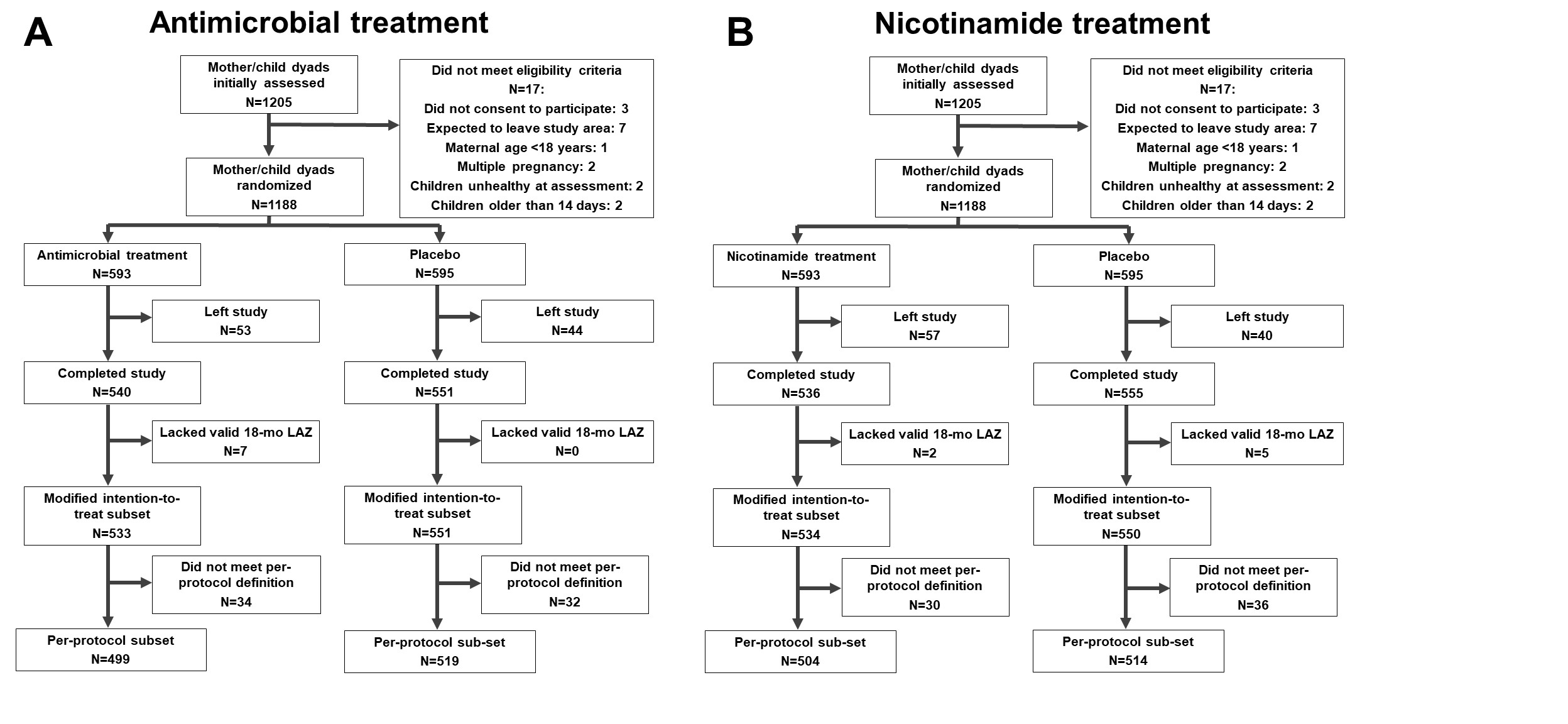
**

Supplement: S2 Fig — (DOCX) [file pmed.1003617.s006.docx]
