# Supplementary material for: Effect of scheduled antimicrobial and nicotinamide treatment on linear growth in children in rural Tanzania: A factorial randomized, double-blind, placebo-controlled trial
Source: PLoS Med. 2021 Sep 28;18(9):e1003617. doi: 10.1371/journal.pmed.1003617 (PMC8478246; doi:10.1371/journal.pmed.1003617)
Supplement: S3 Fig — Subgroup analysis for effect of nicotinamide intervention (A) and antimicrobial intervention (B) on LAZ by SES (WAMI), weight at study entry, sex, and harvest season. LAZ, length-for-age z-score; SES, socioeconomic status; WAMI, improved water and sanitation, assets, maternal education, and household income. (DOCX) [file pmed.1003617.s007.docx]

**
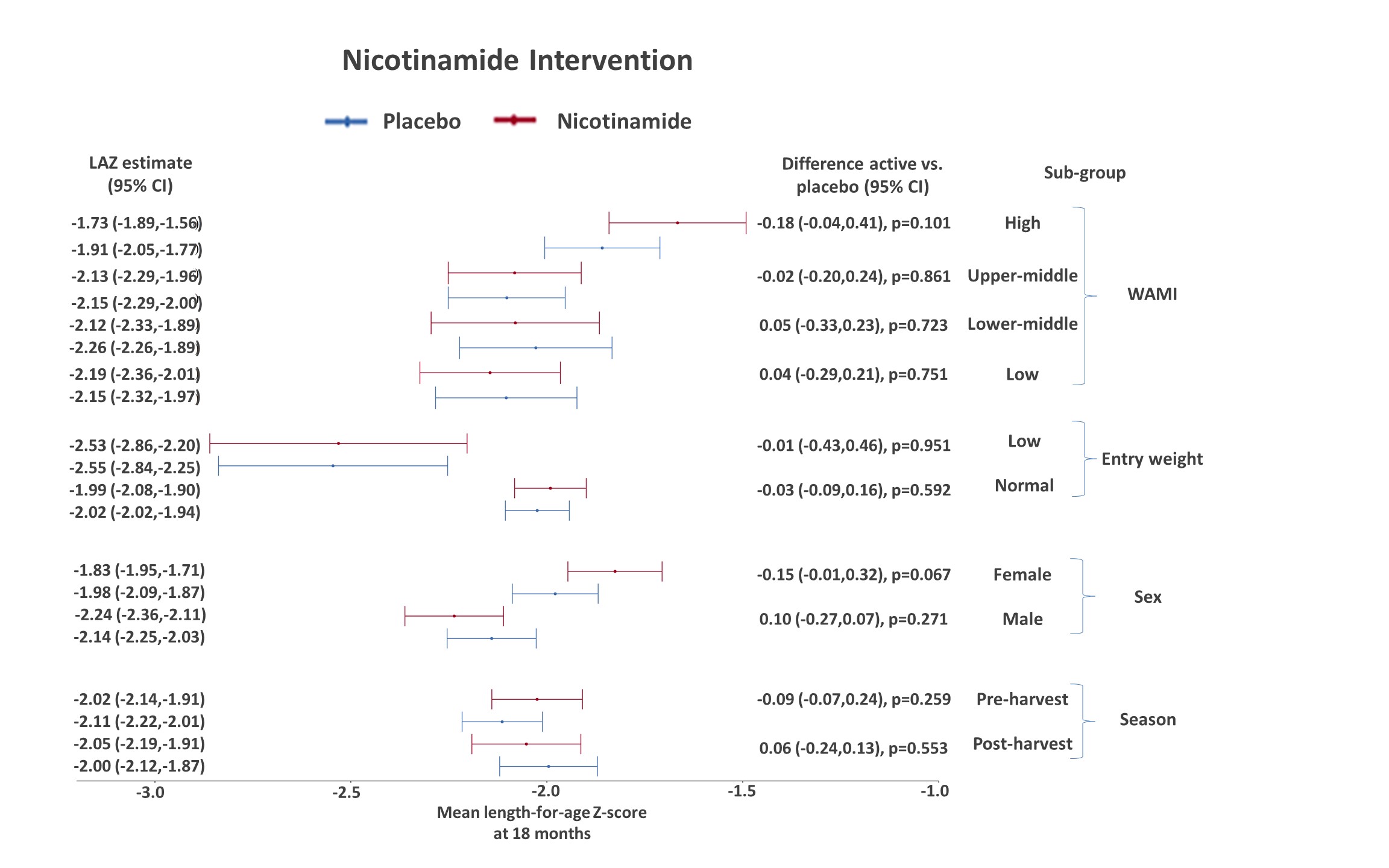
S3 Fig A: Sub-group analyses for effect of nicotinamide intervention on LAZ by SES (WAMI), weight at study entry, sex and harvest season.**


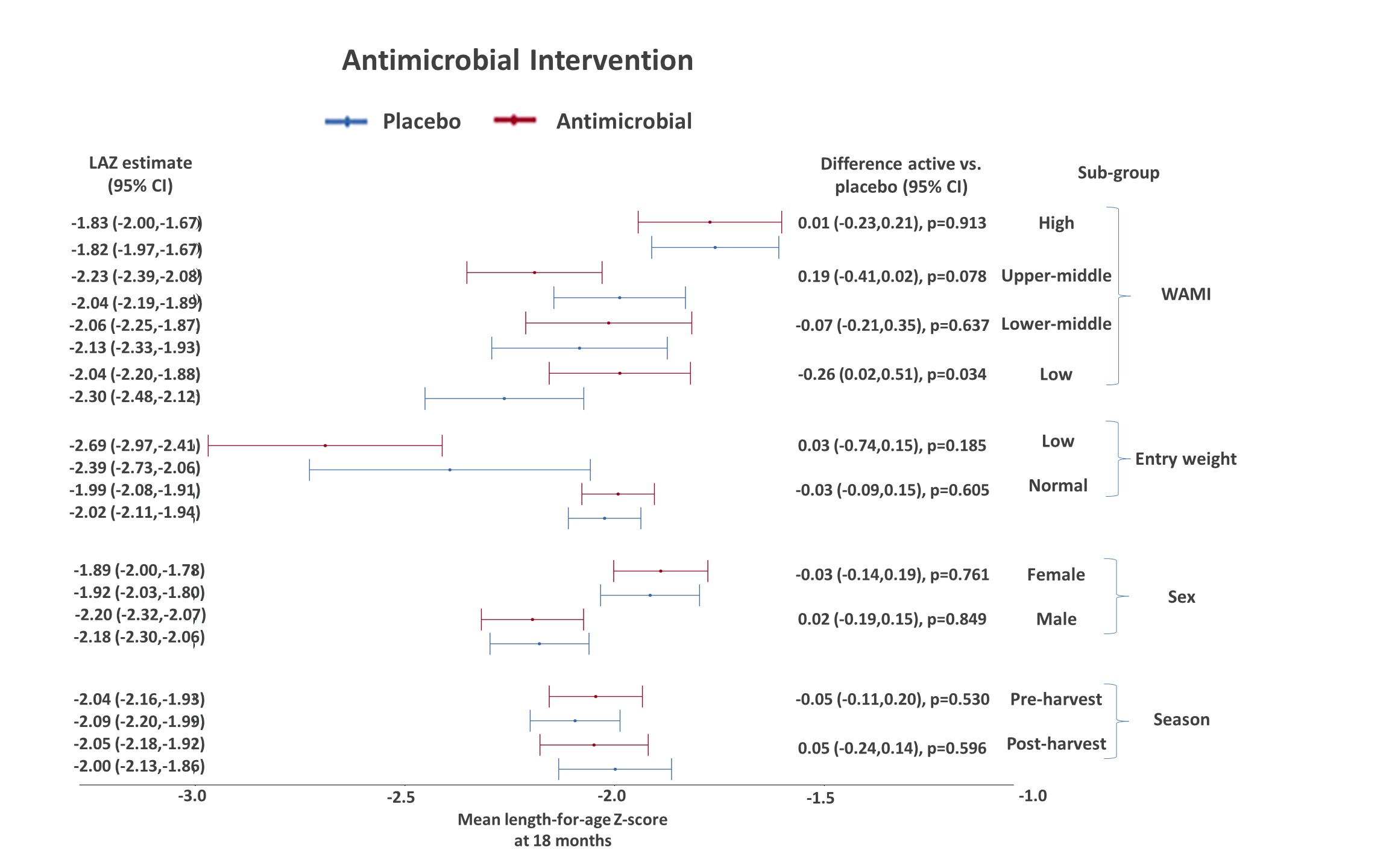
**S3 Fig B: Sub-group analyses for effect of antimicrobial intervention on LAZ by SES (WAMI), weight at study entry, sex and harvest season.**
