# Supplementary material for: Effect of scheduled antimicrobial and nicotinamide treatment on linear growth in children in rural Tanzania: A factorial randomized, double-blind, placebo-controlled trial
Source: PLoS Med. 2021 Sep 28;18(9):e1003617. doi: 10.1371/journal.pmed.1003617 (PMC8478246; doi:10.1371/journal.pmed.1003617)
Supplement: S4 Fig — (DOCX) [file pmed.1003617.s008.docx]

**S4 Fig: Distribution of anthropometry Z-scores at 18 months by intervention group for the modified Intention-to-Treat analysis.**

**
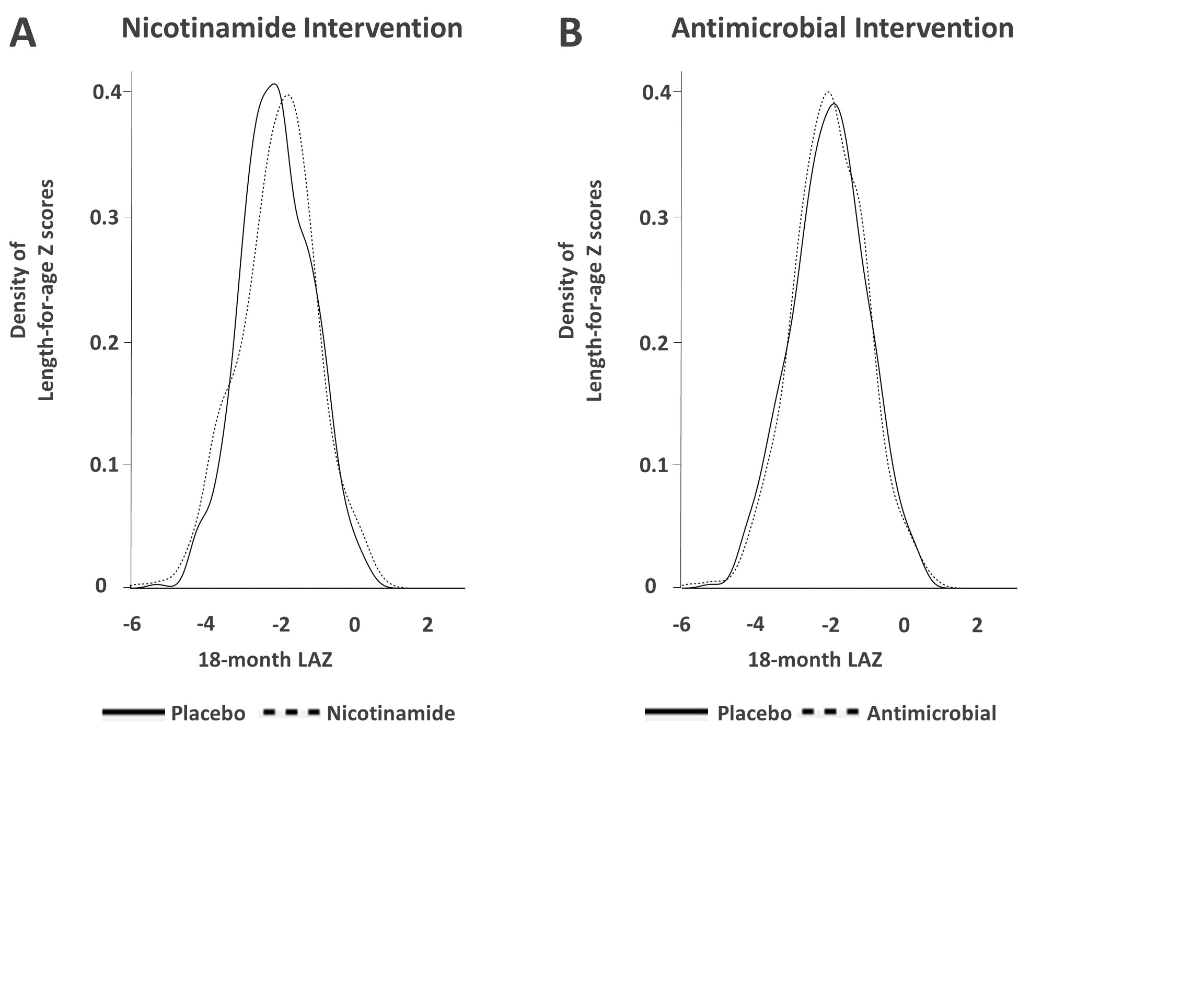
**
