# Supplementary material for: Effect of scheduled antimicrobial and nicotinamide treatment on linear growth in children in rural Tanzania: A factorial randomized, double-blind, placebo-controlled trial
Source: PLoS Med. 2021 Sep 28;18(9):e1003617. doi: 10.1371/journal.pmed.1003617 (PMC8478246; doi:10.1371/journal.pmed.1003617)
Supplement: S5 Fig — Shown are mean values with error bars representing 95% CIs. CI, confidence interval. (DOCX) [file pmed.1003617.s009.docx]

**S5 Fig: Anthropometry Z-scores over time by intervention group for the modified per-protocol analysis.** Shown are mean values with error bars representing 95% confidence intervals.

**
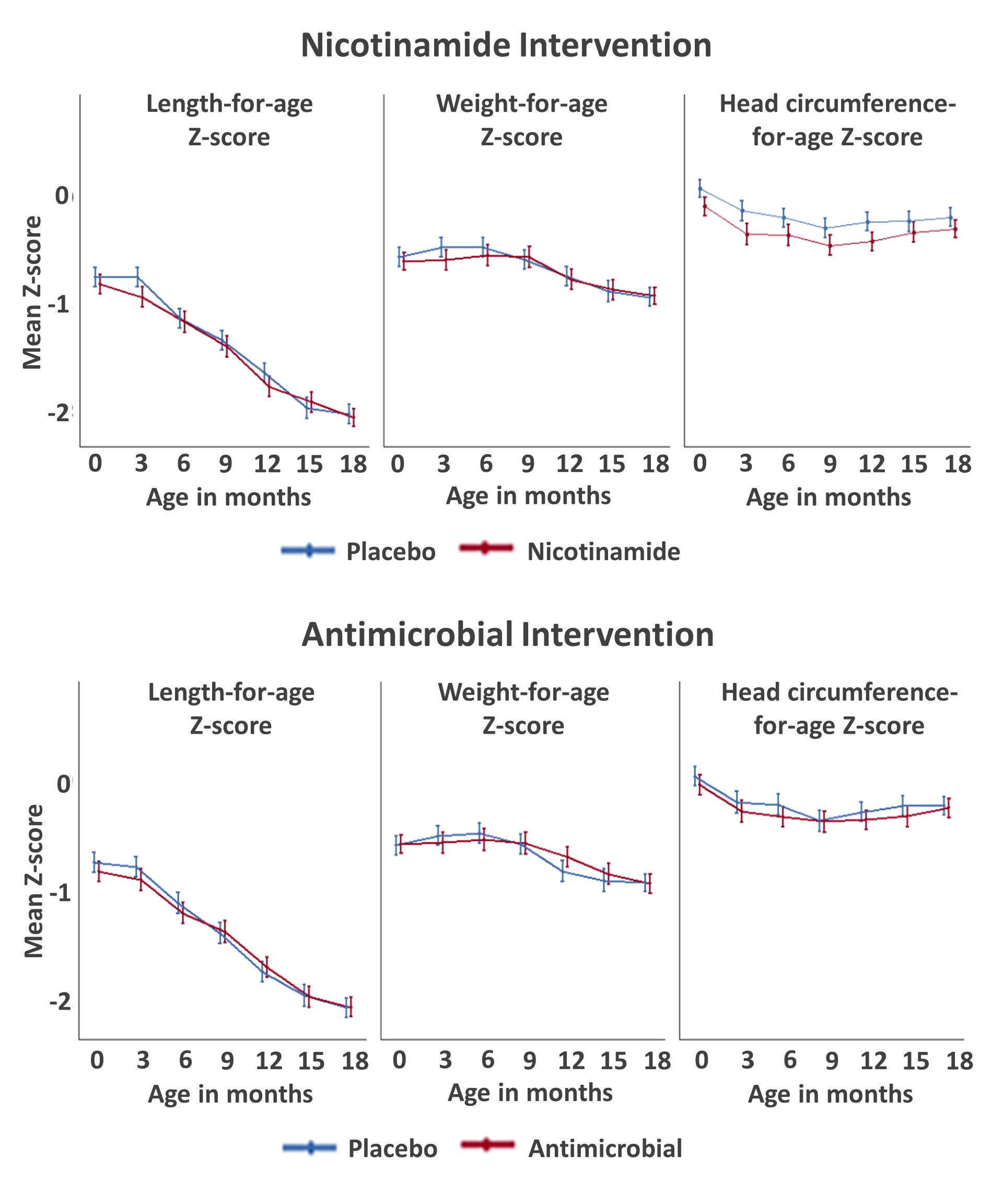
**
