# Supplementary material for: Effect of scheduled antimicrobial and nicotinamide treatment on linear growth in children in rural Tanzania: A factorial randomized, double-blind, placebo-controlled trial
Source: PLoS Med. 2021 Sep 28;18(9):e1003617. doi: 10.1371/journal.pmed.1003617 (PMC8478246; doi:10.1371/journal.pmed.1003617)

**S6 Fig A: Time to Severe Adverse Event (SAE) after enrollment by nicotinamide intervention arm.**


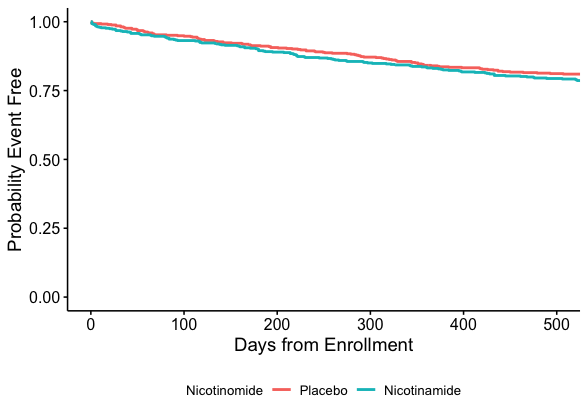


**S6 Fig B: Time to SAE after 6 months by antimicrobial intervention arm.**


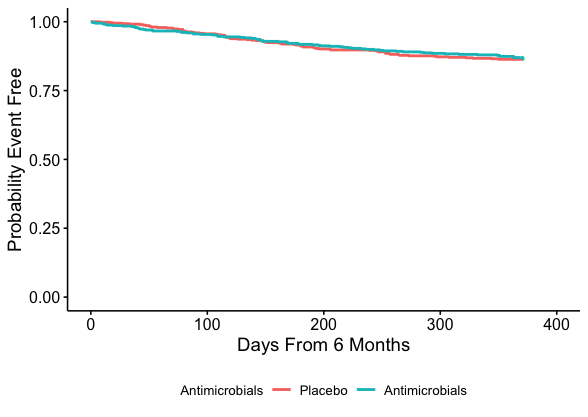

Supplement: S6 Fig — Time to SAE after enrollment by nicotinamide intervention arm (A) and antimicrobial intervention arm (B). SAE, serious adverse event. (DOCX) [file pmed.1003617.s010.docx]
