# Supplementary material for: Effect of scheduled antimicrobial and nicotinamide treatment on linear growth in children in rural Tanzania: A factorial randomized, double-blind, placebo-controlled trial
Source: PLoS Med. 2021 Sep 28;18(9):e1003617. doi: 10.1371/journal.pmed.1003617 (PMC8478246; doi:10.1371/journal.pmed.1003617)
Supplement: S1 Table — (DOCX) [file pmed.1003617.s011.docx]

**S1 Table: Baseline characteristics of the modified intention-to-treat group*.**

| Intervention | Nicotinamide | | Azithromycin + Nitazoxanide | |
| --- | --- | --- | --- | --- |
| Assignment | Placebo  (n =534) | Active  (n=550) | Placebo  (n =551) | Active  (n= 533) |
| *Infant factors* |  |  |  |  |
| Female sex | 263 (47.8%) | 260 (48.7%) | 263 (47.7%) | 260 (48.8%) |
| Hospital birth | 297 (54%) | 262 (49.1%) | 283 (51.4%) | 276 (51.8%) |
| Firstborn | 103 (19.2%) | 102 (19.5%) | 103 (18.7%) | 102 (19.1%) |
| Maternal age | 27.96 (6.93) | 27.84 (6.35) | 27.98 (6.65) | 27.82 (6.65) |
| Enrollment age (days) | 5.89 (3.57) | 5.88 (3.62) | 5.94 (3.53) | 5.84(3.66) |
| *Maternal/family factors* |  |  |  |  |
| Maternal height (cm) | 157.44 (5.52) | 157.18 (5.82) | 157.57 (5.6) | 157.05 (5.73) |
| Mother with ≥ 7 years of education | 420 (76.4%) | 395 (74%) | 428 (77.7%) | 387 (72.6%) |
| Monthly income (/1000TSH) | 48.9 (55.9) | 48.4 (46.3) | 49.2 (58.1) | 48.0 (43.3) |
| *Living environment factors* |  |  |  |  |
| Access to an improved drinking water source | 363 (66%) | 359 (67.2%) | 372 (67.5%) | 350 (65.7%) |
| Drinking water > 10 minutes from home | 448 (81.5%) | 431 (80.7%) | 454 (82.4%) | 425 (79.7%) |
| Access to an improved latrine | 59 (10.7%) | 59 (11%) | 63 (11.4%) | 55 (10.3%) |
| Crowding (>2 persons per room) | 120 (21.8%) | 114 (21.3%) | 107 (19.4%) | 127 (23.8%) |
| Agricultural land ownership | 533 (96.9%) | 519 (97.2%) | 533 (96.7%) | 519 (97.4%) |
| Water, Assets, Maternal Education, and Household Income Index score (Median, IQR)^**^ | 0.3 (0.1) | 0.3 (0.1) | 0.3 (0.1) | 0.3 (0.1) |
| *Anthropometry* |  |  |  |  |
| Enrollment length for age, measurement in cm (SD)  z-score (SD) | 49.03 (2.1)  -0.80 (1.01) | 49.07 (2.11)  -0.82 (1.02]) | 49.17 (2.07  -0.78 ± 1.01) | 48.93 (2.14)  -0.75 (1.02) |
| Enrollment weight for age, measurement in kg (SD)  z-score (SD) | 3.13 (0.47)  -0.61 (0.98) | 3.13 (0.49)  -0.60 (0.94) | 3.13 ± 0.48  -0.62 ± 1.02) | 3.13 (0.48)  -0.61 (0.99) |
| Enrollment head circumference for age,  measurement in cm (SD)  z-score (SD) | 34.64 (1.33)  -0.03 (1.01) | 34.8 (1.34)  -0.09 (1.03) | 34.78 ± 1.34  1.02 ± 0.998) | 34.66 (1.33)  0.01 (1.00) |

* Mean (standard deviation) is shown for continuous variables and number (percentage) for dichotomous variables unless otherwise stated.

** See reference 20.

Abbreviations: SD, standard deviation; TSH, Tanzania Shillings; IQR intraquartile range.
