# Supplementary material for: Effect of scheduled antimicrobial and nicotinamide treatment on linear growth in children in rural Tanzania: A factorial randomized, double-blind, placebo-controlled trial
Source: PLoS Med. 2021 Sep 28;18(9):e1003617. doi: 10.1371/journal.pmed.1003617 (PMC8478246; doi:10.1371/journal.pmed.1003617)
Supplement: S2 Table — (DOCX) [file pmed.1003617.s012.docx]

**S2 Table: Antimicrobial doses as part of intervention and other sources.**

|  |  | **Antimicrobial intervention** | |
| --- | --- | --- | --- |
| **Antimicrobial use—intervention (proportion) n=1091** | **Overall (n=1091)** | **Placebo (n=551)** | **Active (n=540)** |
| Received all doses of azithromycin intervention,  n (%) | 942 (86.3) | 465 (84.4) | 477 (88.3) |
| Missed 1 dose of azithromycin intervention,  n (%) | 143 (13.1) | 83 (15.1) | 60/540 (11.1) |
| Missed 2 doses of azithromycin intervention,  n (%) | 6 (0.6) | 3 (0.5) | 3 (0.6) |
| Received all doses of nitazoxanide intervention,  n (%) | 993 (91.0) | 496 (90.0) | 497 (92.0) |
| Missed 1 dose of nitazoxanide intervention,  n (%) | 98 (9.0) | 55 (10.0) | 43 (8.0) |
| **Other antimicrobial use (n=1091)** |  |  |  |
| Courses of any antimicrobial,  n (incidence per person year) | 2729 (6.99) | 1426 (7.25) | 1303 (6.72) |
| Courses of macrolides,  n (incidence per person year) | 214 (0.55) | 107 (0.54) | 107 (0.55) |
| Courses of penicillins,  n (incidence per person year) | 1589 (4.07) | 817 (4.15) | 772 (3.99) |
| Courses of other sulfonamides,  n (incidence per person year) | 250 (0.64) | 145 (0.74) | 105 (0.54) |
