# Supplementary material for: Effect of scheduled antimicrobial and nicotinamide treatment on linear growth in children in rural Tanzania: A factorial randomized, double-blind, placebo-controlled trial
Source: PLoS Med. 2021 Sep 28;18(9):e1003617. doi: 10.1371/journal.pmed.1003617 (PMC8478246; doi:10.1371/journal.pmed.1003617)
Supplement: S3 Table — (DOCX) [file pmed.1003617.s013.docx]

**S3 Table: Breast feeding and nicotinamide frequency.**

| **Breast feeding at 6 month visit (n=1141)** | Overall | Placebo  (n =578) | Nicotinamide  (n=563) |
| --- | --- | --- | --- |
| Months of exclusive breast feeding, mean, standard deviation* | 5.075 ± 1.102 | 5.07 ± 1.09 | 5.08 ± 1.12 |
| Exclusive breast feeding up to 6 month visit, n (%) | 594 (52.1) | 294 (50.9) | 300 (53.3) |
| Predominant breast feeding up to 6 month visit, n (%)* | 517 (45.3) | 269 (46.5) | 248 (44.1) |
| Some breast feeding (less than predominant) up to 6 month visit, n (%) | 29 (2.5) | 14 (2.4) | 15 (2.7 ) |
| **Nicotinamide use—intervention (n=1091)** |  | N=555 | N=536 |
| *Maternal nicotinamide (months 0-6)* |  |  |  |
| Missed < 20%, n (%) | 836 (76.6) | 419 (75.5) | 417 (77.8) |
| Missed between 20% & 50% of maternal nicotinamide by pill counting (months 0-6), n (%) | 231 (21.2) | 122 (22.0) | 109 (20.4) |
| Missed > 50% of maternal nicotinamide by pill counting (months 0-6), n (%) | 24 (2.2) | 14 (2.5) | 10 (1.8) |
| *Child nicotinamide (months 6-18)* |  |  |  |
| Missed <20%, n (%) | 699 (64.1) | 359 (64.7) | 340 (63.4) |
| Missed between 20% & 50% of child’s nicotinamide by sachet counting (months 6-18), n (%) | 368 (33.7) | 186 (33.5) | 182 (34.0) |
| Missed > 50% of child’s nicotinamide by sachet counting (months 6-18), n (%) | 24 (2.2) | 10 (1.8) | 14 (2.6) |

***** Breast feeding status was assessed monthly. Exclusive breastfeeding status over the 6 month period was defined as mother each month reporting current breastfeeding and answering “no” to questions whether mother has given any liquid or solids to the diet within the last month. Predominant breastfeeding was defined as mother ever reporting current breastfeeding and answering “yes” to whether breastfeeding was the main source of food and answering “yes” to having given any liquid or solids to the diet within the last month. Some breastfeeding (less than predominant) was defined as current breastfeeding but ever answering “no” to whether breastfeeding was the main source of food.
