# Supplementary material for: Effect of scheduled antimicrobial and nicotinamide treatment on linear growth in children in rural Tanzania: A factorial randomized, double-blind, placebo-controlled trial
Source: PLoS Med. 2021 Sep 28;18(9):e1003617. doi: 10.1371/journal.pmed.1003617 (PMC8478246; doi:10.1371/journal.pmed.1003617)
Supplement: S4 Table — (DOCX) [file pmed.1003617.s014.docx]

**S4 Table: Assessment for interaction between interventions on 18-month anthropometry outcomes.***

|  | **LAZ** |  | **WAZ** |  | **HCZ** |  | **MAZ** |  |
| --- | --- | --- | --- | --- | --- | --- | --- | --- |
| **Predictor** | **Estimate (95% CI)** | **p-value** | **Estimate (95% CI)** | **p-value** | **Estimate (95% CI)** | **p-value** | **Estimate (95% CI)** | **p-value** |
| Nicotinamide intervention | -0.01 (-0.16, 0.13) | 0.84 | -0.13 (-0.29, 0.03) | 0.11 | -0.13 (-0.29, 0.04) | 0.13 | -0.03 (-0.12, 0.19) | 0.67 |
| Antimicrobial intervention | 0.02 (-0.13, 0.16) | 0.84 | -0.12 (-0.28, 0.04) | 0.13 | -0.02 (-0.15, 0.18) | 0.85 | -0.04 (-0.19, 0.12) | 0.62 |
| Nicotinamide : Antimicrobial interaction | 0.11 (-0.09, 0.32) | 0.29 | 0.25 (-0.02, 0.48) | 0.03 | 0.17 (-0.09, 0.41) | 0.14 | 0.03 (-0.19, 0.25) | 0.76 |

* Shown are the results of multivariable regression for each of the anthropometry outcomes, assessing associations of the nicotinamide intervention (versus placebo), the antimicrobial intervention (versus placebo) and an interaction term for the two interventions, assessing whether there was a differential effect when the two were provided together.

Abbreviations: LAZ, length-for-age z-score; WAZ, weight-for-age z-score; HCZ, head-circumference-for-age z-score; MAZ, mid-upper arm circumference z-score; CI, confidence interval.
