# Supplementary material for: Effect of scheduled antimicrobial and nicotinamide treatment on linear growth in children in rural Tanzania: A factorial randomized, double-blind, placebo-controlled trial
Source: PLoS Med. 2021 Sep 28;18(9):e1003617. doi: 10.1371/journal.pmed.1003617 (PMC8478246; doi:10.1371/journal.pmed.1003617)
Supplement: S5 Table — (DOCX) [file pmed.1003617.s015.docx]

**S5 Table: Assessment for factors associated with 18-month outcomes to determine covariates for adjusted analysis.***

|  | **LAZ** | |  | **WAZ** | |  | **HCZ** | |  | **MUAC-Z** | |
| --- | --- | --- | --- | --- | --- | --- | --- | --- | --- | --- | --- |
| **Variable** | **Estimate** | **p-value** |  | **Estimate** | **p-value** |  | **Estimate** | **p-value** |  | **Estimate** | **p-value** |
|  |  |  |  |  |  |  |  |  |  |  |  |
| Female | 0.29 | 0.00 |  | 0.23 | 0.00 |  | 0.04 | 0.46 |  | 0.22 | 0.00 |
|  |  |  |  |  |  |  |  |  |  |  |  |
| First born | 0.03 | 0.76 |  | 0.14 | 0.07 |  | 0.02 | 0.83 |  | 0.14 | 0.08 |
|  |  |  |  |  |  |  |  |  |  |  |  |
| **WAMI vs. SES quartile** |  |  |  |  |  |  |  |  |  |  |  |
| SES2 | 0.07 | 0.45 |  | 0.08 | 0.40 |  | 0.04 | 0.65 |  | 0.06 | 0.54 |
| SES3 | 0.03 | 0.70 |  | -0.02 | 0.81 |  | 0.06 | 0.49 |  | -0.04 | 0.65 |
| SES4 | 0.34 | 0.00 |  | 0.26 | 0.00 |  | 0.26 | 0.00 |  | 0.24 | 0.00 |
|  |  |  |  |  |  |  |  |  |  |  |  |
| WAMI | 1.03 | 0.00 |  | 0.76 | 0.00 |  | 0.69 | 0.00 |  | 0.72 | 0.00 |
|  |  |  |  |  |  |  |  |  |  |  |  |
| **Mother’s features** |  |  |  |  |  |  |  |  |  |  |  |
| Height | 0.04 | 0.00 |  | 0.02 | 0.00 |  | 0.02 | 0.00 |  | 0.02 | 0.00 |
| Weight | 0.02 | 0.00 |  | 0.02 | 0.00 |  | 0.02 | 0.00 |  | 0.02 | 0.00 |
| Age | 0.00 | 0.85 |  | 0.00 | 0.80 |  | 0.01 | 0.07 |  | 0.00 | 0.90 |
| Datoga | 0.02 | 0.86 |  | -0.14 | 0.22 |  | 0.07 | 0.52 |  | -0.14 | 0.23 |
|  |  |  |  |  |  |  |  |  |  |  |  |
| **Schooling** |  |  |  |  |  |  |  |  |  |  |  |
| Mother’s years | 0.02 | 0.02 |  | 0.01 | 0.15 |  | 0.00 | 0.94 |  | 0.01 | 0.16 |
| Mother >7 years | 0.04 | 0.61 |  | 0.03 | 0.64 |  | -0.04 | 0.61 |  | 0.03 | 0.69 |
|  |  |  |  |  |  |  |  |  |  |  |  |
| **Birth month vs. season** |  |  |  |  |  |  |  |  |  |  |  |
| February | 0.08 | 0.51 |  | 0.11 | 0.34 |  | 0.00 | 0.98 |  | 0.12 | 0.31 |
| March | 0.20 | 0.09 |  | 0.31 | 0.01 |  | 0.09 | 0.44 |  | 0.32 | 0.01 |
| April | 0.26 | 0.04 |  | 0.27 | 0.03 |  | 0.21 | 0.10 |  | 0.28 | 0.03 |
| May | 0.16 | 0.22 |  | 0.12 | 0.33 |  | -0.20 | 0.12 |  | 0.14 | 0.29 |
| June | 0.17 | 0.26 |  | 0.15 | 0.30 |  | 0.10 | 0.49 |  | 0.16 | 0.26 |
| July | 0.17 | 0.23 |  | 0.10 | 0.46 |  | -0.06 | 0.66 |  | 0.08 | 0.54 |
| August | -0.16 | 0.29 |  | -0.14 | 0.34 |  | -0.18 | 0.24 |  | -0.12 | 0.43 |
| September | 0.25 | 0.23 |  | 0.10 | 0.61 |  | -0.05 | 0.82 |  | 0.11 | 0.59 |
| October | -0.22 | 0.14 |  | -0.13 | 0.37 |  | -0.19 | 0.19 |  | -0.09 | 0.54 |
| November | 0.12 | 0.40 |  | 0.03 | 0.83 |  | -0.17 | 0.23 |  | 0.03 | 0.80 |
| Decermber | -0.09 | 0.47 |  | 0.12 | 0.30 |  | 0.06 | 0.61 |  | 0.12 | 0.28 |
| Season | -0.05 | 0.45 |  | 0.03 | 0.61 |  | 0.05 | 0.38 |  | 0.02 | 0.72 |
|  |  |  |  |  |  |  |  |  |  |  |  |
| Hospital birth | 0.24 | <0.01 |  | 0.27 | <0.01 |  | 0.25 | <0.01 |  | 0.26 | <0.01 |
|  |  |  |  |  |  |  |  |  |  |  |  |
| **Ward:** |  |  |  |  |  |  |  |  |  |  |  |
| Gar | 0.04 | 0.78 |  | -0.10 | 0.43 |  | 0.23 | 0.09 |  | -0.10 | 0.44 |
| Geterer | -0.32 | 0.01 |  | -0.26 | 0.03 |  | 0.10 | 0.42 |  | -0.28 | 0.02 |
| Hayderer | 0.03 | 0.84 |  | 0.05 | 0.73 |  | 0.29 | 0.06 |  | 0.03 | 0.86 |
| Haydom | -0.03 | 0.80 |  | 0.06 | 0.60 |  | 0.35 | 0.00 |  | 0.03 | 0.77 |
| Maghang | -0.15 | 0.26 |  | -0.11 | 0.37 |  | 0.25 | 0.05 |  | -0.14 | 0.25 |
| Mwanga | 0.10 | 0.43 |  | 0.11 | 0.37 |  | 0.29 | 0.01 |  | 0.08 | 0.50 |
| Other | -0.55 | 0.22 |  | 0.49 | 0.26 |  | 1.52 | 0.00 |  | 0.47 | 0.29 |
|  |  |  |  |  |  |  |  |  |  |  |  |
| Age days | 0.00 | 0.05 |  | 0.00 | 0.09 |  | 0.00 | 0.53 |  | 0.00 | 0.06 |

* Shown are results of univariate analyses where the outcomes were the anthropometry measures (LAZ, WAZ, etc.) and the predictors were pre-specified variables that represented potential confounders. As part of the pre-specified statistical analysis plan, each of the above variables with an effect size of >0.2 z-scores or p<0.2 for a particular anthropometry measure was included as a confounder in the adjusted multivariable regression model for that anthropometry outcome.

Abbreviations: LAZ, length-for-age z-score; WAZ, weight-for-age z-score; HCZ, head-circumference-for-age z-score; MUAC, mid-upper arm circumference; SES, socioeconomic status; WAMI, water and sanitation, assets, maternal education and household income.
