# Supplementary material for: Effect of scheduled antimicrobial and nicotinamide treatment on linear growth in children in rural Tanzania: A factorial randomized, double-blind, placebo-controlled trial
Source: PLoS Med. 2021 Sep 28;18(9):e1003617. doi: 10.1371/journal.pmed.1003617 (PMC8478246; doi:10.1371/journal.pmed.1003617)
Supplement: S6 Table — (DOCX) [file pmed.1003617.s016.docx]

**S6 Table: Assessment of 18-month LAZ adjusted for all covariates listed in Supplementary Table 5 (regardless of their relationship to LAZ from pre-specified selection analysis).**

|  | **Nicotinamide** | |  | **Antimicrobial** | |  |
| --- | --- | --- | --- | --- | --- | --- |
| Variable | **Placebo** | **Active** | Difference in z-scores, adjusted for all covariates (CI) | **Placebo** | **Active** | Difference in z-scores, adjusted for all covariates (CI) |
| **Modified Intention to Treat Group** |  |  |  |  |  |  |
| Length, z-score (SD)  measurement in cm (SD)  (n=1,084) | -2.04 (0.95)  75.9 (2.79) | -2.06 (1.04)  76.0 (2.94) | 0.04 (-0.07, 0.14) | -2.05 (1.01)  76.0 (2.89) | -2.05 (0.99)  75.9 (2.83) | 0.08 (-0.03, 0.18) |
| Weight, z-score (SD)  measurement in kg (SD)  (n=1,080) | -0.94 (0.94)  9.60 (1.10) | -0.94 (0.99)  9.60 (1.10) | -0.01 (-0.11, 0.10) | -0.93 (0.95)  9.61 (1.08) | -0.94 (0.99)  9.59 (1.09) | 0.00 (-0.10, 0.11) |
| Head circ., z-score (SD)  measurement in cm (SD)  (n=1,083) | -0.31 (0.98)  46.4 (1.44) | -0.20 (0.96)  46.6 (1.40) | 0.04 (-0.06, 0.13) | -0.23 (0.98)  46.5 (1.47) | -0.28 (0.96)  46.5 (1.38) | 0.01 (-0.09, 0.10) |
| MUAC, z-score (SD)  measurement in cm (SD)  (n=1,078) | 0.17 (0.89)  14.9 (1.07) | 0.15 (0.94)  14.9 (1.13) | -0.02 (-0.13, 0.09) | 0.14 (0.91  14.9 (1.09) | 0.19 (0.93)  14.9 (1.11) | 0.06 (-0.04, 0.17) |
| **Per Protocol Group** |  | |  |  |  |  |
| Variable | **Placebo** | **Active** | Difference in z-scores, adjusted for all covariates (CI) | **Placebo** | **Active** | Difference in z-scores, adjusted for all covariates (CI) |
| Length-for-age z-score  (measurement in cm)  (n=1,018) | -2.08 (75.8) | -2.04 (76.0) | 0.04 (-0.07, 0.15) | -2.07 (75.9) | -2.05 (75.9) | 0.07 (-0.04, 0.18) |
| Weight-for-age z-score  (measurement in kg)  (n=1,014) | -0.94 (9.59) | -0.94 (9.60) | 0.02 (-0.13, 0.09) | -0.94 (9.60) | -0.94 (9.59) | 0.01 (-0.10, 0.12) |
| Head circ.-for-age z-score  (measurement in cm)  (n=1,017) | -0.31 (46.4) | -0.20 (46.6) | 0.02 (-0.08, 0.12) | -0.25 (46.5) | -0.26 (46.5) | 0.03 (-0.07, 0.12) |
| MUAC-for-age z-score  (measurement in cm)  (n=1,013) | -0.18 (14.9) | 0.15 (14.90) | -0.04 (-0.15, 0.07) | 0.13 (14.9) | 0.20 (15.0) | 0.08 (-0.03, 0.20) |

All analyses adjusted for corresponding anthropometry at baseline, age at anthropometry measure, sex, ward, hospital birth, firstborn status, SES, birth month, years of maternal education, maternal age, whether or not the mother was a member of the Datoga tribe, the mothers height and weight.

Abbreviations: CI confidence interval; MUAC, mid-upper arm circumference.
