# Supplementary material for: Effect of scheduled antimicrobial and nicotinamide treatment on linear growth in children in rural Tanzania: A factorial randomized, double-blind, placebo-controlled trial
Source: PLoS Med. 2021 Sep 28;18(9):e1003617. doi: 10.1371/journal.pmed.1003617 (PMC8478246; doi:10.1371/journal.pmed.1003617)
Supplement: S7 Table — (DOCX) [file pmed.1003617.s017.docx]

**S7 Table: Sensitivity analysis of 18-month anthropometry outcomes for those whose 18-month anthropometry was measured in 18-month window.**

|  | **Nicotinamide** | |  |  |  |  |
| --- | --- | --- | --- | --- | --- | --- |
| **Variable** | **Placebo** | **Active** | **Difference, unadjusted (CI)** | **p-value** | **Difference, adjusted (CI)** | **p-value** |
| Length, z-score  (measurement in cm)  (n=853) | -2.04 (75.7) | -2.00 (75.82) | 0.04 (-0.17, 0.10) | 0.61 | 0.04 (-0.07, 0.16) | 0.47 |
| Weight z-score  (measurement in kg)  (n=853) | -0.92 (9.57) | -0.93 (9.56) | 0.01 (-0.11, 0.15) | 0.80 | \| -0.01 (-0.13, 0.11) \| \| --- \| | 0.86 |
| Head circ., z-score  (measurement in cm)  (n=852) | -0.30 (46.4) | -0.19 (46.5) | 0.11 (-0.24, 0.03) | 0.11 | 0.04 (-0.08, 0.15) | 0.52 |
| MUAC, z-score  (measurement in cm)  (n=847) | 0.19 (14.9) | 0.11 (14.834) | 0.08 (-0.04, 0.20) | 0.19 | -0.08 (-0.20, 0.04) | 0.20 |
|  | **Antimicrobial** | |  |  |  |  |
| **Variable** | **Placebo** | **Active** | Difference, unadjusted | p-value | Difference, adjusted | p-value |
| Length, z-score  (measurement in cm)  (n=853) | -2.0 (75.8) | -2.0 (75.7) | 0.00 (-0.13, 0.14) | 0.98 | 0.07 (-0.05, 0.19) | 0.24 |
| Weight z-score  (measurement in kg)  (n=853) | -0.91 (9.59) | -0.94 (9.53) | 0.03 (-0.09, 0.17) | 0.57 | 0.01 (-0.11, 0.13) | 0.86 |
| Head circ., z-score  (measurement in cm)  (n=852) | -0.2 (46.5) | -0.3 (46.41) | 0.06 (-0.08, 0.19) | 0.41 | 0.02 (-0.10, 0.13) | 0.78 |
| MUAC, z-score  (measurement in cm)  (n=847) | 0.1 (14.9) | 0.2 (14.88) | 0.01 (-0.14, 0.11) | 0.82 | 0.05 (-0.07, 0.17) | 0.45 |

* Adjustments based on baseline measures and these individual covariates being associated with final outcome:

LAZ: baseline LAZ, ward, hospital birth, years of maternal education, SES quartile, sex, and mother’s height and weight

WAZ: baseline WAZ, ward, firstborn status, hospital birth, years of maternal education, SES quartile, sex, mother’s height and weight

HCZ: baseline HCZ, ward, hospital birth, SES quartile, mother’s height, weight, and age

MUAC: baseline WAZ,(baseline MUAC not available for most participants), ward, firstborn status, hospital birth, years of maternal education, SES quartile, sex, mother’s height and weight

Abbreviations: LAZ, length-for-age z-score; WAZ, weight-for-age z-score; HCZ, head-circumference-for-age z-score; MUAC, mid-upper arm circumference; CI, confidence interval.
