# Supplementary material for: Effect of scheduled antimicrobial and nicotinamide treatment on linear growth in children in rural Tanzania: A factorial randomized, double-blind, placebo-controlled trial
Source: PLoS Med. 2021 Sep 28;18(9):e1003617. doi: 10.1371/journal.pmed.1003617 (PMC8478246; doi:10.1371/journal.pmed.1003617)
Supplement: S8 Table — (DOCX) [file pmed.1003617.s018.docx]

**S8 Table: 18-month anthropometry outcomes from per-protocol analysis.**

|  | **Nicotinamide** | |  |  |  |  |
| --- | --- | --- | --- | --- | --- | --- |
| **Variable** | **Placebo** | **Active** | **Difference, unadjusted (CI)** | **p-value** | **Difference, adjusted* (CI)** | **p-value** |
| Length-for-age z-score  (measurement in cm)  (n=1,018) | -2.08 (75.8) | -2.04 (76.0) | 0.04 (-0.17, 0.08) | 0.50 | 0.04 (-0.06, 0.15) | 0.43 |
| Weight-for-age z-score  (measurement in kg)  (n=1,014) | -0.94 (9.59) | -0.94 (9.60) | 0.00 (-0.12, 0.12) | 0.98 | 0.00 (-0.11, 0.11) | 0.95 |
| Head circ.-for-age z-score  (measurement in cm)  (n=1,017) | -0.31 (46.4) | -0.20 (46.6) | 0.11 (-0.23, 0.01) | 0.07 | 0.05 (-0.06, 0.14) | 0.31 |
| MUAC-for-age z-score  (measurement in cm)  (n=1,013) | -0.18 (14.9) | 0.15 (14.90) | 0.03 (-0.18, 0.04) | 0.23 | -0.04 (-0.15, 0.07) | 0.51 |
|  | **Antimicrobial** | |  |  |  |  |
| **Variable** | **Placebo** | **Active** | Difference, unadjusted | p-value | Difference, adjusted* | p-value |
| Length-for-age z-score  (measurement in cm)  (n=1,018) | -2.07 (75.9) | -2.05 (75.9) | 0.02 (-0.13, 0.11) | 0.85 | 0.06 (-0.04, 0.17) | 0.25 |
| Weight-for-age z-score  (measurement in kg)  (n=1,014) | -0.94 (9.60) | -0.94 (9.59) | 0.00 (-0.12, 0.12) | 0.99 | 0.01 (-0.09, 0.12) | 0.80 |
| Head circ.-for-age z-score  (measurement in cm)  (n=1,017) | -0.25 (46.5) | -0.26 (46.5) | 0.01 (-0.10, 0.14) | 0.77 | 0.03 (-0.07, 0.13) | 0.55 |
| MUAC-for-age z-score  (measurement in cm)  (n=1,013) | 0.13 (14.9) | 0.20 (15.0) | 0.07 (-0.18, 0.04) | 0.27 | 0.08 (-0.03, 0.19) | 0.13 |

* Adjustments based on baseline measures and these individual covariates being associated with final outcome:

LAZ: baseline LAZ, age in days at 18-month measurement, ward, hospital birth, birth month, years of maternal education, SES quartile category, sex, whether the mother was a member of the Datoga tribe, and mother’s height and weight

WAZ: baseline WAZ, age in days at 18-month measurement, ward, firstborn status, hospital birth, birth month, years of maternal education, SES quartile category, sex, mother’s height and weight

HCZ: baseline HCZ, ward, hospital birth, birth month, SES quartile category, mother’s height, weight, and age

MUAC: baseline WAZ (baseline MUAC not available for most participants), age at 18-month measurement, ward, firstborn status, hospital birth, birth month, years of maternal education, SES quartile category, sex, mother’s height and weight

Abbreviations: LAZ, length-for-age z-score; WAZ, weight-for-age z-score; HCZ, head-circumference-for-age z-score; MUAC, mid-upper arm circumference; CI, confidence interval.
