# Supplementary material for: Effect of scheduled antimicrobial and nicotinamide treatment on linear growth in children in rural Tanzania: A factorial randomized, double-blind, placebo-controlled trial
Source: PLoS Med. 2021 Sep 28;18(9):e1003617. doi: 10.1371/journal.pmed.1003617 (PMC8478246; doi:10.1371/journal.pmed.1003617)
Supplement: S9 Table — (DOCX) [file pmed.1003617.s019.docx]

**S9 Table: Primary outcome weighted to account for potential differential death or loss-to-follow-up by intervention group.**

|  | **Nicotinamide** | |  |  |  |  |
| --- | --- | --- | --- | --- | --- | --- |
| Variable | **Placebo** | **Active** | Difference in z-scores, unadjusted (CI) | p-value | Difference in z-scores, adjusted | p-value |
| Length, z-score (SD)  measurement in cm (SD)  (n=1,084) | -2.04 (0.95)  75.9 (2.79) | -2.06 (1.04)  76.0 (2.94) | 0.03 (-0.09, 0.15) | 0.67 | 0.04 (-0.07, 0.14) | 0.47 |
|  | **Antimicrobial** | |  |  |  |  |
| Variable | **Placebo** | **Active** | Difference in z-scores,  unadjusted | p-value | Difference in z-scores,  adjusted | p-value |
| Length, z-score (SD)  measurement in cm (SD)  (n=1084) | -2.05 (1.01)  76.0 (2.89) | -2.05 (0.99)  75.9 (2.83) | 0.02 (-0.10, 0.14) | 0.80 | 0.08 (-0.02, 0.18) | 0.13 |

Models for adjusted analyses include baseline LAZ, age in days at 18-month measurement, ward, hospital birth, birth month, years of maternal education, SES quartile category, sex, whether the mother was a member of the Datoga tribe, and mother’s height.

Abbreviations: LAZ, length-for-age z-score; SD, standard deviation.
