# Supplementary material for: Effect of scheduled antimicrobial and nicotinamide treatment on linear growth in children in rural Tanzania: A factorial randomized, double-blind, placebo-controlled trial
Source: PLoS Med. 2021 Sep 28;18(9):e1003617. doi: 10.1371/journal.pmed.1003617 (PMC8478246; doi:10.1371/journal.pmed.1003617)
Supplement: S10 Table — (DOCX) [file pmed.1003617.s020.docx]

**S10 Table: Stunting prevalence by time point.**

|  | **Nicotinamide** | |  | **Antimicrobial** | |  |
| --- | --- | --- | --- | --- | --- | --- |
| **Time point** | **Placebo** | **Active** | **p-value** | **Placebo** | **Active** | **p-value** |
| Baseline | 60/550 (10.9%) | 63/534 (11.8%) | 0.71 | 57/551 (10.3%) | 66/533 (12.4%) | 0.34 |
| 3 month | 85/549 (15.5%) | 64/533 (12.0%) | 0.12 | 66/550 (12.0%) | 83/532 (15.6%) | 0.10 |
| 6 month | 127/550 (23.1%) | 131/534 (24.5%) | 0.63 | 125/551 (22.7%) | 133/533 (25.0%) | 0.42 |
| 9 month | 151/530 (28.5%) | 145/518 (28.0%) | 0.91 | 147/529 (27.8%) | 149/519 (28.7%) | 0.79 |
| 12 month | 203/550 (36.9%) | 166/533 (31.1%) | 0.05 | 184/550 (33.5%) | 185/533 (34.7%) | 0.71 |
| 15 month | 194/515 (37.7%) | 166/533 (31.1%) | 0.92 | 200/522 (38.3%) | 182/499 (34.7%) | 0.59 |
| 18 month | 285/550 (51.8%) | 248/534 (46.4%) | 0.09 | 267/551 (48.5%) | 266/533 (49.9%) | 0.67 |
