# Supplementary material for: Effect of scheduled antimicrobial and nicotinamide treatment on linear growth in children in rural Tanzania: A factorial randomized, double-blind, placebo-controlled trial
Source: PLoS Med. 2021 Sep 28;18(9):e1003617. doi: 10.1371/journal.pmed.1003617 (PMC8478246; doi:10.1371/journal.pmed.1003617)
Supplement: S11 Table — (DOCX) [file pmed.1003617.s021.docx]

**S11 Table: Safety laboratory study information.**

|  |  | **Placebo** | | **Nicotinamide** | |
| --- | --- | --- | --- | --- | --- |
|  | **Month** | **Mean (95% confidence interval)** | **Number abnormal (percent)** | **Mean (95% confidence interval)** | **Number abnormal (percent)** |
| Sodium | 2 | 136 (130.8, 141.2) | 12/44 (27.2) | 133.9 (133.4, 134.5) | 12/47 (25.5) |
| (mmol/L) | 8 | 133.7 (132.8, 134.7) | 4/51 (7.8) | 131.6 (126.8, 136.3) | 15/56 (26.8) |
|  | 18 | 139.2 (137.5, 140.8) | 27/72 (37.5) | 137.6 (135.9, 139.2) | 15/58 (25.9) |
| Chloride | 2 | 99.7 (95.2, 104.2) | 5/44 (11.4) | 102.4 (101.8, 103.1) | 3/47 (6.4) |
| (mmol/L) | 8 | 103.8 (103.3, 104.4) | 2/51 (3.9) | 101.4 (97.7, 105) | 1/56 (1.8) |
|  | 18 | 106.7 (105.1, 108.3) | 14/72 (19.4) | 105.3 (103.7, 106.9) | 16/58 (27.6) |
| Bicarbonate | 2 | 17.6 (17, 18.2) | 0/44 (0) | 17.9 (17.4, 18.4) | 0/47 (0) |
| (mmol/L) | 8 | 15.9 (15.1, 16.6) | 4/51 (7.8) | 16.5 (15.7, 17.3) | 0/56 (0) |
|  | 18 | 19 (18.1, 19.8) | 0/35 (0) | 18.3 (17.3, 19.3) | 7/58 (12.1) |
| Creatinine | 2 | 29.4 (26.1, 32.7) | 16/44 (36.3) | 28.5 (24.6, 32.4) | 17/47 (36.2) |
| (mmol/L) | 8 | 15.9 (14.7, 17.1) | 1/51 (2.0) | 16.4 (15.1, 17.7) | 0/56 (0) |
|  | 18 | 22.1 (20.4, 23.8) | 10/72 (13.9) | 22.2 (20.8, 23.7) | 6/58 (10.3) |
| BUN | 2 | 1.9 (1.5, 2.3) | 0/44 (0) | 1.6 (1.4, 1.8) | 0/46 (0) |
| (mmol/L) | 8 | 1.7 (1.5, 1.8) | 0/51 (0) | 1.6 (1.5, 1.8) | 1/56 (1.8) |
|  | 18 | 2.4 (2.2, 2.6) | 4/72 (5.6) | 2.6 (2.3, 2.8) | 7/58 (12.1) |
| ALT | 2 | 23.7 (19.7, 27.8) | 5/44 (11.4) | 27.5 (21, 34.1) | 4/47 (8.5) |
| (U/L) | 8 | 17.3 (15.8, 18.9) | 0/49 (0) | 20.3 (18, 22.6) | 4/55 (7.3) |
|  | 18 | 33.4 (17.3, 49.6) | 14/72 (19.4) | 24.4 (20.8, 27.9) | 8/58 (13.8) |
| AST | 2 | 50.5 (45.7, 55.3) | 5/39 (12.8) | 54.2 (47, 61.5) | 6/43 (14.0) |
| (U/L) | 8 | 49.4 (45, 53.9) | 2/49 (4.1) | 55.8 (50.8, 60.7) | 9/55 (16.4) |
|  | 18 | 51.1 (43.8, 58.4) | 8/72 (11.1) | 49.9 (43, 56.9) | 6/58 (10.3) |
| Bilirubin | 2 | 7.9 (5.8, 10) | 2/39 (5.1) | 6.4 (5.1, 7.7) | 0/43 (0) |
| (umol/L) | 8 | 2.7 (2.3, 3.1) | 0/51 (0) | 2.2 (1.9, 2.5) | 0/56 (0) |
|  | 18 | 2.2 (1.9, 2.5) | 0/67 (0) | 2 (1.7, 2.3) | 0/53 (0) |
| Hemoglobin | 2 | 12.2 (11.7, 12.7) | 12/45 (26.7) | 12 .0(11.7, 12.3) | 14/48 (29.2) |
| (g/dL) | 8 | 10.8 (10.5, 11.1) | 43/60 (71.7) | 11.3 (11,0 11.5)* | 34/57 (60.0) |
|  | 18 | 12.2 (11.7, 12.7) | 14/52 (26.9) | 12.3 (11, 13.6) | 26/60 (43.3) |
| Hematocrit | 2 | 34.2 (32.7, 35.7) | 21/45 (47.7) | 33.9 (32.8, 35) | 29/48 (60.4) |
| (%) | 8 | 31.8 (30.9, 32.7) | 8/60 (13.3) | 33.4 (32.6, 34.1)* | 2/57 (3.5) |
|  | 18 | 34.0 (32.7, 35.3) | 15/52 (28.9) | 33.0 (31.3, 34.6) | 28/61 (45.9) |
| WBC | 2 | 6 (5.3, 6.7) | 5/45 (11.1) | 6.9 (6.1, 7.6) | 8/48 (16.7) |
| (1000 cells/uL) | 8 | 9.3 (8.5, 10) | 30/60 (50.0) | 10.2 (9.3, 11.2) | 29/57 (50.8) |
|  | 18 | 6.1 (5.5, 6.8) | 4/52 (7.7) | 7.2 (5.6, 8.7) | 18/60 (30.0) |
| Platelets | 2 | 311.7 (276.4, 346.9) | 8/45 (17.8) | 307.7 (273.6, 341.7) | 6/48 (12.5) |
| (platelets/uL) | 8 | 338.6 (302.3, 374.9) | 9/60 (15.0) | 349.6 (308.3, 390.9) | 13/57 (22.8) |
|  | 18 | 313.1 (279.5, 346.6) | 22/52 (42.3) | 314.5 (281.5, 347.4) | 26/60 (43.3) |

* p<0.05 in t-test comparing nicotinamide group to placebo group.

Abbreviations: BUN, blood urea nitrogen; ALT, alanine aminotransaminase; AST; aspartate aminotransaminase; WBC, white blood cell count.
